# A Comparative Study of Cabernet Sauvignon Red Wine Aroma Profiles During Ageing in Medium-Toasted Oak Barrels

**DOI:** 10.3390/foods14183178

**Published:** 2025-09-12

**Authors:** Anita Pichler, Ivana Ivić, Josip Mesić, Brankica Svitlica, Nela Nedić Tiban, Iva Ostrun, Tanja Marković, Mirela Kopjar

**Affiliations:** 1 Faculty of Food Technology Osijek, Josip Juraj Strossmayer University, F. Kuhača 18, 31000 Osijek, Croatia; anita.pichler@ptfos.hr (A.P.); nela.nedic@ptfos.hr (N.N.T.); iostrun@ptfos.hr (I.O.); mirela.kopjar@ptfos.hr (M.K.); 2 Faculty of Tourism and Rural Development, Vukovarska 17, 34000 Požega, Croatia; jmesic@ftrr.hr; 3 Faculty of Agrobiotechnical Sciences Osijek, Josip Juraj Strossmayer University, V. Preloga 1, 31000 Osijek, Croatia; bsvitlica@fazos.hr; 4 Institute of Public Health, Franje Krežme 1, 31000 Osijek, Croatia; tanja.markovic.zzjz@gmail.com

**Keywords:** cabernet sauvignon, oak barrel, medium toasting, ageing, aroma profile

## Abstract

Ageing in oak barrels affects the tertiary aroma of red wine, yet further research on the impact of different conditions used for medium toasting of barrels could still be conducted to optimise wine production and meet consumer preferences. In this study, using the GC/MS method, the aroma profiles of two consecutive vintages of Cabernet Sauvignon wine and samples aged for 12 months in different vessels were determined. Besides the stainless steel tanks, Excellence barrels with medium, medium plus, and medium long toasting, and Premium barrels with medium toasting were used. A total of 48 aroma compounds were identified, and their odour activity value (OAV ≥ 1) was calculated. According to it, 10 key compounds were selected: β-damascenone, ethyl octanoate, ethyl vanillate, ethyl cinnamate, lauric acid, linalool, hotrienol, ethyl hexanoate, diethyl succinate, and 2-phenylethanol. The results showed that wooden barrels have a greater impact on wine aroma during ageing, compared to stainless steel tanks. The initial wine aroma and key compounds with fruity, floral, and fatty notes were most preserved in the stainless steel tank. The study highlights that controlled selection of barrel type (grain density) and toasting method can significantly modulate the aromatic complexity of Cabernet Sauvignon wines. Toasted barrels resulted in an increase in smoky and woody notes (volatile phenols and lactones), and a decrease in fruity and floral notes (β-damascenone, 2-phenylethanol, hotrienol, linalool, etc.). These findings could provide practical guidelines for winemakers for optimising ageing strategies and contributing new insights into the impact of wine vessels on aroma development and sensory perception.

## 1. Introduction

Cabernet Sauvignon is one of the most widely grown grape varieties, and its wine is valued due to its rich aroma profile, full body, pronounced tannins, and the ability to age for a long period [1]. The aroma profile of Cabernet Sauvignon wine is complex and usually includes compounds with fruity, spicy, and smoky notes [2]. Volatile compounds play a crucial role in wine quality and consumer acceptance of wine. These compounds in wine originate from grapes (primary aroma, including monoterpenes, like linalool and hotrienol), developed during alcoholic and malolactic fermentation (secondary aroma, including higher alcohols, volatile acids, and esters) and the ageing process (tertiary aroma, including various compounds, and depending on ageing vessel, time, etc.) [3,4,5,6]. The final aroma of the wine is a combination of the above-mentioned factors.

Ageing of red wine represents an essential stage when the final aroma profile of the wine is formed and shaped, and therefore, the choice of ageing vessel has a significant influence on the final product [6,7,8]. During ageing and maturation of wine, the contact and/or reaction of wine volatile compounds with the ageing vessel results in a loss of some compounds, but also in forming new ones. For example, the non-aromatic glycosidically bound terpenes, present in grapes, go through enzyme hydrolysis, releasing new free aromatic compounds [9]; higher alcohols and fatty acids are potential precursors of esters; microoxygenation or extraction from wood surface results in the formation of new aromatic compounds, etc. [10]. This greatly depends on the ageing vessel type, volume, and material. Stainless steel tanks are durable, easy to maintain and manage; however, they are inert, they do not interact with the wine during ageing, nor do they alter or contribute to its aroma [11]. On the other hand, ageing in wooden barrels is a complex process that results in different changes in the wine aroma profile. The wood can release aroma compounds in wine, react with the wine, and change its final aroma profile [12].

Different types of wood can be used for barrel production, like white or red oak, chestnut oak, redwood, mulberry, and others. The choice depends on the wood characteristics, like porosity, hardness, bending, and contribution to wine, etc. To date, the most suitable and affordable type of wood for barrel production is still oak [8,13]. One of the most important properties of wooden barrels is porosity, which enables microoxygenation, a key process where small amounts of oxygen pass through the wood pores and react with wine aroma compounds. This results in the formation of a unique aroma profile of wine, making ageing in wooden barrels the focus of many studies [8,14,15,16,17].

However, the properties of the wood are not the only factors that influence wine aroma during ageing in wooden barrels. Other factors include ageing time, cellar temperature, barrel size and volume, its age and number of uses, grain density of the wood, toasting level, and others. The barrel volume influences the ratio of wood surface/wine volume, and this is smaller if the barrel is larger. Further, ageing time depends on wine variety and its composition, and also on all the above-mentioned factors [17].

A significant influence has been attributed to the toasting level of the wooden barrels. Toasting represents a process of controlled heating of the inside of barrels, which results in chemical changes in wood properties. High temperatures during toasting result in the formation of various aroma compounds that can pass from wood to wine. These compounds are mostly desirable and have spicy, vanilla, coffee, and smoky aromatic notes. If the toasting temperature is too high or the toasting time is too long, thermodegradation of the mentioned compounds could occur. There are three main toasting levels: light, medium, and heavy toasting, and they are conducted at different temperatures and times. However, each toasting level can be conducted at a certain temperature and time intervals, so different variations or intensities have been reported [18,19,20]. Light toasting includes temperatures from 120 to 180 °C, held at the highest temperature up to 5 min, suitable for wine where minimal changes are necessary. For vanilla and spicy notes, softer tannins and a more complex aroma profile, medium toasting is used (180–210 °C, from 5 to 10 min or even longer at the highest temperature). Heavy toasting is conducted at temperatures up to 230 °C (for 5 to 15 min), resulting in coffee and deep smoky aromatic notes in wine [8].

Toasted oak barrel ageing significantly affects the aroma profile of red wine, as mentioned. While most previous studies focused on the main toasting levels (light, medium, and heavy), recent findings highlight that even subtle variations within the same toasting level can significantly influence the aroma profile of wines [8,17,20,21,22,23]. Nevertheless, a medium toasting level of oak barrels has been proven to be most suitable for most wines, and it is the focus of many studies due to its ability to obtain an optimised aroma profile with vanilla, coconut, smoky, and spicy notes. Previous studies included changes in wine characteristics affected by main toasting levels [18], the volume of medium-toasted barrels [24], medium-toasted barrels obtained from different cooperages [25], medium-toasted oak chips added into wine [26], and the type of wood [27] or the origin of wood [28] used for medium-toasted barrels production. Some studies focused on certain compounds like volatile phenols and lactones [20], ellagitannins [19,29], or specific alcohols and esters [30]. However, it can be observed that little attention has been given to the fine subdivision of medium toasting parameters and their impact on key aroma compounds. In the Croatian wine industry, oak barrique barrels are most used for red wine ageing, yet there is a lack of studies investigating how slight modifications of medium toasting intensity influence aroma composition, particularly combined with different wood grain density. Further, since Cabernet Sauvignon red wine is one of the most widely cultivated and economically important red grape and wine variety in Croatia, it was used for this study.

Therefore, the aim of this study was to monitor the changes in the aroma profile of Cabernet Sauvignon red wine aged for 12 months in stainless steel tanks and oak barrels with different intensities of medium toasting (medium, medium plus, and medium long). Further, different grain density of the wood staves was included (Excellence and Premium), using the same intensity of medium toasting. Two consecutive wine vintages were observed to establish the different or similar trends in aroma profile changes. Special emphasis was placed on the determination of key aroma compounds and their changes during ageing of the wine, along with sensory evaluation of samples. It was expected that results would contribute to a better understanding of the intensity of medium toasting and its influence on the aroma profile of Cabernet Sauvignon wine.

## 2. Materials and Methods

### 2.1. Materials

#### 2.1.1. Chemical Reagents

For this study, chemical reagents from Sigma-Aldrich, St. Louis, MO, USA (myrtenol standard), and from Kemika, Zagreb, Croatia (sodium chloride), were used.

#### 2.1.2. Wooden Barrels

Wooden barrels used for this study were produced in Našice, Croatia, in the Auric Barrels cooperage. They used a 70:30 combination of sessile oak (*Quercus petraea* L.) and pedunculated oak *(Quercus robur* L.) to produce Excellence (3–5 grains/cm) and Premium (5–7 grains/cm) barrels. This study included Excellence and Premium barrels with medium toasting level (slowly rising temperature from 100 to 190 °C), and Excellence barrels with medium plus (rising temperature from 110 to 205 °C) and medium long (rising temperature from 110 to 210 °C) toasting. All medium toasting processes lasted 60 min in total, except medium long toasting, which lasted 65 min (5 more min at the highest temperature). Two sets of the mentioned barrels were purchased from Auric barrels for both wine vintages (Cabernet Sauvignon 2020 and 2021).

#### 2.1.3. Wine Samples

Cabernet Sauvignon grapes were cultivated and harvested in the Kutjevo vineyard. The harvest occurred on 27 October 2020 and 11 November 2021. The dates of harvest depended on grape ripeness, which is directly related to the climatic conditions in the vineyard. The average monthly values of air temperature, sunshine hours, and precipitation in the Kutjevo vineyard during 2020 and 2021 can be found in our previous study [8]. After harvest and crushing, a mash was obtained that had been macerated in stainless steel Vinimatic for 12 days, and punch-downs were carried out two times in a day. The *Saccharomyces cerevisiae* Siha Finesse Red yeast (BHF Technologies, Oakleigh, Australia) was used for fermentation, which was carried out in a stainless steel tank at 23–25 °C. The vinification process was fully completed in March 2021, and in May 2022, wines were divided into 5 different vessels: stainless steel tanks (S); Excellence oak barrels with medium (EM), medium plus (E+), and medium long (EL) toasting; and Premium oak barrels with medium (PM) toasting. Wines aged in all vessels for 12 months, and sampling was conducted every 3 months. First sampling of 2020 vintage Cabernet Sauvignon was conducted after 3 months of ageing in June 2021 (I), second after 6 months of ageing in September 2021 (II), third after 9 months of ageing in December 2021 (III), and fourth after 12 months of ageing in March 2022 (IV). First sampling of 2021 vintage Cabernet Sauvignon was conducted after 3 months of ageing in August 2022 (I), second after 6 months of ageing in November 2022 (II), third after 9 months of ageing in February 2023 (III), and fourth after 12 months of ageing in May 2023 (IV). Sampling and all analyses were conducted in triplicate. The identical procedure was applied for both wine vintages.

### 2.2. Methods

#### 2.2.1. Aroma Analysis

Aroma profile in wine samples was analysed on an Agilent 7890B gas chromatograph (GC) with Agilent 5977A mass spectrometer (MS, Agilent Technologies, Santa Clara, CA, USA). As a sampling method, solid-phase microextraction (SPME) was applied, where a fibre with polydimethylsiloxane/divinylbenzene sorbent (PDMS/DVB), 65/10 (Agilent Technologies), was used. The detailed descriptions of GC/MS conditions, identification, and quantification methods of aroma compounds were already described in our previous studies [2,8,31,32]. Briefly, in a 10 mL headspace vial, 5 mL of the wine sample was mixed with 1 g of NaCl. For quantification purposes, 5 μL of an internal standard solution (myrtenol, 1 mg/L) was added to each sample. Such prepared vials were incubated on a magnetic stirrer at 40 °C and 300 rpm, followed by adsorption on a SPME fibre coated with polydimethylsiloxane/divinylbenzene (PDMS, 65 μm, Supelco, Bellefonte, PA, USA) for 45 min. The fibre was then inserted into the GC injector port for desorption at 250 °C for 7 min in splitless mode. Separation was performed on an HP-5MS capillary column (30 m × 0.25 mm × 0.25 μm) using helium (purity 99.999%) as the carrier gas at a flow rate of 1.0 mL/min. The GC oven temperature was programmed as follows: 40 °C (held 10 min), ramp to 120 °C at 3 °C/min, and ramp to 250 °C at 10 °C/min. The MS source and quadrupole temperatures were set at 230 °C and 150 °C, respectively. Mass spectra were acquired in the range *m*/*z* under electron ionisation at 70 eV. The quantification of compounds included a semi-quantitative method, including the use of an internal standard. The quantification of the rest of the compounds was determined tentatively through an internal standard. Compound identification was based on comparison of retention times, mass spectra, and linear retention indices (LRI) calculated using C7–C30 n-alkanes (analysed under the same GC/MS conditions), with NIST (National Institute of Standards and Technology, Gaithersburg, MD, USA) and Wiley mass spectral libraries. All samples were analysed in triplicate and expressed as mean values.

#### 2.2.2. Sensory and Descriptive Analysis

A panel of certified sensory evaluators (3 men, 2 women; age range 30–50 years), with more than 5 years of experience in sensory evaluation, trained during the “Uncorking rural heritage” project according to the Norwegian Institute of Organoleptics (NOFIMA) procedure, tasted all samples. For evaluation, they used the OIV (International Organisation of Vine and Wine) 100-point test [8,33]. They also made a descriptive analysis of samples, ranking the intensity (0–10) of body, sweetness, astringency, and certain aromatic notes found in Cabernet Sauvignon wine, like black currant, black cherry, blackberry, cherry, dry plum, smoke, plum jam, bell pepper, coffee, chocolate, cedar wood, oak wood, graphite, leather, pepper and flowers.

#### 2.2.3. Statistical Evaluation of Experimental Data

All results are presented as mean value ± standard deviation (SD) of three replicates. For one-way ANOVA and Fisher’s LSD test, Statistica 13.1 (StatSoft, Tulsa, OK, USA) software was used. For hierarchical cluster analysis (HCA), principal component analysis (PCA), Kruskal–Wallis test, and heatmaps, OriginPro 2016 (OriginLab Corporation, Northampton, MA, USA) software was used.

## 3. Results

**Figure 1:**
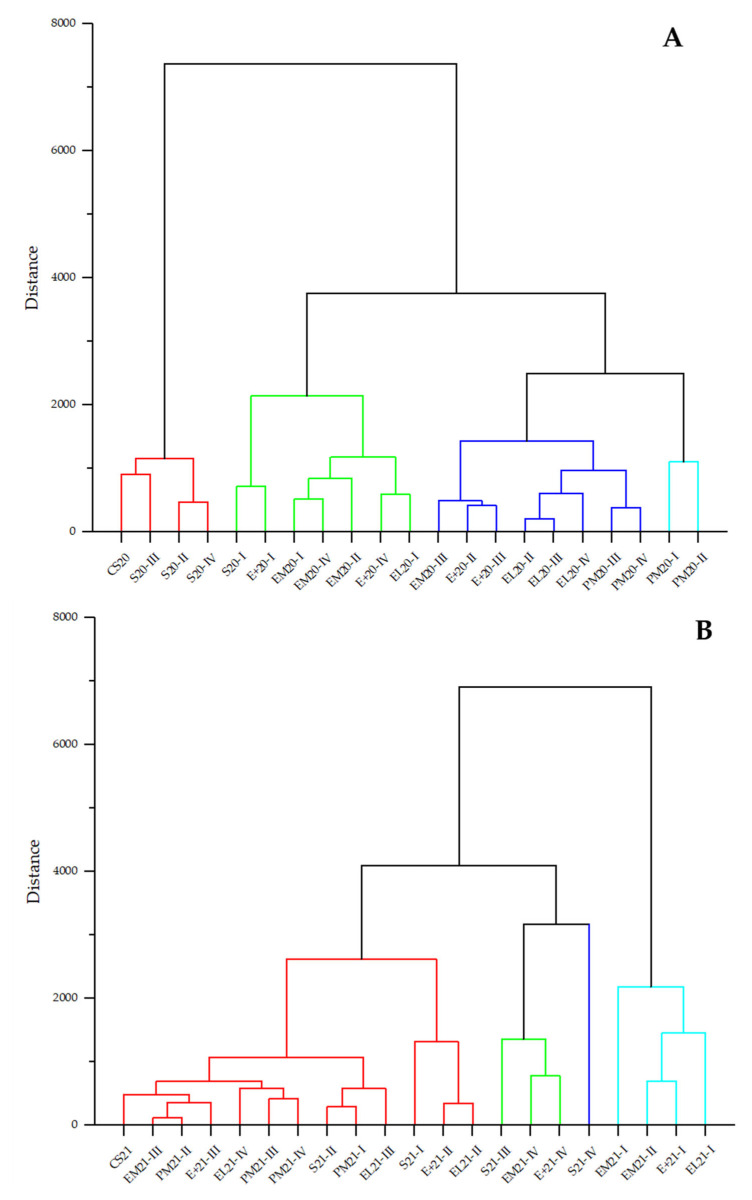
Hierarchical cluster analysis (HCA) of Cabernet Sauvignon wine and samples obtained during 12 months of ageing in different vessels. Abbreviations: CS20 and CS21—initial 2020 (**A**) and 2021 (**B**) vintage wines; S—stainless steel tank; EM—wooden barrel with excellent medium toasting; E+—wooden barrel with excellent medium plus toasting; EL—wooden barrel with excellent medium long toasting; PM—wooden barrel with premium medium toasting; I, II, III, IV—sampling after 3, 6, 9, and 12 months of ageing, respectively.

**Figure 2:**
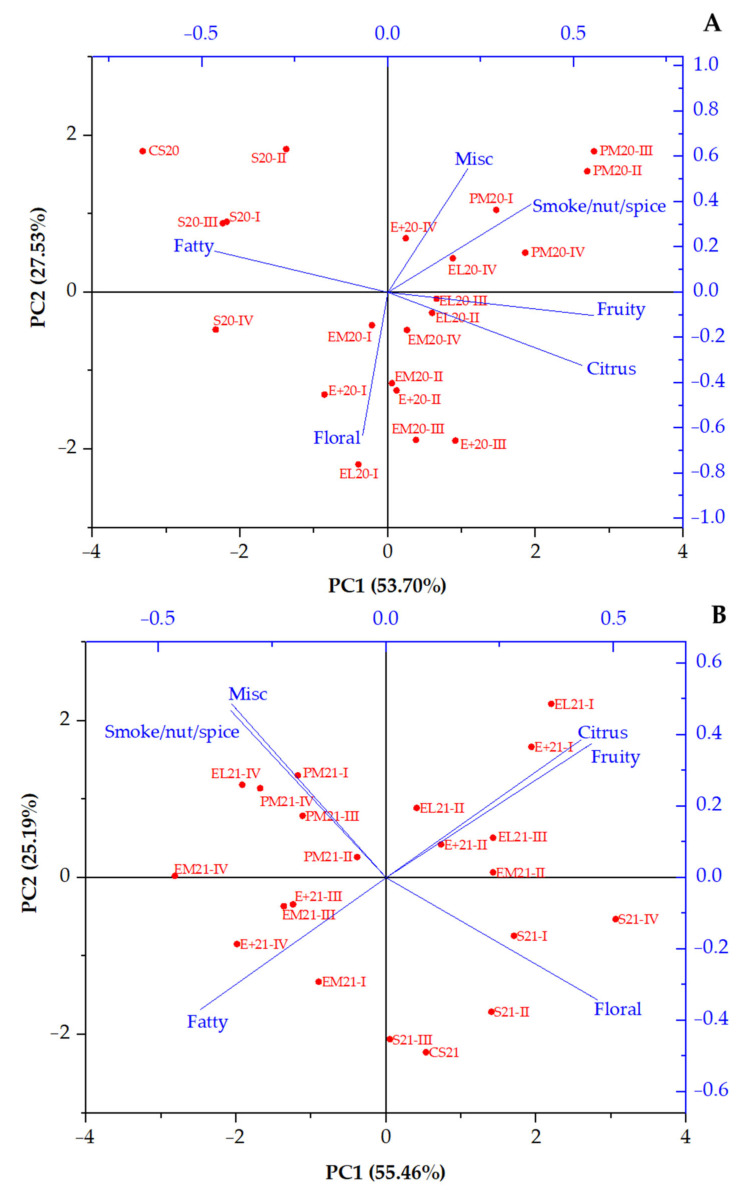
Principal component analysis (PCA) of Cabernet Sauvignon wine and samples obtained during 12 months of ageing in different vessels. Abbreviations: Misc.—miscellaneous (vinegar, caramel, and sulphurous); CS20 and CS21—initial 2020 (**A**) and 2021 (**B**) vintage wines; S—stainless steel tank; EM—wooden barrel with excellent medium toasting; E+—wooden barrel with excellent medium plus toasting; EL—wooden barrel with excellent medium long toasting; PM—wooden barrel with premium medium toasting; I, II, III, IV—sampling after 3, 6, 9, and 12 months of ageing, respectively.

**Figure 3:**
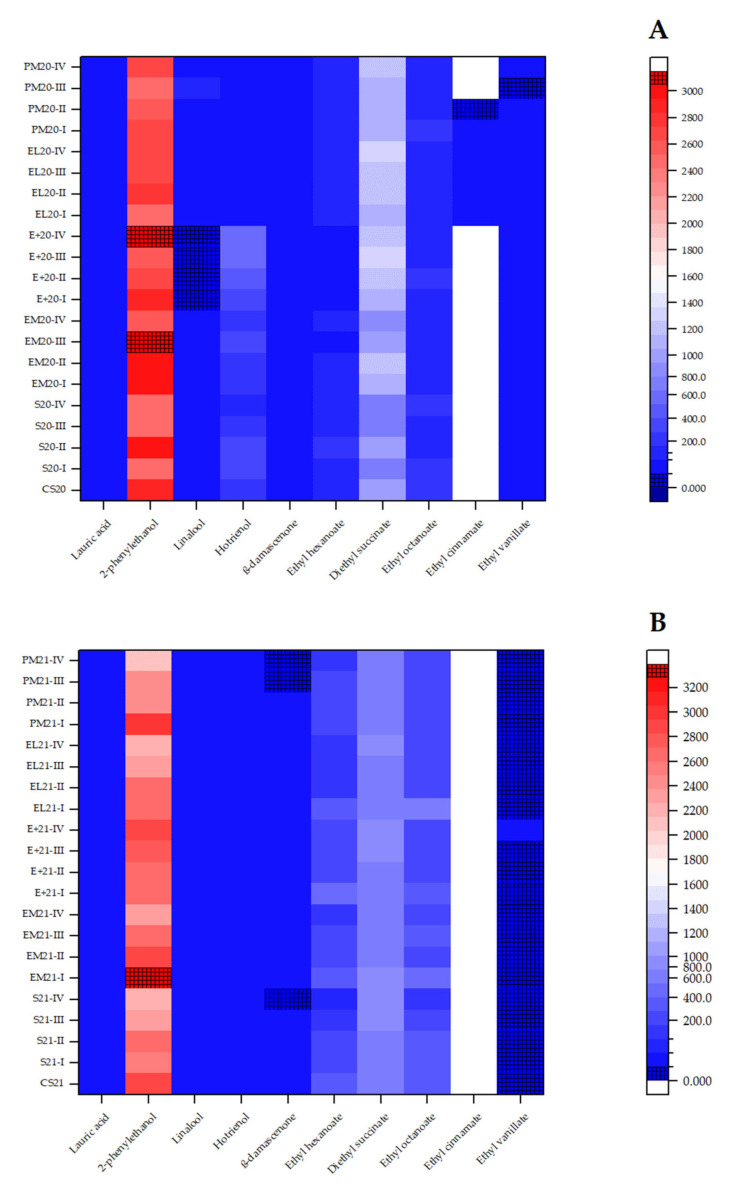
Heatmaps of the 10 key aroma compounds in Cabernet Sauvignon wine and samples obtained during 12 months of ageing in different vessels. Abbreviations: CS20 and CS21—initial 2020 (**A**) and 2021 (**B**) vintage wines; S—stainless steel tank; EM—wooden barrel with excellent medium toasting; E+—wooden barrel with excellent medium plus toasting; EL—wooden barrel with excellent medium long toasting; PM—wooden barrel with premium medium toasting; I, II, III, IV—sampling after 3, 6, 9, and 12 months of ageing, respectively.

**Figure 4:**
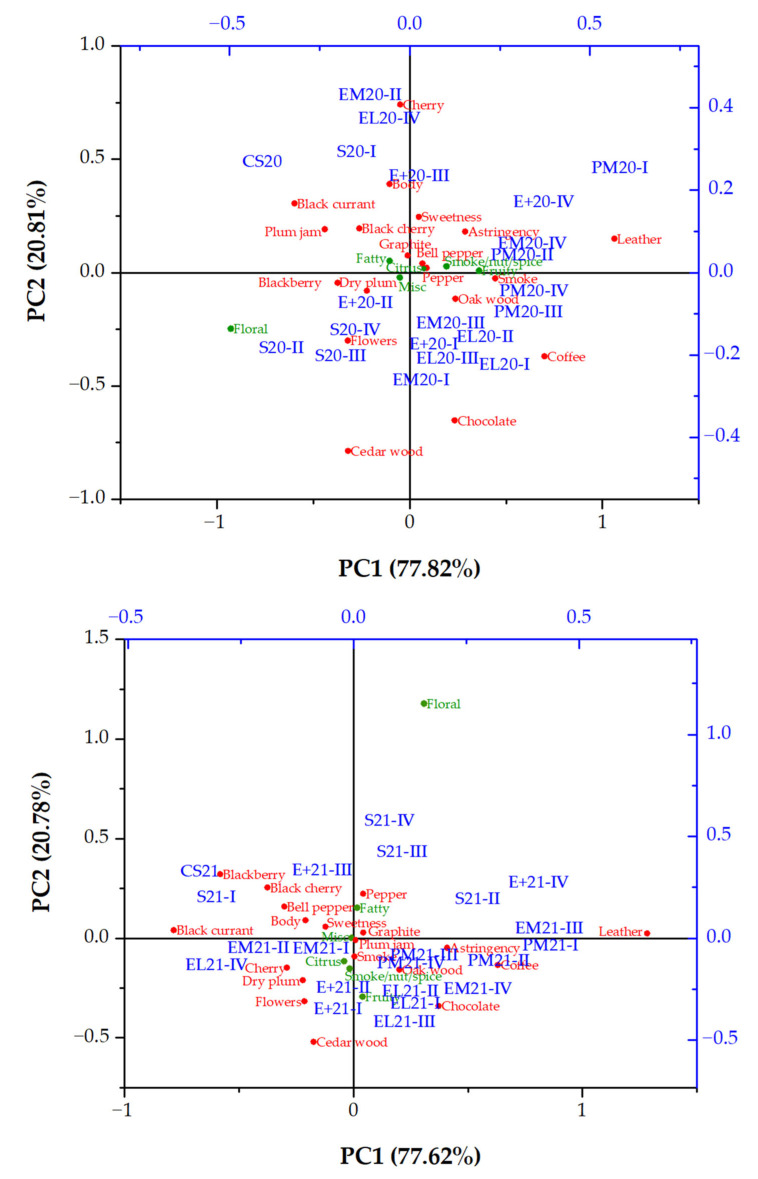
PCA biplot integrating GC/MS-based (green colour) aroma profile and descriptive analysis (red colour) of 2020 and 2021 vintage Cabernet Sauvignon wine and samples obtained during 12 months of ageing in different vessels. Abbreviations: CS20 and CS21—initial 2020 and 2021 vintage wines, respectively; S—stainless steel tank; EM—wooden barrel with excellent medium toasting; E+—wooden barrel with excellent medium plus toasting; EL—wooden barrel with excellent medium long toasting; PM—wooden barrel with premium medium toasting; I, II, III, IV—sampling after 3, 6, 9, and 12 months of ageing, respectively.

The results of aroma profile analyses of Cabernet Sauvignon wine, 2020 and 2021 vintage, and the samples obtained during 12 months of their ageing in S tank, EM, E+, EL, and PM barrels, are presented in Figure 1, Figure 2, Figure 3 and Figure 4, Table 1 and Table 2, and Tables S1–S8 in the Supplementary File. The samples are marked chronologically: after 3 months of ageing (I), after six months of ageing (II), after 9 months of ageing (III), and after 12 months of ageing (IV).

### 3.1. Individual Aroma Compounds

The retention time and index, mass-to-charge ratio, odour threshold according to the literature sources [34,35,36,37,38,39,40,41,42,43,44], and odour description for each aroma compound found in the samples are presented in Table S11 in the Supplementary File, while their concentrations are presented in Tables S1–S8. For better insight, according to the odour perception threshold (OTH), the odour activity value (OAV) of aroma compounds was calculated, where the compounds with OAV ≥ 1 were considered as key aroma compounds with the highest contribution to the wine aroma profile [44]. Ten key aroma compounds (OAV ≥ 1) were selected, and their concentrations in the analysed wine samples are presented in Table 1 and Table 2.

All 48 identified aroma compounds were divided into acids, alcohols, carbonyl compounds, terpenes, esters, phenols, and lactones. Since each compound has a dominant odour, they were also divided accordingly into six groups: fatty, fruity, floral, citrus, and miscellaneous (vinegar, caramel, and sulphurous), and smoke/nut/spice. These divisions were used for statistical analysis and the creation of hierarchical clusters and PCA biplots. Tables S1–S8 present the concentration of each compound in the samples.

The aroma profile changed during ageing in all vessels compared to the initial wines. For example, acetic acid (Tables S1 and S2) showed a marked increase after 12 months of ageing of the 2021 vintage Cabernet Sauvignon (CS21), especially in the PM barrel (almost 10 times higher concentration than the initial one, 126.0 μg/L). After an initial decrease, a trend of increasing concentration was observed during storage of the 2020 vintage wine (CS20), but the final concentration in EM and E+ barrels after 12 months was lower than the initial value of 663.6 μg/L. Hexanoic acid exhibited a general decrease across all samples of CS20, and after 12 months of ageing, it was not detected in any samples, except in the E+ barrel, where an increase was observed. On the other hand, in the 2021 vintage samples, an increase was recorded, especially in the E+ barrel, where the highest concentration was measured after 12 months of ageing (111.2 μg/L). Decanoic, lauric, and myristic acids showed a similar trend of decreasing concentration in both wine vintages after 12 months of storage, compared to the initial wines. In the 2020 vintage samples, myristic acid was not even detected in any sample except in the initial one. A similar decreasing trend was observed for palmitic acid during the ageing of both wines, except for the wine from the stainless steel tank, where the highest concentration was measured (21.6 μg/L).

Terpenes are also presented in Tables S1 and S2. It can be observed that the E+20-IV sample contained the highest concentration of hotrienol (more than twice the initial value of 273.5 μg/L), and the E+21-IV sample contained the highest concentrations of linalool, hotrienol, and β-damascenone. Linalool oxide was not detected in any CS21 sample, but only in CS20 samples. Among the 2020 vintage samples, linalool oxide and β-damascenone had the highest concentrations in EL20-IV, but linalool and eugenol had the highest values in PM20-IV. All wooden barrels were favourable for eugenol, which was not found in the initial wines nor the samples from stainless steel tanks.

Further, nine alcohols were identified in the analysed samples (Tables S3 and S4). The most abundant alcohols were isoamyl alcohol (7.8 mg/L in CS20 wine and 11.1 mg/L in CS21 wine) and 2-phenylethanol (2.9 and 2.8 mg/L, respectively). The rest of the alcohols had concentrations below 2 mg/L. The 2021 vintage samples contained all nine alcohols, but among the 2020 vintage samples, 1-heptanol was not found. Moreover, CS20 wine and samples from the stainless steel tank did not contain 2-ethyl-1-hexanol. This alcohol was detected in the 2020 vintage samples aged in wooden barrels, with the highest concentration measured after 3 months of ageing (up to 47 μg/L), followed by a decrease to below 10 μg/L in all barrels after 12 months (and complete absence in EM20-IV). All 2021 vintage samples contained 2-ethyl-1-hexanol, but a similar trend was observed: an initial increase in concentration after 3 months of storage, followed by a gradual decrease with longer ageing. Similar behaviour was observed for 1-octanol in the 2020 vintage samples and isoamyl alcohol in the 2021 vintage samples, but also for methionol in both wines from wooden barrels. While wooden barrels resulted in a decrease in methionol, stainless steel tanks were more favourable for this compound and resulted in an increased concentration during ageing of both wine vintages. On the other hand, the ageing process resulted in an increased concentration of 2,3-butanediol, 1-hexanol, and benzyl alcohol, especially in wooden barrels. The same trend was noted for isoamyl alcohol in the 2020 vintage samples from all wooden barrels. The highest concentrations of 2,3-butanediol and 1-hexanol were measured in the 2021 vintage samples from the PM barrel after 12 months of ageing, reaching 1684.3 μg/L and 937.0 μg/L, respectively, which is 2–3 times higher than the initial values. In the 2020 vintage samples, the maximum concentrations after 12 months of ageing were observed in the E+ barrel for 2,3-butanediol (1367.0 μg/L) and in the EL barrel for 1-hexanol (794.4 μg/L), representing approximately 6- and 4-fold increases relative to their initial levels. Comparing all used vessels, the E+ barrel proved to be most favourable in enhancing the concentrations of benzyl alcohol, 1-octanol, and 2-phenylethanol in both wine vintages after 12 months of ageing.

The carbonyl compounds shown in Tables S5 and S6 included one ketone (geranyl acetone) and three aldehydes (benzaldehyde, myristaldehyde, and hexyl cinnamaldehyde). Geranyl acetone was not found in the initial CS20 wine nor in any samples obtained after ageing, regardless of the vessel type. However, it was found in the 2021 vintage samples, with the highest concentration in the initial wine, 17.8 μg/L. After 12 months of ageing, the concentration of geranyl acetone was lower in all vessels compared to the initial value, except for the EL21-IV sample, where no significant difference was observed. Benzaldehyde was only detected in the EL and PM barrels with 2020 vintage wine and the PM barrel with 2021 vintage wine, where its concentration increased during ageing. The EL and PM barrels were also favourable for hexyl cinnamaldehyde in 2020 vintage samples. The highest concentration of this compound after 12 months was measured in PM20-IV, reaching 8.5 μg/L, which is almost double the initial value. For the 2021 vintage samples, all wooden barrels increased hexyl cinnamaldehyde, with the maximum concentration measured in the E+21-IV sample (12.6 μg/L, more than three times higher than the initial one). The same sample contained the highest concentration of myristaldehyde, 6.1 μg/L. In contrast, EL and PM barrels resulted in a loss of this compound in 2021 vintage samples, and a complete absence in 2020 vintage samples after 12 months of ageing.

In Tables S5 and S6, the concentrations of volatile phenols (4-ethyl phenol, 4EP; 4-ethyl guaiacol, 4EG) and lactones (γ-butyrolactone, cis-whiskey lactone, γ-heptalactone, trans-whiskey lactone, and γ-nonalactone) are also presented. In both wine vintages, none of these compounds were found in the initial wines. Furthermore, the samples aged in the stainless steel tank contained only γ-butyrolactone, with a maximum concentration of 13.5 μg/L measured in S21-IV. All other volatile phenols and lactones were exclusively found in samples aged in oak barrels, with concentrations varying according to the toasting method and barrel type. In 2021 vintage samples, 4EG was not found in any barrel, whereas in the 2020 vintage samples, it was present in E+, EL, and PM barrels. The concentrations of all identified compounds increased significantly during ageing. For the 2020 vintage samples, the PM and E+ barrels stood out, where most of the maximum concentrations were measured after 12 months of ageing. Among 2021 vintage samples, the PM barrel exhibited the highest levels of γ-butyrolactone, 4EP, and cis-whiskey lactone. Meanwhile, trans-whiskey lactone and γ-nonalactone had the highest concentrations in the EM barrel after 12 months of storage (62.9 and 12.2 μg/L, respectively, which were similar to the 2020 vintage samples from the same barrel).

Sixteen esters were identified in the analysed samples (Tables S7 and S8). Their concentrations varied depending on wine vintage and vessel type, although several consistent trends were observed. For example, isoamyl lactate, diethyl succinate, and most of the ethyl esters (ethyl vanillate, ethyl laurate, ethyl myristate, ethyl palmitate, ethyl linoleate, ethyl oleate, and ethyl stearate) exhibited an increasing trend during 12 months of ageing in both vintages, particularly in samples aged in oak barrels. In the 2020 vintage, the highest concentrations of the above-mentioned esters were found in the E+, EL, and PM barrels after 12 months of ageing, whereas in the 2021 vintage, ageing in the E+ barrel proved to be most favourable for their accumulation. However, the stainless steel tank resulted in a decreased concentration of most esters compared to the initial wine. Isoamyl lactate was not detected in the initial wines and samples aged in the stainless steel tank, but only in the ones aged in wooden barrels. Its concentration reached a maximum of 48.0 μg/L in E+21-IV and 177.6 μg/L in PM20-IV. In 2020 vintage samples, ethyl 4-hydroxybutanoate was found in samples aged in E+, EL, and PM barrels, but with concentrations equal to (E+ barrel) or lower than the initial one (20.4 μg/L). In contrast, this ester was found in all 2021 samples, although its concentration remained significantly lower than the initial one (69.2 μg/L), despite the increasing trend during the ageing period. Ethyl hexanoate, ethyl octanoate, phenethyl acetate, ethyl decanoate, and methyl dihydrojasmonate showed a decreasing trend, resulting in concentrations mostly below the initial value. Exceptions included the EL20-IV, where the highest concentration of phenethyl acetate (47.3 μg/L) was measured; PM20-IV, where the concentration of ethyl decanoate reached the initial value (30.6 μg/L); or E+21-IV, where it was the highest (53.7 μg/L), along with the highest concentration of methyl dihydrojasmonate (5.3 μg/L). Ethyl cinnamate was not detected in any 2021 vintage samples, but only in 2020 vintage samples aged in EL (up to 7.9 μg/L by the end of the ageing period) and in the PM barrel, where its concentration decreased from 8.2 to 2.7 μg/L in the first 6 months of ageing and was not detected later.

Furthermore, it should be mentioned that ethyl acetate, known as one of the key esters in wine, was not quantified in this study. Its odour threshold is reported to be 7500 μg/L [38], and sometimes the applied HS-SPME-GC/MS method is not optimised or sensitive enough for the detection of aroma-active compounds present in lower concentrations. Ethyl acetate also co-eluted with ethanol under the selected chromatographic conditions, preventing accurate quantification. On the other hand, if present in higher concentrations, ethyl acetate does not contribute to wine aroma, but rather results in acetone or glue-like aromatic notes [45], which is not the case in this study.

### 3.2. Statistical Analysis of Aroma Groups

Since a great number of aroma compounds were analysed, and their behaviour varied during the ageing of two vintages of Cabernet Sauvignon in different vessels, for better insight and statistical analysis, all compounds were divided into meaningful groups. First, they were divided according to the chemical classification (acids, alcohols, carbonyl compounds, terpenes, esters, volatile phenols, and lactones) for hierarchical cluster analysis, using Ward’s linkage method and Euclidean distance (Figure 1). Second, a division was made according to the aromatic notes (fatty, fruity, floral, citrus, miscellaneous (vinegar, caramel, and sulphurous), and smoke/nut/spice) for PCA (Figure 2). And third, according to the OAV, ten aroma compounds were selected with a value higher than 1 (Table 3) and analysed using a heatmap (Figure 3).

#### 3.2.1. Cluster Analysis of Aroma Groups

The hierarchical cluster analysis (HCA) of aroma groups identified in Cabernet Sauvignon wines from the 2020 and 2021 vintage, and samples aged in different vessels, is presented in Figure 1.

According to the presented HCA dendrogram, it is evident that the aroma profile of both wines changed during ageing in different vessels, which were the primary factor of sample arrangement. Several groups of samples could be distinctly separated based on vessel type and ageing time. For example, in the 2020 vintage, the initial wine was clustered (red) with samples aged in stainless steel tanks for six or more months, indicating minimal aroma differentiation during ageing in inert materials. The samples aged in EM barrels formed a separate group, but still belonged to the green cluster with S20-I, E+20-I, E+20-IV, and EL20-I. The remaining oak-aged samples were separated in the blue cluster, except PM20-I and PM20-II, which were clearly distinguished on the right side of the dendrogram in a cyan cluster. These patterns suggest that the vessel type and different medium-toasting methods were the major determinants of cluster formation for the 2020 vintage samples, while ageing time influenced the non-linear evolution of aroma profile of the analysed samples.

On the other hand, the HCA dendrogram of the 2021 vintage separates the samples differently. The main red cluster included 13 samples from different vessels and different ageing stages, indicating that the ageing process led to convergent aroma profiles of samples at some point. However, by the end of the ageing period, the influence of vessel type, especially different medium-toasting methods, resulted in significantly divergent aroma profiles with final-stage samples scattered across multiple clusters.

Comparing two vintages, obvious differences were present, but HCA indicated that vessel type resulted in the broadest separation, although ageing time can override vessel effects. Another similarity in both vintages was the samples aged in the E+ barrel, which did not cluster according to the ageing time and were scattered across clusters, indicating an unpredictable trajectory in aroma development, unlike other samples.

#### 3.2.2. Principal Component Analysis

As mentioned before, aroma compounds were divided according to their aromatic notes, and a PCA biplot was made for both vintages (Figure 2). For this purpose, within each category (fruity, floral, citrus, fatty, miscellaneous, and smoke/nut/spice), the sum of concentrations (μg/L) of compounds was calculated. This approach allows an overview of the contribution of each aromatic family to the overall volatile profile of the wines. The main aromatic note for each compound is presented in Table 1, and their concentrations are presented in the Supplementary File.

It can be observed that PCA explained around 81% of total variance for both 2020 and 2021 vintage (53.7 and 55.46% for PC1, 27.53 and 25.19% for PC2, respectively). Figure 2A shows the PCA biplot for the 2020 vintage. Aromatic notes were distributed across the biplot, pulling in the directions of certain clustered samples. Fatty notes strongly pulled to the upper left quadrant (negative PC1, positive PC2), where initial wine and samples aged in stainless steel tanks for 3, 6, and 9 months were placed. The sample S20-IV was placed in the lower left quadrant and not clustered with any others. The misc. and smoke/nut/spice notes were observed in the samples from the PM barrel, as well as in the final-stage samples taken from the E+ and EL barrels. Floral notes prevailed in the early stages of ageing in EM, E+, and EL barrels. The rest of the samples were clustered in the lower right quadrant, correlating with fruity and citrus notes. For the 2021 vintage (Figure 2B), the vectors of these aromatic notes pulled into the upper right quadrant, associating with the samples obtained in the early stages of ageing in EL and E+ barrels. Initial wine and S-tank samples were placed on the positive side of PC1 and the negative side of PC2, along with the floral aromatic vector. The quadrant on the negative sides of PC1 and PC2 contained fatty notes correlating with the final stages of ageing in E+ barrels, and two EM samples (I and III). The upper left quadrant contained the rest of the samples, including the ones aged in the PM barrel. The misc. and smoke/nut/spice aromatic notes prevailed in these samples.

In comparison, it was evident that for both wines, the samples aged in stainless steel tanks were separated from the others and were very close to the initial wine, based on their main aromatic notes. Similarly, samples from PM barrels were also separated, along with miscellaneous and smoke/nut/spice notes, in both wines. Despite the similarities, obvious differences were observed between the two vintages, suggesting that aroma profile changes were also a result of vintage-driven extraction kinetics and depended on the aromatic composition of the initial wine.

#### 3.2.3. Key Aroma Compounds

Since various aroma compounds differently affect the aroma profile of wine, the OAV was calculated to determine the ones with the greatest influence (OAV ≥ 1). Therefore, ten aroma compounds were selected: lauric acid, 2-phenylethanol, linalool, hotrienol, β-damascenone, ethyl hexanoate, diethyl succinate, ethyl octanoate, ethyl cinnamate, and ethyl vanillate (Table 3). Since their concentrations changed during the ageing period, the OAV also changes accordingly, and therefore, the OAV range was presented.

Among the mentioned compounds, β-damascenone exhibited the highest OAV, in the range of 30.3–206.3, confirming its strong contribution to fruity and floral complexity. However, medium-toasted barrels and ageing time did not favour its concentrations, since it was the highest in stainless steel tanks at the earlier stages of the ageing period of both wine vintages. Second-highest OAV was calculated for ethyl octanoate (16.7–104.4), another major driver of fruity characteristics, but its concentration and impact also decreased during ageing. Regardless of its high concentration, 2-phenylethanol had the OAV in the range of 2.1–3.4, and it follows the decreasing trend of fruity contributors. Linalool and hotrienol showed significant OAV (up to 9.6 and 5.2, respectively) in several samples, contributing to fresh floral and citrus notes. Hotrienol, however, had OAV lower than 1 in all samples of 2021 vintage wine due to its low concentrations. As expected, ageing in medium-toasted barrels resulted in enhanced vanilla and spicy notes, and therefore a notable OAV was calculated in most samples. The main difference was noted between wine vintages, because the 2021 vintage Cabernet Sauvignon did not contain ethyl cinnamate, and concentrations of ethyl vanillate were lower than those measured in 2020 vintage samples (the highest OAV of 7.0 was calculated for E+20-IV). Two more esters indicated a strong contribution to the wine aroma: ethyl hexanoate and diethyl succinate. While its OAV was lower (up to 1.1), diethyl succinate had higher concentrations than ethyl hexanoate (0.1–4.2) in all samples, following an increasing trend during ageing in wooden barrels, especially in E+ barrels. This was not the case for ethyl hexanoate, which exhibited a decreasing trend. Lauric acid had the OAV higher than 1 in all samples, contributing to the creamy and fatty nuances that decreased during the ageing of wine.

For better insight, a heatmap was made for both vintages and presented in Figure 3.

At first glance, both heatmaps are very similar, but there are some evident differences. In both vintages, 2-phenylethanol had the highest concentration, and it was indicated with red colour of different intensity across samples (the darker the red colour, the more abundant the compound). A slightly darker colour was noted in the 2020 vintage. Linalool had the lowest concentration in the E+ barrel with the 2020 vintage wine, while in the rest of the samples, no significant difference could be observed in blue colour on the heatmap. Linalool had the same colour intensity across all 2021 vintage samples, as it was noted for hotrienol in these samples, and β-damascenone in both wine vintages. Hotrienol was slightly more pronounced in the 2020 vintage, especially in the E+ barrel. Diethyl succinate was the second most abundant compound in all samples, indicated with a different intensity of light blue colour, and it was more abundant in the 2020 vintage wine. On the other hand, ethyl octanoate was more abundant in the 2021 vintage samples. These samples also had the lowest content of ethyl vanillate and had no ethyl cinnamate present, as mentioned. In the 2020 vintage, ethyl cinnamate was only detected in EL and PM barrels. Across both years, stainless steel tanks proved to have generally the lowest concentrations of aroma compounds, with very subtle variations among ageing stages. The influence of oak barrels with different medium-toasting methods seemed to depend on the initial composition of the wine. While more pronounced changes in terpenes appeared to be in 2020 vintage samples from oak barrels, the changes in ester content were more pronounced across 2021 vintage oak-aged samples. Between barrels, the heatmaps indicated that E+ and PM barrels had somewhat greater influence on the aroma profile of wines than the other two barrels.

#### 3.2.4. Effect Across Vessel Type

Across both wine vintages (Table S9), β-damascenone stood out with the highest OAVs in most of the aged samples, indicating its dominant role in contributing fruity/floral complexity, especially during ageing in stainless steel tanks, PM, and EL barrels. Ethyl octanoate and ethyl hexanoate consistently registered as the main ester contributors of fruity notes, especially ethyl octanoate, with OAVs higher than 16. Samples from stainless steel tank and from EL and PM barrels (especially the first months of ageing) showed generally elevated OAVs for the mentioned esters. Wood-derived markers, ethyl vanillate and ethyl cinnamate, were also clearly associated with the influence of barrel type and toasting method, since their OAVs were lower in stainless steel tanks and higher in oak barrels, especially EM and E+ barrels. While hotrienol showed dominant influence on floral and citrus nuances in the E+ barrel (especially vintage 2020), the PM barrel showed elevated OAVs for linalool, along with lauric acid. In general, ageing in oak barrels amplified slightly fruity, vanilla, and spicy dimensions that are somewhat muted in stainless steel tanks, where the aroma seemed to be less intense.

#### 3.2.5. Effect of Ageing Time 

Across most samples, total aromatic intensity increased or decreased progressively from 3 to 9 months of ageing, after which many of the compounds reached a plateau. Fruity esters, like ethyl octanoate and ethyl hexanoate, showed a consistent decrease in OAV in stainless steel tanks, followed by EM and E+ barrels, especially after 9 months of ageing. Except for EL20 samples and E+21 samples, the OAV of β-damascenone decreased, especially after 9 months of ageing. Ethyl vanillate showed an increasing trend in almost all samples, firstly in the E+ barrel. Interestingly, β-damascenone showed a higher impact on wine aroma in the first 3 to 6 months of ageing in several samples, while in the rest of the samples (S20, EL20, and E+21), the longer the ageing time, the higher its OAV and concentration. Lauric acid showed a consistent decreasing trend in its overall impact on all samples during ageing time. Terpenes retained their fruity and floral influence during ageing of most samples, slightly increasing over time in the E+ barrel. This barrel was the only one that favoured the 2-phenylethanol fruity influence during ageing time.

#### 3.2.6. Integration with PCA

These per-compound OAV patterns could explain the multivariate separation observed in PCA and heatmap clustering: the barrel-aged samples were separate from stainless steel tank samples by the first principal component, mainly driven by greater β-damascenone, ethyl octanoate, and ethyl vanillate impact. In contrast, samples from stainless steel tanks were associated with fatty descriptors (especially lauric acid) and lower total aromatic intensity. Ageing time had a low effect on samples from PM barrels, according to the PCA biplots, since they were all clustered close to each other. However, samples from Excellence barrels showed differences between the first and second half of the ageing period. Fruity compounds (like hotrienol, 2-phenylethanol, and even diethyl succinate) developed rapidly, within the first 6 or 9 months, whereas vanilla and spice markers accumulated steadily until 12 months. Nevertheless, wine vintage, precisely the composition of the initial wine, played a significant role in the behaviour of all compounds, and it should be considered when explaining the similarities and differences among samples.

#### 3.2.7. Sensory Evaluation

The sensory evaluators have rated all samples by the 100-point test and described the most prevalent aromatic notes present in them. Their results are presented in Table 4 and Figure 4.

According to the results from the 100-point test (Table 4), the highest score was obtained for the 2020 and 2021 Cabernet Sauvignon aged in the EL barrel for 9 months (94.3), but after 12 months, the score was lower. However, after the full ageing period, the samples from the PM barrel had the highest points (91.3 for PM20-IV and 94.0 for PM21-IV) among all samples. The lowest score was noted for samples from E+ barrels after 12 months of ageing (78.7 and 77.3 points for the 2020 and 2021 vintage, respectively). The rest of the samples obtained the same (EM barrel) or slightly higher (S tank and EL barrel) points than the initial wine. All samples have obtained the highest points for visual parameters (colour and clarity). The variations occurred in nose and taste, which is consistent with the changes in aroma profile obtained by GC/MS.

The descriptive analysis results were presented in the Supplementary File (Tables S9 and S10). The Kruskal–Wallis test showed that there were no significant differences among samples at the level of 0.05 in the points obtained from the descriptive analysis. Therefore, the sum concentrations of aromatic groups and the points obtained in the descriptive analysis have been calculated and expressed as a percentage in the total aroma, or total points, respectively, and PCA was performed (Figure 4) simply to compare the descriptive analysis and GC/MS obtained results. Similarities between descriptive analysis conducted by sensory evaluators and aromatic notes determined by GC/MS have been noted. Both results included fruity (especially berry fruits, cherry, and plum), floral (no specific flower), wooden (cedar, oak), and smoky notes. Most of the aromatic notes were clustered in the middle of the biplot, meaning that they were not specifically pronounced in any sample. However, the initial wines, along with samples from stainless steel tanks, were mostly separated from others and clustered with berry aromas, especially in the 2020 vintage. The samples from toasted barrels were scattered throughout the biplot, but the ones obtained after 9 or 12 months of ageing were mostly clustered with smoke, wood, coffee, and chocolate aroma, especially samples from PM barrels. Floral aroma was not pronounced in any sample.

## 4. Discussion

Wine production involves several stages, each of which influences the final product. One of the most critical stages is ageing, maturing, or storage of wine, which depends on the temperature in the cellar, ageing time, vessel type, wine composition, oxygen exposure, and other factors [8]. The most interesting ageing vessels for wine are wooden barrels due to their active interaction with the product during ageing, unlike stainless steel tanks, which are inert. Wooden barrels can be manufactured from different wood species, treated with different toasting methods, and have different volumes, grain density, etc. Consequently, many studies have aimed to determine the influence of wooden barrels with different characteristics on wine composition, especially the aroma profile [8,12,22,24,46,47,48,49,50,51]. Different wood types (chestnut, oak wood, ash wood, acacia, and cherry) contain various odour-active compounds that are extracted into wine or react with wine components during ageing [12]. Further, studies have shown that barrel size [52] and toasting level [8] had a significant impact on the kinetics of compound extraction and the concentrations of aroma-active molecules in wine.

This study highlighted the significant influence of different medium-toasting methods of oak barrels (Excellence and Premium) during 12 months of ageing on the aroma profile of Cabernet Sauvignon wine (vintage 2020 and 2021). A comparison with ageing in a stainless steel tank was also conducted. In total, 48 aroma compounds were identified in the analysed wines. All of them are characteristic of Cabernet Sauvignon wine [53]. For better insight, aroma compounds were divided according to their chemical structure (acids, alcohols, carbonyl compounds, terpenes, esters, volatile phenols, and lactones) and main odour (fatty, fruity, floral, citrus, miscellaneous (vinegar, caramel, and sulphurous), and smoke/nut/spice).

The observed differences between two wine vintages before ageing were a result of the climate conditions during the harvest year. For example, acetic acid was higher in the initial 2020 vintage Cabernet Sauvignon wine. However, its concentration increased significantly during storage of the 2021 vintage wine, and the final concentrations were higher than in the 2020 samples from the same oak barrel. Nevertheless, the concentrations of acetic acid did not exceed the spoilage limit of 0.9 g/L in any sample [54]. The change in acetic acid concentration during ageing could be a result of oxidation of ethanol due to acetic acid bacteria metabolism in aerobic conditions [55]. The amount of acetic acid bacteria that remained in wine depended on vintage-specific factors, like the health of grape berries and the must composition. The oxygen required for this reaction could be naturally present in wine, or it could permeate through wooden barrel staves—a process known as microoxygenation [56].

The presence of oxygen also contributed to the chemical reaction of other compounds and the metabolism of other microorganisms. For example, *Brettanomyces* yeasts need small amounts of oxygen for survival. They produce volatile phenols, like 4-ethylphenol and 4-ethylguaiacol. The presence of these compounds in concentrations higher than several hundred μg/L could result in an undesirable aroma with horse sweat, leather, stable, or medicinal notes. In contrast, in lower concentrations, like in the samples analysed in this study, they contribute to a smoky, pleasant aroma [8,57]. However, volatile phenols, along with lactones, could also be extracted from wood to wine during ageing in wooden barrels, and they are considered as indicators of wine oak ageing. Lactones are cyclic carboxylic esters formed through esterification of carbonyl and hydroxyl groups, mostly during maceration and fermentation. Butyrolactone can be synthesised from amino acids or organic acids [58,59]. Therefore, in the samples analysed in this study, γ-butyrolactone was found in all vessels, including the stainless steel tank. However, its concentration increased only during ageing in oak barrels, and the final content depended on the wine vintage, barrel type, and toasting method. The accumulation of other lactones (cis-whiskey lactone, γ-heptalactone, trans-whiskey lactone, and γ-nonalactone) was also observed during the ageing of Cabernet Sauvignon wine in all oak barrels. These compounds contribute to the coconut, woody, smoky, and spicy aromas [23]. As mentioned, differences in their concentrations have been noted among barrel type, toasting method, and wine vintage. It could be observed that the Premium barrel with medium toasting, followed by the Excellence barrel with medium plus toasting, stood out among other samples in terms of the increase in concentrations of lactones.

Toasting level plays a great role in the final wine aroma during ageing in wooden barrels. Burning the inner surface of a barrel alters the chemical composition of wood. For example, some macromolecules can break down, releasing new aroma compounds that can be extracted into wine [29]. However, the extraction of wood compounds also depends on the barrel size, the wine/wood contact surface, and the wine composition. In this study, all barrels were the same size, but the starting aroma profiles of two vintages of Cabernet Sauvignon wine were slightly different and resulted in various aroma changes in the observed vessels. Similar conclusions have been made in a previous study, where ageing of Cabernet Sauvignon wine of two consecutive vintages in barrels with different toasting levels and volumes was investigated [24].

Furthermore, the ageing process also influences the compounds that are already present in wine. Higher alcohols are usually the most abundant group, mostly due to high concentrations of isoamyl alcohol and 2-phenylethanol, as was obtained in this study. Their total concentration reached up to 20 mg/L in both wine vintages after 12 months of ageing. This was still below the threshold of negative impact of 400 mg/L, after which they do not contribute to the herbaceous and fruity aroma, but have a negative influence on wine quality [60]. The increase in their concentration during ageing could be a result of ester hydrolysis, and vice versa, the decrease was related to the esterification, along with the evaporation through wood pores [8,61].

Esters, however, were the predominant group of aroma compounds in the Cabernet Sauvignon wine analysed in this study. Since ethanol is the most abundant alcohol, most of the identified esters in wines were ethyl esters. Part of the wine esters were formed during alcoholic fermentation, but the other part was formed by esterification during wine ageing [62]. On the other hand, their concentration could decrease due to their hydrolysis and oxidation influenced by microoxygenation and temperature [8,63], but also due to bonding with other compounds, like polyphenols [64,65,66]. Toasting level, wine composition, and barrel type showed a significant influence on the kinetics of those reactions, since different behaviour of esters was noticed in the analysed samples.

Toasting of wooden barrels can result in thermal degradation of lignin into its derivatives, and one of them is benzaldehyde [27]. The extraction of benzaldehyde and its concentration depended significantly on the toasting method and barrel type: it was found only in 2020 vintage samples aged in EL and PM barrels, and in 2021 vintage samples aged in PM barrels. The concentrations of other carbonyl compounds differed between wine vintages, which was usually related to alcohol oxidation, or to reactions with polyphenols (resulting in the formation of condensed tannins and stabilised colour compounds) and with acids (leading to ester formation) [67,68].

The behaviour of terpenes in the analysed wine could also be explained similarly: the changes in concentrations occur due to interactions with other compounds in wine, oxidation, or extraction from wood. The results in this study showed that eugenol was not detected in any initial wine nor in samples aged in stainless steel tanks, but only in wooden barrels, where its concentration increased with time. This indicated that it was extracted from oak barrels, and the concentration depended on the toasting method and barrel type (the highest concentration was measured in both wines aged in PM barrels). This is consistent with previous studies [8,17,67].

Nevertheless, when observing individual aroma compounds, it could be noted that, despite vintage-specific differences, several similar patterns during ageing of Cabernet Sauvignon wine in different vessels were present. This was visible in the HCA dendrogram and PCA biplot, where the wood-derived compounds, contributing to woody, spicy, and smoky notes, prevailed in the samples from wooden barrels, especially PM and E+ barrels, and those samples tend to cluster together. On the other hand, the stainless steel tank mostly preserved the floral and fatty (organic acids) aromas, and usually clustered with the initial wines. According to the OAV calculation, only 10 volatile compounds stood out as the ones that had the greatest impact on the aroma profile of the analysed Cabernet Sauvignon wine. The heatmaps of their concentration indicated that both wine vintages had similar key aroma markers, with slight differences in their behaviour during ageing in different vessels. However, the persistence of fatty acids and lower levels of esters in the stainless steel tank referred to a slightly fresher, but less complex aroma profile of wines aged in it. The scattering of samples aged in the E+ barrel suggested that they exhibit high variability in aroma profile during ageing, and possibly greater sensitivity to microoxygenation or higher compound extraction rates.

However, to establish the consumers’ acceptance of wine, a sensory evaluator tasted the samples and rated them according to the 100-point test and descriptive analysis. Main parameters observed were visual, olfactory, and mouth-feel perception [69]. From the results, it can be observed that red wine does improve during ageing, but the ageing vessel and medium-toasting intensity had a significant effect on it. As the GC/MS showed changes in the wine aroma profile, similar changes were also observed by the evaluators. However, while several key aroma compounds showed differences in concentrations and OAVs, these variations were not always reflected in the sensory perception. This suggests that small differences in volatile concentration may remain below the sensory detection threshold or may interact synergistically with other compounds, masking their impact on overall aroma.

## 5. Conclusions

This study provided a comprehensive characterisation of the aroma-active compounds in Cabernet Sauvignon wines from two consecutive vintages (2020 and 2021) that aged in five different vessels: stainless steel tanks, Excellence barrels with medium (EM), medium plus (E+), and medium long (EL) toasting, and Premium barrels with medium toasting (PM). According to the GC/MS analysis, OAV-driven sensory relevance, and PCA-based multivariate analysis, novel insights into how vessel type, toast level, barrel grain density, and vintage influence wine aroma evolution were obtained.

In total, 48 aroma compounds were identified by GC/MS, but only 10 of them were selected as key compounds according to the OAV ≥ 1. Besides β-damascenone, which had the highest OAV, key compounds also included two terpenes (linalool and hotrienol), fruity esters (ethyl octanoate, ethyl hexanoate, and diethyl succinate), spicy esters (ethyl cinnamate and ethyl vanillate), 2-phenylethanol, and lauric acid. Their development in wines strongly depended on vessel type, toasting regime, and ageing time. Differences between wine vintages also played a significant role in aroma changes, but the following main conclusions could be extracted:

- Stainless steel tank samples maintained lower total OAVs, preserving fresher, lighter profiles with reduced wood-derived influence.
- Excellence barrels with medium toasting showed moderate ester expression, but a lower tendency to accumulate β-damascenone and linalool.
- Excellence barrels with medium plus toasting exhibited enhanced aromatic enrichment, with moderate impact of β-damascenone and elevated OAVs of ethyl vanillate, and even ethyl cinnamate.
- Excellence barrels with medium long toasting demonstrated distinctive shifts towards spicy and smoky notes, with strong extraction of wood-derived compounds, which also included volatile phenols and lactones.
- Premium barrels with medium toasting resulted in elevated contents of β-damascenone, fruity esters, ethyl vanillate, and volatile phenols and lactones, and this led to a more balanced smoky, woody, and spicy aroma profile.

These findings demonstrated that a subtle shift in barrel toasting conditions can lead to disproportionate changes in wine aroma composition. For example, Premium barrels with medium toasting resulted in increased smoky, woody, and spicy notes, compared to Excellence barrels with the same toasting. This indicates that barrel grain density also played a key role in aroma evolution. Additionally, ageing time also strongly modulated aroma development, since fruity esters and β-damascenone, for example, changed rapidly within 6–9 months, and wood-derived and spicy aroma compounds accumulated progressively and steadily.

Overall, these highlights provide a deeper understanding of how barrel type, medium toasting method, and ageing time affect Cabernet Sauvignon red wine aroma development. Since these results demonstrated that even minor adjustments in barrel toasting, grain selection, and ageing time significantly change final wine aroma, they could be used by producers as a practical guide during the winemaking process to optimise wine sensory quality and meet consumer preferences.

## Figures and Tables

**Table 1:** Key aroma compounds with odour activity value (OAV) ≥ 1 in 2020 vintage Cabernet Sauvignon and samples obtained during 12-month storage in different vessels.

Sample	Lauric Acid (μg/L)	2-Phenylethanol (mg/L)	Linalool (µg/L)	Hotrienol (µg/L)	Β-Damascenone (µg/L)	Ethyl Hexanoate (µg/L)	Diethyl Succinate (µg/L)	Ethyl Octanoate (µg/L)	Ethyl Cinnamate (mg/L)	Ethyl Vanillate (mg/L)
CS20	45.6 ± 1.2 ^k^	2.9 ± 0.1 ^c^	10.0 ± 0.4 ^gh^	273.5 ± 3.9 ^j^	5.2 ± 0.1 ^a^	117.6 ± 0.1 ^l^	1026.6 ± 21.8 ^d^	251.6 ± 5.0 ^k^	-	12.0 ± 0.5 ^gh^
S20-I	24.9 ± 1.5 ^i^	2.4 ± 0.1 ^a^	9.1 ± 0.1 ^f^	323.4 ± 1.9 ^l^	10.3 ± 0.1 ^h^	127.1 ± 2.0 ^m^	756.7 ± 22.0 ^b^	178.3 ± 2.9 ^h^	-	7.8 ± 0.1 ^c^
S20-II	16.9 ± 0.2 ^ef^	3.0 ± 0.1 ^cd^	6.7 ± 0.2 ^e^	359.7 ± 1.1 ^m^	16.0 ± 0.2 ^m^	186.7 ± 3.3 ^o^	1023.3 ± 8.2 ^d^	147.6 ± 1.7 ^g^	-	7.9 ± 0.2 ^c^
S20-III	13.2 ± 0.1 ^c^	2.4 ± 0.1 ^a^	6.1 ± 0.1 ^e^	166.6 ± 2.0 ^h^	13.5 ± 0.1 ^k^	113.1 ± 1.3 ^k^	718.4 ± 6.2 ^ab^	136.3 ± 3.0 ^ef^	-	17.2 ± 0.1 ^k^
S20-IV	19.5 ± 0.1 ^g^	2.4 ± 0.1 ^a^	8.7 ± 0.4 ^f^	126.1 ± 2.5 ^g^	14.2 ± 0.1 ^kl^	149.5 ± 0.8 ^n^	665.5 ± 2.8 ^a^	204.1 ± 2.8 ^i^	-	16.8 ± 0.3 ^k^
EM20-I	24.2 ± 0.5 ^i^	3.0 ± 0.1 ^cd^	12.6 ± 0.4 ^i^	201.2 ± 0.7 ^i^	12.2 ± 0.3 ^j^	71.1 ± 1.4 ^e^	1108.1 ± 34.6 ^ef^	145.6 ± 3.0 ^g^	-	12.7 ± 0.3 ^hi^
EM20-II	18.6 ± 0.3 ^g^	3.0 ± 0.1 ^cd^	10.6 ± 0.1 ^h^	291.1 ± 8.3 ^k^	8.9 ± 0.3 ^ef^	60.3 ± 0.7 ^d^	1203.5 ± 11.9 ^hi^	100.8 ± 0.2 ^b^	-	13.2 ± 0.1 ^i^
EM20-III	16.5 ± 0.5 ^e^	3.1 ± 0.1 ^d^	10.6 ± 0.1 ^h^	300.9 ± 5.0 ^k^	5.7 ± 0.1 ^a^	49.1 ± 0.7 ^c^	1027.2 ± 22.9 ^d^	99.9 ± 4.8 ^b^	-	16.7 ± 0.3 ^k^
EM20-IV	18.4 ± 0.4 ^fg^	2.6 ± 0.1 ^ab^	5.2 ± 0.2 ^d^	203.9 ± 9.2 ^i^	8.4 ± 0.1 ^de^	106.2 ± 0.2 ^j^	847.6 ± 3.0 ^c^	128.0 ± 6.1 ^cd^	-	7.5 ± 0.2 ^bc^
E+20-I	9.7 ± 0.5 ^a^	2.9 ± 0.1 ^c^	3.9 ± 0.3 ^bc^	330.8 ± 1.5 ^l^	7.5 ± 0.1 ^bc^	14.6 ± 0.5 ^b^	1068.5 ± 12.5 ^de^	186.6 ± 1.1 ^h^	-	15.3 ± 0.1 ^j^
E+20-II	9.2 ± 0.3 ^a^	2.7 ± 0.1 ^b^	3.5 ± 0.2 ^b^	479.3 ± 1.2 ^n^	7.4 ± 0.2 ^bc^	10.4 ± 0.2 ^ab^	1209.8 ± 29.9 ^hij^	186.1 ± 1.9 ^h^	-	11.3 ± 0.1 ^fg^
E+20-III	9.6 ± 0.1 ^a^	2.5 ± 0.1 ^ab^	4.3 ± 0.1 ^c^	510.9 ± 5.8 ^o^	8.7 ± 0.2 ^e^	9.2 ± 0.1 ^a^	1358.5 ± 7.6 ^k^	123.9 ± 1.7 ^c^	-	10.4 ± 0.1 ^e^
E+20-IV	11.3 ± 0.3 ^b^	3.1 ± 0.1 ^d^	2.7 ± 0.2 ^a^	573.8 ± 5.6 ^p^	6.8 ± 0.1 ^b^	10.2 ± 0.1 ^ab^	1250.7 ± 9.6 ^ij^	132.4 ± 3.3 ^de^	-	21.1 ± 0.3 ^l^
EL20-I	14.9 ± 0.3 ^d^	2.5 ± 0.1 ^ab^	9.8 ± 0.1 ^g^	25.7 ± 0.5 ^bc^	9.5 ± 0.5 ^fg^	95.2 ± 1.9 ^i^	1101.1 ± 18.9 ^ef^	83.6 ± 0.8 ^a^	6.3 ± 0.1 ^b^	8.8 ± 0.6 ^d^
EL20-II	15.7 ± 0.1 ^de^	2.8 ± 0.1 ^bc^	14.8 ± 0.1 ^k^	29.5 ± 1.2 ^cd^	9.7 ± 0.1 ^gh^	90.1 ± 1.5 ^h^	1177.5 ± 10.8 ^gh^	90.5 ± 1.2 ^a^	6.6 ± 0.1 ^c^	7.0 ± 0.2 ^b^
EL20-III	14.9 ± 0.2 ^d^	2.7 ± 0.1 ^b^	15.2 ± 0.2 ^k^	14.2 ± 0.1 ^a^	11.4 ± 0.4 ^i^	107.5 ± 0.7 ^j^	1173.3 ± 26.3 ^gh^	90.2 ± 0.4 ^a^	6.6 ± 0.1 ^bc^	7.6 ± 0.1 ^bc^
EL20-IV	16.1 ± 0.1 ^de^	2.7 ± 0.1 ^b^	13.6 ± 0.3 ^j^	18.0 ± 0.2 ^ab^	14.4 ± 0.2 ^l^	90.4 ± 1.4 ^h^	1263.6 ± 14.6 ^j^	91.3 ± 1.1 ^a^	7.9 ± 0.1 ^d^	10.9 ± 0.3 ^ef^
PM20-I	21.1 ± 0.4 ^h^	2.7 ± 0.1 ^b^	34.8 ± 0.1 ^m^	38.4 ± 0.1 ^de^	13.7 ± 0.3 ^kl^	96.3 ± 3.7 ^i^	1130.4 ± 25.4 ^fg^	217.3 ± 1.3 ^j^	8.2 ± 0.1 ^d^	9.2 ± 0.2 ^d^
PM20-II	22.4 ± 0.1 ^h^	2.5 ± 0.1 ^ab^	46.0 ± 0.2 ^n^	50.0 ± 0.5 ^f^	9.6 ± 0.1 ^fg^	75.9 ± 2.3 ^fg^	1103.4 ± 18.2 ^ef^	142.7 ± 0.2 ^fg^	2.7 ± 0.1 ^a^	9.1 ± 0.1 ^d^
PM20-III	18.8 ± 0.3 ^g^	2.4 ± 0.1 ^a^	57.7 ± 0.3 ^o^	42.3 ± 0.8 ^ef^	7.8 ± 0.3 ^cd^	80.2 ± 1.3 ^g^	1131.7 ± 2.7 ^fg^	132.8 ± 1.0 ^de^	-	4.6 ± 0.2 ^a^
PM20-IV	39.8 ± 0.6 ^j^	2.6 ± 0.1 ^ab^	19.9 ± 0.5 ^l^	31.2 ± 0.2 ^cd^	11.7 ± 0.7 ^ij^	73.1 ± 0.9 ^ef^	1249.6 ± 29.4 ^ij^	133.4 ± 2.5 ^de^	-	10.5 ± 0.6 ^ef^

“-” not detected. Abbreviations: CS20−2020 vintage Cabernet Sauvignon sample prior storage; 20—vintage year 2020; S—stainless steel tank; EM—wooden barrel with excellent medium toasting; E+—wooden barrel with excellent medium plus toasting; EL—wooden barrel with excellent medium long toasting; PM—wooden barrel with premium medium toasting; I, II, III, IV—sampling after 3, 6, 9, and 12 months of ageing, respectively. Different superscript letters (a–p) in the same column indicate statistical difference determined by ANOVA, followed by Fisher’s (LSD) test with *p* < 0.05.

**Table 2:** Key aroma compounds with odour activity value (OAV) ≥ 1 in 2021 vintage Cabernet Sauvignon and samples obtained during 12-month storage in different vessels.

Sample	Lauric Acid (μg/L)	2-Phenylethanol (mg/L)	Linalool (µg/L)	HOTRIENOL (µg/L)	Β-Damascenone (µg/L)	Ethyl Hexanoate (µg/L)	Diethyl Succinate (µg/L)	Ethyl Octanoate (µg/L)	Ethyl Cinnamate (mg/L)	Ethyl Vanillate (mg/L)
CS21	31.5 ± 0.3 ^n^	2.8 ± 0.1 ^de^	8.5 ± 0.1 ^cd^	8.4 ± 0.1 ^fgh^	5.5 ± 0.1 ^d^	304.7 ± 1.1 ^k^	579.5 ± 0.9 ^ab^	396.2 ± 0.5 ^m^	-	2.0 ± 0.1 ^fg^
S21-I	27.7 ± 0.2 ^m^	2.6 ± 0.1 ^cd^	16.0 ± 0.6 ^l^	10.2 ± 0.3 ^l^	20.6 ± 0.1 ^m^	299.9 ± 6.0 ^k^	557.4 ± 13.1 ^a^	378.1 ± 1.1 ^l^	-	1.5 ± 0.1 ^c^
S21-II	19.9 ± 0.9 ^ij^	2.7± 0.1 ^d^	10.3 ± 0.2 ^gh^	6.8 ± 0.1 ^c^	14.6 ± 0.4 ^l^	275.8 ± 7.0 ^j^	608.2 ± 4.6 ^cd^	377.9 ± 7.5 ^l^	-	1.4 ± 0.1 ^c^
S21-III	13.4 ± 0.6 ^d^	2.4 ± 0.1 ^bc^	10.6 ± 0.4 ^h^	7.1 ± 0.2 ^cd^	7.6 ± 0.1 ^f^	128.8 ± 2.0 ^bc^	761.6 ± 8.8 ^i^	158.9 ± 3.8 ^b^	-	1.6 ± 0.1 ^cd^
S21-IV	9.1 ± 0.1 ^a^	2.2 ± 0.1 ^ab^	8.1 ± 0.1 ^bc^	5.0 ± 0.4 ^a^	3.8 ± 0.1 ^b^	95.9 ± 0.1 ^a^	804.1 ± 1.7 ^k^	128.4 ± 0.2 ^a^	-	1.5 ± 0.1 ^cd^
EM21-I	22.0 ± 0.9 ^l^	3.4 ± 0.1 ^g^	9.2 ± 0.4 ^def^	8.2 ± 0.2 ^efg^	12.5 ± 0.2 ^j^	345.9 ± 4.5 ^l^	790.0 ± 5.2 ^jk^	466.6 ± 4.1 ^n^	-	2.5 ± 0.1 ^h^
EM21-II	21.8 ± 0.4 ^l^	3.0 ± 0.1 ^ef^	9.8 ± 0.5 ^fg^	8.0 ± 0.2 ^efg^	8.5 ± 0.1 ^gh^	174.4 ± 1.0 ^f^	715.3 ± 18.8 ^gh^	290.7 ± 4.2 ^hi^	-	3.0 ± 0.1 ^i^
EM21-III	13.0 ± 0.8 ^cd^	2.6 ± 0.1 ^cd^	8.6 ± 0.1 ^cde^	11.0 ± 0.3 ^l^	7.6 ± 0.3 ^f^	192.8 ± 2.9 ^g^	633.2 ± 6.4 ^ef^	309.1 ± 3.0 ^jk^	-	4.1 ± 0.1 ^j^
EM21-IV	11.7 ± 0.1 ^bc^	2.3 ± 0.1 ^b^	7.8 ± 0.1 ^bc^	11.8 ± 0.1 ^m^	5.5 ± 0.1 ^cd^	117.6 ± 2.5 ^b^	786.2 ± 2.7 ^ij^	232.2 ± 2.1 ^e^	-	4.8 ± 0.1 ^k^
E+21-I	15.2 ± 0.2 ^ef^	2.6 ± 0.1 ^cd^	8.2 ± 0.3 ^bc^	10.0 ± 0.4 ^l^	8.1 ± 0.1 ^fg^	417.5 ± 7.3 ^n^	573.2 ± 11.3 ^ab^	317.5 ± 3.4 ^k^	-	1.4 ± 0.1 ^c^
E+21-II	20.6 ± 0.3 ^j^	2.7 ± 0.1 ^d^	9.9 ± 0.1 ^fgh^	6.9 ± 0.1 ^c^	12.6 ± 0.7 ^j^	219.7 ± 0.5 ^i^	705.3 ± 10.4 ^gh^	279.3 ± 5.8 ^g^	-	2.1 ± 0.1 ^g^
E+21-III	17.2 ± 0.4 ^g^	2.8 ± 0.1 ^de^	11.5 ± 0.4 ^i^	9.7 ± 0.5 ^jl^	10.2 ± 0.1 ^i^	214.0 ± 3.0 ^hi^	775.9 ± 4.0 ^ij^	266.2 ± 1.9 ^f^	-	4.0 ± 0.1 ^j^
E+21-IV	19.3 ± 0.2 ^hi^	2.9 ± 0.1 ^e^	12.7 ± 0.4 ^j^	11.6 ± 0.1 ^lm^	12.4 ± 0.3 ^j^	201.9 ± 1.6 ^gh^	802.4 ± 1.3 ^k^	223.9 ± 1.9 ^e^	-	5.2 ± 0.1 ^l^
EL21-I	16.1 ± 0.1 ^fg^	2.6 ± 0.1 ^cd^	9.4 ± 0.2 ^ef^	9.3 ± 0.2 ^ij^	16.6 ± 0.3 ^l^	371.8 ± 0.7 ^m^	631.6 ± 4.3 ^ef^	521.8 ± 3.7 ^o^	-	1.8 ± 0.1 ^ef^
EL21-II	21.0 ± 0.1 ^jl^	2.6 ± 0.1 ^cd^	9.3 ± 0.1 ^def^	5.9 ± 0.2 ^b^	8.9 ± 0.6 ^h^	149.2 ± 1.6 ^e^	697.4 ± 8.4 ^g^	265.5 ± 0.8 ^f^	-	1.7 ± 0.1 ^de^
EL21-III	18.5 ± 0.1 ^h^	2.3 ± 0.1 ^b^	9.4 ± 0.3 ^f^	7.6 ± 0.1 ^de^	9.7 ± 0.1 ^i^	143.4 ± 0.6 ^de^	725.4 ± 2.3 ^h^	204.3 ± 3.2 ^d^	-	1.5 ± 0.1 ^c^
EL21-IV	19.3 ± 0.1 ^hi^	2.3 ± 0.1 ^b^	10.4 ± 0.1 ^gh^	8.6 ± 0.3 ^ghi^	6.7 ± 0.2 ^e^	136.1 ± 2.9 ^cd^	849.2 ± 0.3 ^l^	176.7 ± 1.1 ^c^	-	1.4 ± 0.1 ^c^
PM21-I	25.1 ± 0.3 ^l^	3.1 ± 0.1 ^f^	5.6 ± 0.1 ^a^	8.4 ± 0.1 ^fgh^	8.6 ± 0.2 ^gh^	264.4 ± 12.4 ^j^	612.6 ± 5.3 ^de^	283.3 ± 4.1 ^gh^	-	2.7 ± 0.1 ^h^
PM21-II	18.6 ± 0.6 ^h^	2.4 ± 0.1 ^bc^	6.2 ± 0.1 ^a^	5.8 ± 0.2 ^b^	5.2 ± 0.1 ^cd^	196.6 ± 5.3 ^g^	644.0 ± 3.7 ^f^	298.2 ± 5.6 ^ij^	-	1.8 ± 0.1 ^ef^
PM21-III	14.8 ± 0.3 ^e^	2.5 ± 0.1 ^c^	7.5 ± 0.4 ^b^	7.9 ± 0.1 ^ef^	4.8 ± 0.2 ^c^	166.1 ± 2.4 ^f^	646.2 ± 12.0 ^f^	233.6 ± 5.2 ^e^	-	1.1 ± 0.1 ^b^
PM21-IV	11.4 ± 0.1 ^b^	2.1 ± 0.1 ^a^	10.3 ± 0.2 ^gh^	9.0 ± 0.1 ^hi^	3.0 ± 0.1 ^a^	133.8 ± 0.7 ^cd^	693.9 ± 1.8 ^g^	229.6 ± 5.3 ^e^	-	0.7 ± 0.1 ^a^

Abbreviations: CS21—2021 vintage Cabernet Sauvignon sample prior storage; 21—vintage year 2021; S—stainless steel tank; EM—wooden barrel with excellent medium toasting; E+—wooden barrel with excellent medium plus toasting; EL—wooden barrel with excellent medium long toasting; PM−wooden barrel with premium medium toasting; I, II, III, IV—sampling after 3, 6, 9, and 12 months of ageing, respectively. Different superscript letters (a–n) in the same column indicate statistical difference determined by ANOVA, followed by Fisher’s (LSD) test with *p* < 0.05.

**Table 3:** Key aroma compounds (OAV ≥ 1) identified in 2020 and 2021 vintage Cabernet Sauvignon wines and samples aged for 12 months in different vessels.

Compound	Odour Threshold (µg/L)	OAV Range	Lowest Concentration (Sample) (µg/L)	Highest Concentration (Sample) (µg/L)	Main Aroma Contribution
Lauric acid	5	1.8–9.1	9.1 (S21-IV)	45.6 (CS20)	Creamy, fatty aroma balance
2-phenylethanol	1000	2.1–3.4	2131.7 (PM21-IV)	3385.7 (EM21-I)	Rose, floral sweetness
Linalool	6	0.4–9.6	2.7 (E+20-IV)	57.7 (PM20-III)	Fresh floral/citrus notes
Hotrienol	110	0.1–5.2	5.1 (S21-IV)	573.9 (E+20-IV)	Floral aroma, honey nuance
β-damascenone	0.1	30.3–206.3	3.3 (PM21-IV)	20.6 (S21-I)	Fruity/floral complexity
Ethyl hexanoate	100	0.1–4.2	9.2 (E+20-III)	417.5 (E+21-I)	Banana, stone fruit notes
Diethyl succinate	1200	0.5–1.1	557.4 (S21-I)	1358.5 (E+20-III)	Mild fruity background
Ethyl octanoate	5	16.7–104.4	83.6 (EL20-I)	521.8 (EL21-I)	Fresh tropical fruit
Ethyl cinnamate	1.1	2.4–7.5	2.7 (PM20-II)	8.22 (PM20-I)	Spicy-fruity enhancement
Ethyl vanillate	3	0.2–7.0	0.66 (PM21-IV)	5.20 (E+21-IV)	Sweet vanilla aroma

**Table 4:** Organoleptic evaluation of 2020 and 2021 vintage Cabernet Sauvignon and samples obtained during 12-month storage in different vessels using the 100-point test.

Sample	Visual	Nose	Taste	Harmony	Total	Sample	Visual	Nose	Taste	Harmony	Total
CS20	15.0	26.7	34.3	9.7	85.7	CS21	15.0	25.0	33.7	10.3	84.0
S20-I	15.0	26.0	35.7	10.0	86.7	S21-I	15.0	24.3	37.7	10.0	87.0
S20-II	15.0	27.7	35.7	10.0	88.3	S21-II	15.0	23.3	35.0	10.0	83.3
S20-III	15.0	29.0	36.3	10.0	90.3	S21-III	15.0	25.3	38.0	9.7	88.0
S20-IV	15.0	29.3	37.3	10.0	91.7	S21-IV	15.0	23.7	38.0	9.7	86.3
EM20-I	15.0	28.7	39.3	10.7	93.7	EM21-I	15.0	26.7	38.3	10.0	90.0
EM20-II	15.0	25.7	36.7	10.0	87.3	EM21-II	15.0	24.3	37.3	10.0	86.7
EM20-III	15.0	27.0	35.7	10.0	87.7	EM21-III	15.0	22.7	32.3	9.0	79.0
EM20-IV	15.0	25.3	35.3	10.0	85.7	EM21-IV	15.0	23.0	37.3	10.0	85.3
E+20-I	15.0	27.7	37.7	10.0	90.3	E+21-I	15.0	26.0	38.0	9.7	88.7
E+20-II	15.0	28.7	37.7	10.3	91.7	E+21-II	15.0	26.0	39.0	10.3	90.3
E+20-III	15.0	24.7	37.7	10.3	87.7	E+21-III	15.0	25.3	36.3	10.0	86.7
E+20-IV	15.0	20.0	34.3	9.3	78.7	E+21-IV	15.0	20.7	32.0	9.7	77.3
EL20-I	15.0	22.3	37.3	10.0	84.7	EL21-I	15.0	24.7	35.0	9.7	84.3
EL20-II	15.0	26.0	37.7	9.7	88.3	EL21-II	15.0	27.7	37.7	10.0	90.3
EL20-III	15.0	29.0	40.0	10.3	94.3	EL21-III	15.0	28.3	41.0	10.0	94.3
EL20-IV	15.0	27.3	37.3	10.3	90.0	EL21-IV	15.0	24.0	37.0	10.0	86.0
PM20-I	15.0	22.0	33.7	9.3	80.0	PM21-I	15.0	19.7	33.7	9.3	77.7
PM20-II	15.0	22.0	36.7	10.0	83.7	PM21-II	15.0	25.3	35.7	9.3	85.3
PM20-III	15.0	25.3	38.0	10.0	88.3	PM21-III	15.0	24.7	37.7	10.0	87.3
PM20-IV	15.0	26.7	39.7	10.0	91.3	PM21-IV	15.0	28.0	40.7	10.3	94.0

Points were expressed as the average value of different judges. Maximal points for visual (clarity and colour) were 15, for nose (aroma purity, intensity, and quality) were 30, for taste (purity, intensity, persistence, and quality) were 44, and for harmony were 11, making a total of 100 points. Abbreviations: CS20 and CS21—initial 2020 and 2021 vintage wines, respectively; S—stainless steel tank; EM—wooden barrel with excellent medium toasting; E+—wooden barrel with excellent medium plus toasting; EL—wooden barrel with excellent medium long toasting; PM—wooden barrel with premium medium toasting; I, II, III, IV—sampling after 3, 6, 9, and 12 months of ageing, respectively.

## Data Availability

The original contributions presented in this study are included in the article/Supplementary Materials. Further inquiries can be directed to the corresponding author.

## Supplementary Materials

The following supporting information can be downloaded at: https://www.mdpi.com/article/10.3390/foods14183178/s1, Table S1: Acid and terpene concentrations (µg/L) in the aromatic profile of 2020 vintage Cabernet Sauvignon and samples obtained during 12-month storage in different vessels; Table S2: Acid and terpene concentrations (µg/L) in the aromatic profile of 2021 vintage Cabernet Sauvignon and samples obtained during 12-month storage in different vessels; Table S3: Alcohol concentrations in the aromatic profile of 2020 vintage Cabernet Sauvignon and samples obtained during 12-month storage in different vessels; Table S4: Alcohol concentrations in the aromatic profile of 2021 vintage Cabernet Sauvignon and samples obtained during 12-month storage in different vessels; Table S5: Carbonyl compound, volatile phenol and lactone concentrations (µg/L) in the aromatic profile of 2020 vintage Cabernet Sauvignon and samples obtained during 12-month storage in different vessels; Table S6: Carbonyl compound, volatile phenol and lactone concentrations (µg/L) in the aromatic profile of 2021 vintage Cabernet Sauvignon and samples obtained during 12-month storage in different vessels; Table S7: Ester concentrations (µg/L) in the aromatic profile of 2020 vintage Cabernet Sauvignon and samples obtained during 12-month storage in different vessels; Table S8: Ester concentrations (µg/L) in the aromatic profile of 2021 vintage Cabernet Sauvignon and samples obtained during 12-month storage in different vessels; Table S9: The results of descriptive analysis (points from 0 to 10 for each property or aromatic note) obtained by trained panellists for 2020 vintage Cabernet Sauvignon and samples obtained during 12-month storage in different vessels; Table S10: The results of descriptive analysis (points from 0 to 10 for each property or aromatic note) obtained by trained panellists for 2021 vintage Cabernet Sauvignon and samples obtained during 12-month storage in different vessels; Table S11: Main information of aroma compounds identified in 2020 and 2021 vintage Cabernet Sauvignon and samples obtained during 12-month ageing in different vessels.

## References

[1] Robinson J., Harding J., Voulliamouz J. (2012). Wine Grapes—A Complete Guide to 1,368 Vine Varieties, Including Their Origins and Flavours.

[2] Ivić I., Kopjar M., Jukić V., Bošnjak M., Maglica M., Mesić J., Pichler A. (2021). Aroma Profile and Chemical Composition of Reverse Osmosis and Nanofiltration Concentrates of Red Wine Cabernet Sauvignon. Molecules.

[3] Belda I., Ruiz J., Esteban-Fernández A., Navascués E., Marquina D., Santos A., Moreno-Arribas M. (2017). Microbial Contribution to Wine Aroma and Its Intended Use for Wine Quality Improvement. Molecules.

[4] Gamero Lluna A. (2011). Study of the Production and Release of Aromas During Winemaking Carried Out by Different Saccharomyces Species and Hybrids. Ph.D. Thesis.

[5] Padilla B., Gil J.V., Manzanares P. (2016). Past and Future of Non-Saccharomyces Yeasts: From Spoilage Microorganisms to Biotechnological Tools for Improving Wine Aroma Complexity. Front. Microbiol..

[6] Moreno J., Peinado R. (2012). Enological Chemistry.

[7] Jackson R.S. (2008). Wine Science: Principles and Applications.

[8] Pichler A., Ivić I., Mesić J., Drenjančević M., Kujundžić T., Marković T., Kopjar M. (2023). Aroma Profile of Merlot Red Wine Stored in Stainless-Steel Tanks and Wooden Barrels with Different Toasting Methods. Foods.

[9] Dunlevy J.D., Kalua C.M., Keyzers R.A., Boss P.K., Roubelakis-Angelakis K.A. (2009). The production of flavour and aroma compounds in grape berries. Grapevine Molecular Physiology and Biotechnology.

[10] Zhu F., Du B., Li J., Morata A., Loira I. (2016). Aroma compounds in wine. Grape and Wine Biotechnology.

[11] Montalvo F.F., García-Alcaraz J.L., Cámara E.M., Jiménez-Macías E., Blanco-Fernández J. (2021). Environmental Impact of Wine Fermentation in Steel and Concrete Tanks. J. Clean. Prod..

[12] Ortega-Heras M., Gonzalez-Huerta C., Herrera P., Gonzalez-Sanjose M.L. (2004). Changes in Wine Volatile Compounds of Varietal Wines during Ageing in Wood Barrels. Anal. Chim. Acta.

[13] Tao Y., García J.F., Sun D.-W. (2014). Advances in Wine Aging Technologies for Enhancing Wine Quality and Accelerating Wine Aging Process. Crit. Rev. Food Sci. Nutr..

[14] de Castro M.C., Bortoletto A.M., Silvello G.C., Alcarde A.R. (2021). Maturation related phenolic compounds in cachaça aged in new oak barrels. J. Inst. Brew..

[15] Wang C., Wang C., Tang K., Rao Z., Chen J. (2022). Effects of Different Aging Methods on the Phenolic Compounds and Antioxidant Activity of Red Wine. Fermentation.

[16] Lu H., Cheng B., Lan Y., Duan C., He F. (2023). Modifications in Aroma Characteristics of ‘Merlot’ Dry Red Wines Aged in American, French and Slovakian Oak Barrels with Different Toasting Degrees. Food Sci. Hum. Wellness.

[17] Carpena M., Pereira A.G., Prieto M.A., Simal-Gandara J. (2020). Wine Aging Technology: Fundamental Role of Wood Barrels. Foods.

[18] Bosso A., Petrozziello M., Santini D., Motta S., Guaita M., Marulli C. (2008). Effect of Grain Type and Toasting Conditions of Barrels on the Concentration of the Volatile Substances Released by the Wood and on the Sensory Characteristics of Montepulciano d’Abruzzo. J. Food Sci..

[19] Chira K., Teissedre P.-L. (2013). Extraction of oak volatiles and ellagitannins compounds and sensory profile of wine aged with French winewoods subjected to different toasting methods: Behaviour during storage. Food Chem..

[20] Collins T.S., Miles J.L., Boulton R.B., Ebeler S.E. (2015). Targeted volatile composition of oak wood samples taken during toasting at a commercial cooperage. Tetrahedron.

[21] Chira K., Teissedre P.-L. (2015). Chemical and Sensory Evaluation of Wine Matured in Oak Barrel: Effect of Oak Species Involved and Toasting Process. Eur. Food Res. Technol..

[22] Alañón M.E., Schumacher R., Castro-Vázquez L., Díaz-Maroto I.J., Díaz-Maroto M.C., Pérez-Coello M.S. (2013). Enological potential of chestnut wood for aging Tempranillo wines part I: Volatile compounds and sensorial properties. Food Res. Int..

[23] Prida A., Chatonnet P. (2010). Impact of Oak-Derived Compounds on the Olfactory Perception of Barrel-Aged Wines. Am. J. Enol. Vitic..

[24] Rodríguez-Rodríguez P., Gómez-Plaza E. (2011). Effect of Volume and Toast Level of French Oak Barrels (*Quercus petraea* L.) on Cabernet Sauvignon Wine Characteristics. Am. J. Enol. Vitic..

[25] Pilet A., de Sousa R.B., Ricardo-da-Silva J.M., Catarino S. (2019). Barrel-to-Barrel Variation of Phenolic and Mineral Composition of Red Wine. BIO Web Conf..

[26] Gómez García-Carpintero E., Gómez Gallego M.A., Sánchez-Palomo E., González Viñas M.A. (2012). Impact of alternative technique to ageing using oak chips in alcoholic or in malolactic fermentation on volatile and sensory composition of red wines. Food Chem..

[27] Fernández de Simón B., Martínez J., Sanz M., Cadahía E., Esteruelas E., Muñoz A.M. (2014). Volatile compounds and sensorial characterisation of red wine aged in cherry, chestnut, false acacia, ash and oak wood barrels. Food Chem..

[28] Spillman P.J., Sefton M.A., Gawel R. (2008). The effect of oak wood source, location of seasoning and coopering on the composition of volatile compounds in oak-matured wines. Aust. J. Grape Wine Res..

[29] González-Centeno M.R., Chira K., Teissedre P.-L. (2016). Ellagitannin Content, Volatile Composition and Sensory Profile of Wines from Different Countries Matured in Oak Barrels Subjected to Different Toasting Methods. Food Chem..

[30] Spillman P.J., Pollnitz A.P., Liacopoulos D., Pardon K.H., Sefton M.A. (1998). Formation and Degradation of Furfuryl Alcohol, 5-Methylfurfuryl Alcohol, Vanillyl Alcohol, and Their Ethyl Ethers in Barrel-Aged Wines. J. Agric. Food Chem..

[31] Ivić I., Kopjar M., Obhođaš J., Vinković A., Pichler D., Mesić J., Pichler A. (2021). Concentration with Nanofiltration of Red Wine Cabernet Sauvignon Produced from Conventionally and Ecologically Grown Grapes: Effect on Volatile Compounds and Chemical Composition. Membranes.

[32] Ivić I., Kopjar M., Obhođaš J., Vinković A., Babić J., Mesić J., Pichler A. (2022). Influence of the Processing Parameters on the Aroma Profile and Chemical Composition of Conventional and Ecological Cabernet Sauvignon Red Wines during Concentration by Reverse Osmosis. Membranes.

[33] Ailer Š., Valšíková M., Jedlička J., Mankovecký J., Baroň M. (2020). Influence of Sugar and Ethanol Content and Color of Wines On the Sensory Evaluation: From Wine Competition “Nemčiňany Wine Days” in Slovak Republic (2013–2016). Erwerbs-Obstbau.

[34] Yu H., Xie T., Xie J., Ai L., Tian H. (2019). Characterization of key aroma compounds in Chinese rice wine using gas chromatography-mass spectrometry and gas chromatography-olfactometry. Food Chem..

[35] de Vries C.J., Mokwena L.M., Buica A., McKay M. (2016). Determination of Volatile Phenol in Cabernet Sauvignon Wines, Made from Smoke-affected Grapes, by using HS-SPME GC-MS. S. Afr. J. Enol. Vitic..

[36] Arcari S.G., Caliari V., Sganzerla M., Godoy H.T. (2017). Volatile composition of Merlot red wine and its contribution to the aroma: Optimization and validation of analytical method. Talanta.

[37] Belitz H.D., Grosch W., Schieberle P. (2009). Food Chemistry.

[38] Ouyang X., Yuan G., Ren J., Wang L., Wang M., Li Y., Zhang B., Zhu B. (2017). Aromatic compounds and organoleptic features of fermented wolfberry wine: Effects of maceration time. Int. J. Food Prop..

[39] Brown R.C., Sefton M.A., Taylor D.K., Elsey G.M. (2006). An odour detection threshold determination of all four possible stereoisomers of oak lactone in a white and a red wine. Aust. J. Grape Wine Res..

[40] Chatonnet P., Dubourdie D., Boidron J., Pons M. (1992). The origin of ethylphenols in wines. J. Sci. Food Agric..

[41] Cao W., Shu N., Wen J., Yang Y., Jin Y., Lu W. (2022). Characterization of the Key Aroma Volatile Compounds in Nine Different Grape Varieties Wine by Headspace Gas Chromatography–Ion Mobility Spectrometry (HS-GC-IMS), Odor Activity Values (OAV) and Sensory Analysis. Foods.

[42] Cordente A.G., Solomon M., Schulkin A., Leigh Francis I., Barker A., Borneman A.R., Curtin C.D. (2018). Novel wine yeast with ARO4 and TYR1 mutations that overproduce ‘floral’ aroma compounds 2-phenylethanol and 2-phenylethyl acetate. Appl. Microbiol. Biotechnol..

[43] Miller G.C., Barker D., Pilkington L.I., Deed R.C. (2023). Synthesis of a novel isotopically labelled standard for quantification of γ-nonalactone in New Zealand Pinot noir via SIDA-SPE-GC–MS. Anal. Bioanal. Chem..

[44] Manolache M., Anamaria C., Pop N., Emese G., Pop T.I. (2019). Odor Activity Value in Red Wines Aroma From Three Wine Regions in Romania. Agriculture.

[45] Cliff M.A., Pickering G.J. (2006). Determination of odour detection thresholds for acetic acid and ethyl acetate in ice wine. J. Wine Res..

[46] Hernández-Carapia M.Á., Verde-Calvo J.R., Escalona-Buendía H.B., Peña-Álvarez A. (2023). Effect of Maturation with American Oak Chips on the Volatile and Sensory Profile of a Cabernet Sauvignon Rosé Wine and Its Comparison with Commercial Wines. Beverages.

[47] del Alamo-Sanza M., Nevares I. (2018). Oak wine barrel as an active vessel: A critical review of past and current knowledge. Crit. Rev. Food Sci. Nutr..

[48] Dumitriu G.-D., Teodosiu C., Gabur I., Cotea V.V., Peinado R.A., López de Lerma N. (2019). Evaluation of Aroma Compounds in the Process of Wine Ageing with Oak Chips. Foods.

[49] Cerdán T.G., Goñi D.T., Azpilicueta C.A. (2004). Accumulation of volatile compounds during ageing of two red wines with different composition. J. Food Eng..

[50] Cerdán T.G., Rodríguez Mozaz S., Ancín Azpilicueta C. (2002). Volatile composition of aged wine in used barrels of French oak and of American oak. Food Res. Int..

[51] Chira K., González-Centeno M.R., Teissedre P.L. (2017). Wine Ageing in Oak Barrel: Effect of Toasting Process. Agric. Res. Technol. Open Access J..

[52] Fernández de Simón B., Cadahía E., del Álamo M., Nevares I. (2010). Effect of size, seasoning and toasting in the volatile compounds in toasted oak wood and in a red wine treated with them. Anal. Chim. Acta.

[53] Tang K., Xi Y.-R., Ma Y., Zhang H.-N., Xu Y. (2019). Chemical and Sensory Characterization of Cabernet Sauvignon Wines from the Chinese Loess Plateau Region. Molecules.

[54] Lin J., Massonnet M., Cantu D. (2019). The genetic basis of grape and wine aroma. Hortic. Res..

[55] Garde-Cerdán T., Ancín-Azpilicueta C. (2006). Review of quality factors on wine ageing in oak barrels. Trends Food Sci. Technol..

[56] du Toit W.J. (2006). The Effect of Oxygen on the Composition and Microbiology of Red Wine. Ph.D. Thesis.

[57] Rayne S., Eggers N.J. (2007). 4-Ethylphenol and 4-ethylguaiacol in wines: Estimating non-microbial sourced contributions and toxicological considerations. J. Environ. Sci. Health Part B.

[58] Bueno J.E., Peinado R., Moreno J., Medina M., Moyano L., Zea L. (2003). Selection of Volatile Aroma Compounds by Statistical and Enological Criteria for Analytical Differentiation of Musts and Wines of Two Grape Varieties. J. Food Sci..

[59] Călugăr A., Coldea T.E., Pop C.R., Pop T.I., Babeș A.C., Bunea C.I., Manolache M., Gal E. (2020). Evaluation of Volatile Compounds during Ageing with Oak Chips and Oak Barrel of Muscat Ottonel Wine. Processes.

[60] Yue T.-X., Chi M., Song C.-Z., Liu M.-Y., Meng J.-F., Zhang Z.-W., Li M.-H. (2014). Aroma characterization of Cabernet Sauvignon wine from the Plateau of Yunnan (China) with different altitudes using SPME-GC/MS. Int. J. Food Prop..

[61] Pérez-Prieto L.J., López-Roca J.M., Gómez-Plaza E. (2003). Differences in major volatile compounds of red wines according to storage length and storage conditions. J. Food Compos. Anal..

[62] Ribereau-Gayon P., Glories Y., Maujean A., Dubourdieu D. (2006). Handbook of Enology, Volume 2: The Chemistry of Wine Stabilization and Treatments.

[63] Alpeza I. (2008). Temelji kemijskog sastava vina. Glas. Zaštite Bilja.

[64] Lambropoulos I., Roussis I.G. (2007). Inhibition of the decrease of volatile esters and terpenes during storage of a white wine and a model wine medium by caffeic acid and gallic acid. Food Res. Int..

[65] Longo R., Blackman J.W., Torley P.J., Rogiers S.Y., Schmidtke L.M. (2016). Changes in Volatile Composition and Sensory Attributes of Wines During Alcohol Content Reduction. J. Sci. Food Agric..

[66] Jung D.M., De Ropp J.S., Ebeler S.E. (2000). Study of interactions between food phenolics and aromatic flavors using one- and two-dimensional 1H NMR spectroscopy. J. Agric. Food Chem..

[67] Tarko T., Duda-Chodak A., Sroka P., Siuta M. (2020). The Impact of Oxygen at Various Stages of Vinification on the Chemical Composition and the Antioxidant and Sensory Properties of White and Red Wines. Int. J. Food Sci..

[68] Zhang D., Wei Z., Han Y., Duan Y., Shi B., Ma W. (2023). A Review on Wine Flavour Profiles Altered by Bottle Aging. Molecules.

[69] Guld Z., Nyitrainé Sárdy D., Gere A., Rácz A. (2020). Comparison of sensory evaluation techniques for Hungarian wines. J. Chemom..

