# Supplementary material for: A Comparative Study of Cabernet Sauvignon Red Wine Aroma Profiles During Ageing in Medium-Toasted Oak Barrels
Source: Foods. 2025 Sep 12;14(18):3178. doi: 10.3390/foods14183178 (PMC12469085; doi:10.3390/foods14183178)
Supplement: Supplementary file 1 [file foods-14-03178-s001.zip › foods-3852838-supplementary.pdf]

foods

MDPI

*Supplementary file*

# A Comparative Study of Cabernet Sauvignon Red Wine Aroma Profiles During Ageing in Medium-Toasted Oak Barrels

**Anita Pichler [1], Ivana Ivić [1,*], Josip Mesić [2], Brankica Svitlica [3], Nela Nedić Tiban [1], Iva Ostrun [1], Tanja Marković [4] and Mirela Kopjar [1]**

[1] Faculty of Food Technology Osijek, Josip Juraj Strossmayer University, F. Kuhača 18, 31000 Osijek, Croatia; anita.pichler@ptfos.hr (A.P.); nela.nedic@ptfos.hr (N.N.T.); iostrun@ptfos.hr (I.O.); mirela.kopjar@ptfos.hr (M.K.)
[2] Faculty of Tourism and Rural Development, Vukovarska 17, 34000 Požega, Croatia; jmesic@ftrr.hr
[3] Faculty of Agrobiotechnical Sciences Osijek, Josip Juraj Strossmayer University, V. Preloga 1, 31000 Osijek, Croatia; bsvitlica@fazos.hr
[4] Institute of Public Health, Franje Krežme 1, 31000 Osijek, Croatia; tanja.markovic.zzjz@gmail.com
* Correspondence: iivic@ptfos.hr; Tel.: +385-3122-4355

**Abstract:** Ageing in oak barrels affects the tertiary aroma of red wine, yet further research on the impact of different conditions used for medium toasting of barrels could still be conduct-ed. In this study, using the GC/MS method, aroma profiles of two consecutive vintages of Cabernet Sauvignon wine and samples aged for 12 months in different vessels were determined. Besides the stainless steel tank, Excellence barrel with medium, medium plus and medium long toasting, and Premium barrel with medium toasting were used. A panel of trained sensory evaluators rated the samples according to the OIV 100-points test. The results showed that wooden barrels have a greater impact on wine aroma during ageing, compared to the stainless steel tank. This impact depended on the initial wine composition. Further, slight differences in grain density and in time and temperature during medium toasting resulted in different aroma profiles of aged wines. Premium barrel with medium toasting resulted in higher concentrations of smoky and woody notes, while Excellence barrel with medium plus toasting stood out with the most pronounced changes of aroma profile, compared to other Excellence barrels. The initial wine aroma, including fruity and fatty notes, was most preserved in the stainless steel tank.

**Keywords:** Cabernet Sauvignon; oak barrel; medium toasting; ageing; aroma profile

Academic Editor: Liping Du

Received: 18 August 2025
Revised: 8 September 2025
Accepted: 10 September 2025
Published: 12 September 2025

**Citation:** Pichler, A.; Ivić, I.; Mesić, J.; Svitlica, B.; Nedić Tiban, N.; Ostrun, I.; Marković, T.; Kopjar, M. A Comparative Study of Cabernet Sauvignon Red Wine Aroma Profiles During Ageing in Medium-Toasted Oak Barrels. *Foods* **2025**, *14*, 3178. https://doi.org/10.3390/foods14183178

**Copyright:** © 2025 by the authors. Licensee MDPI, Basel, Switzerland. This article is an open access article distributed under the terms and conditions of the Creative Commons Attribution (CC BY) license (https://creativecommons.org/licenses/by/4.0/).

The results obtained by gas chromatography/mass spectrometry on samples of Cabernet Sauvignon wine are presented in Tables S1–S11.

**Table S1.** Acid and terpene concentrations (μg/L) in the aromatic profile of 2020 vintage Cabernet Sauvignon and samples obtained during 12-month storage in different vessels.

| Sample | Acids | | | | | | Terpenes | | | | | |
|---|---|---|---|---|---|---|---|---|---|---|---|---|
| | Acetic acid | Hexanoic acid | Decanoic acid | Lauric acid | Myristic acid | Palmitic acid | Linalool oxide | Linalool | Hotrienol | β-citronellol | Eugenol | β-damascenone |
| CS20 | $663.6 \pm 5.8^{k}$ | $88.6 \pm 0.4^{e}$ | $252.8 \pm 0.5^{p}$ | $45.6 \pm 1.2^{k}$ | $42.3 \pm 0.2^{a}$ | $77.3 \pm 1.4^{o}$ | $17.0 \pm 0.2^{jk}$ | $10.0 \pm 0.4^{gh}$ | $273.5 \pm 3.9^{j}$ | $13.4 \pm 0.2^{j}$ | - | $5.2 \pm 0.1^{a}$ |
| S20-I | $264.7 \pm 4.1^{d}$ | $33.9 \pm 0.9^{c}$ | $188.2 \pm 0.4^{o}$ | $24.9 \pm 1.5^{i}$ | - | $10.4 \pm 0.1^{fg}$ | $15.0 \pm 0.3^{i}$ | $9.1 \pm 0.1^{f}$ | $323.4 \pm 1.9^{l}$ | $9.8 \pm 0.5^{h}$ | - | $10.3 \pm 0.1^{h}$ |
| S20-II | $607.0 \pm 3.9^{j}$ | $27.7 \pm 0.3^{b}$ | $131.0 \pm 0.1^{l}$ | $16.9 \pm 0.2^{ef}$ | - | $35.0 \pm 0.1^{n}$ | $14.9 \pm 0.1^{i}$ | $6.7 \pm 0.2^{e}$ | $359.7 \pm 1.1^{m}$ | $13.0 \pm 0.1^{j}$ | - | $16.0 \pm 0.2^{m}$ |
| S20-III | $650.0 \pm 4.3^{k}$ | - | $126.4 \pm 0.2^{k}$ | $13.2 \pm 0.1^{c}$ | - | $10.7 \pm 0.2^{gh}$ | $13.3 \pm 0.2^{ef}$ | $6.1 \pm 0.1^{e}$ | $166.6 \pm 2.0^{h}$ | $11.6 \pm 0.1^{i}$ | - | $13.5 \pm 0.1^{k}$ |
| S20-IV | $770.2 \pm 3.6^{m}$ | - | $113.3 \pm 0.5^{j}$ | $19.5 \pm 0.1^{g}$ | - | $13.5 \pm 0.2^{k}$ | $11.8 \pm 0.2^{d}$ | $8.7 \pm 0.4^{f}$ | $126.1 \pm 2.5^{g}$ | $17.0 \pm 0.2^{k}$ | - | $14.2 \pm 0.1^{kl}$ |
| EM20-I | $274.5 \pm 9.7^{d}$ | $19.7 \pm 0.2^{a}$ | $150.0 \pm 0.7^{n}$ | $24.2 \pm 0.5^{i}$ | - | $7.2 \pm 0.2^{e}$ | $13.0 \pm 0.1^{ef}$ | $12.6 \pm 0.4^{i}$ | $201.2 \pm 0.7^{i}$ | $7.8 \pm 0.1^{g}$ | $7.0 \pm 0.1^{ef}$ | $12.2 \pm 0.3^{j}$ |
| EM20-II | $383.5 \pm 4.3^{f}$ | $22.1 \pm 0.2^{a}$ | $86.6 \pm 1.9^{g}$ | $18.6 \pm 0.3^{g}$ | - | $10.2 \pm 0.4^{fg}$ | $14.1 \pm 0.5^{gh}$ | $10.6 \pm 0.1^{h}$ | $291.1 \pm 8.3^{k}$ | $3.9 \pm 0.1^{b}$ | $8.7 \pm 0.4^{i}$ | $8.9 \pm 0.3^{ef}$ |
| EM20-III | $415.7 \pm 1.6^{g}$ | $33.5 \pm 0.1^{c}$ | $87.3 \pm 0.6^{g}$ | $16.5 \pm 0.5^{e}$ | - | $9.4 \pm 0.1^{f}$ | $16.6 \pm 0.1^{j}$ | $10.6 \pm 0.1^{h}$ | $300.9 \pm 5.0^{k}$ | $2.7 \pm 0.1^{a}$ | $9.6 \pm 0.1^{j}$ | $5.7 \pm 0.1^{a}$ |
| EM20-IV | $450.7 \pm 25.7^{h}$ | - | $96.0 \pm 5.4^{h}$ | $18.4 \pm 0.4^{fg}$ | - | $4.9 \pm 0.2^{bc}$ | $13.1 \pm 0.4^{ef}$ | $5.2 \pm 0.2^{d}$ | $203.9 \pm 9.2^{i}$ | $2.8 \pm 0.1^{a}$ | $8.0 \pm 0.1^{gh}$ | $8.4 \pm 0.1^{de}$ |
| E+20-I | $147.1 \pm 1.3^{a}$ | $28.0 \pm 0.5^{b}$ | $132.7 \pm 0.5^{lm}$ | $9.7 \pm 0.5^{a}$ | - | $6.9 \pm 0.1^{e}$ | $7.7 \pm 0.1^{a}$ | $3.9 \pm 0.3^{bc}$ | $330.8 \pm 1.5^{l}$ | $3.9 \pm 0.2^{b}$ | $5.8 \pm 0.1^{bc}$ | $7.5 \pm 0.1^{bc}$ |
| E+20-II | $161.3 \pm 5.2^{ab}$ | $119.1 \pm 5.3^{f}$ | $72.1 \pm 0.7^{f}$ | $9.2 \pm 0.3^{a}$ | - | $6.4 \pm 0.1^{de}$ | $12.7 \pm 0.5^{e}$ | $3.5 \pm 0.2^{b}$ | $479.3 \pm 1.2^{n}$ | $5.9 \pm 0.3^{d}$ | $6.7 \pm 0.2^{de}$ | $7.4 \pm 0.2^{bc}$ |
| E+20-III | $173.2 \pm 2.5^{b}$ | $31.7 \pm 0.2^{bc}$ | $60.1 \pm 0.1^{e}$ | $9.6 \pm 0.1^{a}$ | - | $3.4 \pm 0.2^{a}$ | $18.6 \pm 0.1^{l}$ | $4.3 \pm 0.1^{c}$ | $510.9 \pm 5.8^{o}$ | $6.8 \pm 0.2^{ef}$ | $8.4 \pm 0.3^{hi}$ | $8.7 \pm 0.2^{e}$ |
| E+20-IV | $233.5 \pm 0.7^{c}$ | $58.3 \pm 0.3^{d}$ | $135.1 \pm 1.0^{m}$ | $11.3 \pm 0.3^{b}$ | - | $5.8 \pm 0.1^{cd}$ | $13.4 \pm 0.2^{fg}$ | $2.7 \pm 0.2^{a}$ | $573.8 \pm 5.6^{p}$ | $6.5 \pm 0.2^{e}$ | $10.8 \pm 0.4^{k}$ | $6.8 \pm 0.1^{b}$ |
| EL20-I | $321.2 \pm 2.1^{e}$ | - | $57.1 \pm 0.2^{e}$ | $14.9 \pm 0.3^{d}$ | - | $4.0 \pm 0.1^{ab}$ | $10.6 \pm 0.1^{c}$ | $9.8 \pm 0.1^{g}$ | $25.7 \pm 0.5^{bc}$ | $5.2 \pm 0.1^{c}$ | $2.5 \pm 0.1^{a}$ | $9.5 \pm 0.5^{fg}$ |
| EL20-II | $481.9 \pm 1.4^{i}$ | - | $44.0 \pm 0.8^{b}$ | $15.7 \pm 0.1^{de}$ | - | $3.6 \pm 0.1^{a}$ | $14.7 \pm 0.4^{hi}$ | $14.8 \pm 0.1^{k}$ | $29.5 \pm 1.2^{cd}$ | $4.1 \pm 0.1^{b}$ | $5.3 \pm 0.1^{b}$ | $9.7 \pm 0.1^{gh}$ |
| EL20-III | $600.6 \pm 6.4^{j}$ | - | $45.2 \pm 0.1^{bc}$ | $14.9 \pm 0.2^{d}$ | - | $16.8 \pm 0.5^{l}$ | $15.3 \pm 0.1^{i}$ | $15.2 \pm 0.2^{k}$ | $14.2 \pm 0.1^{a}$ | $4.0 \pm 0.1^{b}$ | $6.2 \pm 0.1^{cd}$ | $11.4 \pm 0.4^{i}$ |
| EL20-IV | $870.9 \pm 0.3^{o}$ | - | $50.3 \pm 0.2^{d}$ | $16.1 \pm 0.1^{de}$ | - | $12.4 \pm 0.2^{ij}$ | $17.4 \pm 0.2^{k}$ | $13.6 \pm 0.3^{j}$ | $18.0 \pm 0.2^{ab}$ | $4.3 \pm 0.1^{b}$ | $5.9 \pm 0.1^{bc}$ | $14.4 \pm 0.2^{l}$ |
| PM20-I | $722.6 \pm 0.4^{l}$ | - | $107.7 \pm 0.3^{i}$ | $21.1 \pm 0.4^{h}$ | - | $24.2 \pm 0.3^{m}$ | $9.0 \pm 0.1^{b}$ | $34.8 \pm 0.1^{m}$ | $38.4 \pm 0.1^{de}$ | $5.9 \pm 0.3^{d}$ | $7.5 \pm 0.3^{fg}$ | $13.7 \pm 0.3^{kl}$ |
| PM20-II | $899.1 \pm 14.1^{p}$ | - | $31.7 \pm 0.2^{a}$ | $22.4 \pm 0.1^{h}$ | - | $12.6 \pm 0.1^{jk}$ | $11.6 \pm 0.1^{d}$ | $46.0 \pm 0.2^{n}$ | $50.0 \pm 0.5^{f}$ | $5.0 \pm 0.1^{c}$ | $10.1 \pm 0.3^{j}$ | $9.6 \pm 0.1^{fg}$ |
| PM20-III | $865.9 \pm 5.2^{o}$ | - | $28.9 \pm 0.1^{a}$ | $18.8 \pm 0.3^{g}$ | - | $11.6 \pm 0.1^{hi}$ | $12.6 \pm 0.2^{e}$ | $57.7 \pm 0.3^{o}$ | $42.3 \pm 0.8^{ef}$ | $5.4 \pm 0.1^{cd}$ | $12.6 \pm 0.1^{l}$ | $7.8 \pm 0.3^{cd}$ |
| PM20-IV | $830.5 \pm 3.7^{n}$ | - | $48.8 \pm 1.3^{cd}$ | $39.8 \pm 0.6^{j}$ | - | $11.2 \pm 0.2^{gh}$ | $12.8 \pm 0.3^{ef}$ | $19.9 \pm 0.5^{l}$ | $31.2 \pm 0.2^{cd}$ | $7.2 \pm 0.1^{f}$ | $17.9 \pm 0.4^{m}$ | $11.7 \pm 0.7^{ij}$ |

“-“ not detected. Abbreviations: CS20−2020 vintage Cabernet Sauvignon sample prior storage; 20−vintage year 2020; S−stainless steel tank; EM−wooden barrel with excellent medium toasting; E+−wooden barrel with excellent medium plus toasting; EL−wooden barrel with excellent medium long toasting; PM−wooden barrel with premium medium toasting; I, II, III, IV−sampling after 3, 6, 9 and 12 months of ageing, respectively. Different superscript letters (a−p) in the same column indicate statistical difference determined by ANOVA, Fisher’s (LSD) test with $p<0.05$.

**Table S2.** Acid and terpene concentrations (μg/L) in the aromatic profile of 2021 vintage Cabernet Sauvignon and samples obtained during 12-month storage in different vessels.

| Sample | Acids | | | | | | Terpenes | | | | | |
|---|---|---|---|---|---|---|---|---|---|---|---|---|
| | Acetic acid | Hexanoic acid | Decanoic acid | Lauric acid | Myristic acid | Palmitic acid | Linalool oxide | Linalool | Hotrienol | β-citronellol | Eugenol | β-damascenone |
| CS21 | 126.0 ± 3.5$^{a}$ | 79.9 ± 0.4$^{l}$ | 328.0 ± 3.4$^{m}$ | 31.5 ± 0.3$^{n}$ | 9.4 ± 0.3$^{l}$ | 17.2 ± 0.1$^{f}$ | - | 8.5 ± 0.1$^{cd}$ | 8.4 ± 0.1$^{fgh}$ | 13.3 ± 0.4$^{m}$ | - | 5.5 ± 0.1$^{d}$ |
| S21-I | 552.7 ± 10.2$^{gh}$ | 34.8 ± 0.1$^{c}$ | 107.5 ± 3.1$^{d}$ | 27.7 ± 0.2$^{m}$ | 5.1 ± 0.1$^{gh}$ | 3.3 ± 0.1$^{a}$ | - | 16.0 ± 0.6$^{l}$ | 10.2 ± 0.3$^{l}$ | 7.5 ± 0.2$^{f}$ | - | 20.6 ± 0.1$^{m}$ |
| S21-II | 318.8 ± 2.9$^{d}$ | 69.6 ± 1.4$^{ij}$ | 156.1 ± 1.7$^{f}$ | 19.9 ± 0.9$^{ij}$ | 7.5 ± 0.1$^{l}$ | 15.4 ± 0.1$^{e}$ | - | 10.3 ± 0.2$^{gh}$ | 6.8 ± 0.1$^{c}$ | 10.0 ± 0.4$^{jl}$ | - | 14.6 ± 0.4$^{l}$ |
| S21-III | 365.7 ± 3.6$^{e}$ | 89.5 ± 0.9$^{n}$ | 201.2 ± 0.6$^{hi}$ | 13.4 ± 0.6$^{d}$ | 4.2 ± 0.2$^{de}$ | 19.4 ± 0.2$^{gh}$ | - | 10.6 ± 0.4$^{h}$ | 7.1 ± 0.2$^{cd}$ | 8.8 ± 0.2$^{hi}$ | - | 7.6 ± 0.1$^{f}$ |
| S21-IV | 770.4 ± 17.4$^{l}$ | 107.0 ± 1.0$^{o}$ | 231.5 ± 0.8$^{l}$ | 9.1 ± 0.1$^{a}$ | 3.6 ± 0.1$^{c}$ | 21.6 ± 0.2$^{i}$ | - | 8.1 ± 0.1$^{bc}$ | 5.0 ± 0.4$^{a}$ | 10.1 ± 0.2$^{l}$ | - | 3.8 ± 0.1$^{b}$ |
| EM21-I | 166.1 ± 3.1$^{b}$ | 45.6 ± 1.3$^{e}$ | 195.1 ± 3.9$^{h}$ | 22.0 ± 0.9$^{l}$ | 4.4 ± 0.1$^{ef}$ | 18.9 ± 0.2$^{gh}$ | - | 9.2 ± 0.4$^{def}$ | 8.2 ± 0.2$^{efg}$ | 8.4 ± 0.1$^{gh}$ | 1.8 ± 0.1$^{b}$ | 12.5 ± 0.2$^{j}$ |
| EM21-II | 283.6 ± 4.9$^{c}$ | 67.4 ± 0.4$^{i}$ | 180.1 ± 0.6$^{g}$ | 21.8 ± 0.4$^{l}$ | 3.5 ± 0.3$^{c}$ | 14.3 ± 0.1$^{de}$ | - | 9.8 ± 0.5$^{fg}$ | 8.0 ± 0.2$^{efg}$ | 9.4 ± 0.5$^{ij}$ | 2.2 ± 0.1$^{c}$ | 8.5 ± 0.1$^{gh}$ |
| EM21-III | 404.4 ± 0.7$^{f}$ | 67.0 ± 0.5$^{i}$ | 203.5 ± 0.4$^{ij}$ | 13.0 ± 0.8$^{cd}$ | 4.2 ± 0.1$^{de}$ | 13.6 ± 0.3$^{cd}$ | - | 8.6 ± 0.1$^{cde}$ | 11.0 ± 0.3$^{l}$ | 6.4 ± 0.2$^{e}$ | 2.5 ± 0.1$^{c}$ | 7.6 ± 0.3$^{f}$ |
| EM21-IV | 619.7 ± 5.7$^{j}$ | 76.7 ± 0.1$^{l}$ | 209.7 ± 0.8$^{j}$ | 11.7 ± 0.1$^{bc}$ | 4.8 ± 0.1$^{fg}$ | 12.8 ± 0.1$^{cd}$ | - | 7.8 ± 0.1$^{bc}$ | 11.8 ± 0.1$^{m}$ | 5.5 ± 0.3$^{cd}$ | 2.8 ± 0.1$^{c}$ | 5.5 ± 0.1$^{cd}$ |
| E+21-I | 540.9 ± 2.9$^{gh}$ | 5.9 ± 0.2$^{a}$ | 83.6 ± 0.2$^{b}$ | 15.2 ± 0.2$^{ef}$ | 2.5 ± 0.2$^{ab}$ | 23.9 ± 0.3$^{j}$ | - | 8.2 ± 0.3$^{bc}$ | 10.0 ± 0.4$^{l}$ | 4.4 ± 0.2$^{a}$ | 1.1 ± 0.1$^{d}$ | 8.1 ± 0.1$^{fg}$ |
| E+21-II | 554.9 ± 0.8$^{gh}$ | 59.1 ± 0.8$^{g}$ | 75.8 ± 3.7$^{a}$ | 20.6 ± 0.3$^{j}$ | 2.4 ± 0.1$^{ab}$ | 17.8 ± 0.1$^{fg}$ | - | 9.9 ± 0.1$^{fgh}$ | 6.9 ± 0.1$^{c}$ | 6.2 ± 0.1$^{e}$ | 1.4 ± 0.1$^{a}$ | 12.6 ± 0.7$^{j}$ |
| E+21-III | 564.1 ± 5.8$^{hi}$ | 84.6 ± 2.0$^{m}$ | 235.2 ± 2.4$^{l}$ | 17.2 ± 0.4$^{g}$ | 4.8 ± 0.1$^{g}$ | 14.2 ± 0.1$^{de}$ | - | 11.5 ± 0.4$^{i}$ | 9.7 ± 0.5$^{jl}$ | 7.8 ± 0.1$^{fg}$ | 2.4 ± 0.1$^{c}$ | 10.2 ± 0.1$^{i}$ |
| E+21-IV | 584.8 ± 3.1$^{i}$ | 111.2 ± 1.1$^{p}$ | 285.0 ± 2.3$^{l}$ | 19.3 ± 0.2$^{hi}$ | 5.5 ± 0.3$^{hi}$ | 12.2 ± 0.1$^{c}$ | - | 12.7 ± 0.4$^{j}$ | 11.6 ± 0.1$^{lm}$ | 9.0 ± 0.1$^{hi}$ | 2.9 ± 0.1$^{d}$ | 12.4 ± 0.3$^{j}$ |
| EL21-I | 730.1 ± 15.4$^{l}$ | 12.3 ± 0.2$^{b}$ | 90.5 ± 1.3$^{c}$ | 16.1 ± 0.1$^{fg}$ | 3.8 ± 0.1$^{cd}$ | 32.6 ± 2.3$^{l}$ | - | 9.4 ± 0.2$^{ef}$ | 9.3 ± 0.2$^{ij}$ | 6.0 ± 0.1$^{de}$ | 1.6 ± 0.2$^{b}$ | 16.6 ± 0.3$^{l}$ |
| EL21-II | 795.9 ± 4.3$^{l}$ | 64.9 ± 0.1$^{h}$ | 158.0 ± 2.2$^{f}$ | 21.0 ± 0.1$^{jl}$ | 2.7 ± 0.1$^{b}$ | 19.7 ± 0.6$^{h}$ | - | 9.3 ± 0.1$^{def}$ | 5.9 ± 0.2$^{b}$ | 8.9 ± 0.1$^{hi}$ | 1.7 ± 0.1$^{b}$ | 8.9 ± 0.6$^{h}$ |
| EL21-III | 873.3 ± 7.0$^{m}$ | 47.3 ± 0.2$^{ef}$ | 158.7 ± 2.7$^{f}$ | 18.5 ± 0.1$^{h}$ | 2.8 ± 0.2$^{b}$ | 17.9 ± 0.3$^{fg}$ | - | 9.4 ± 0.3$^{f}$ | 7.6 ± 0.1$^{de}$ | 9.4 ± 0.1$^{i}$ | 1.7 ± 0.1$^{b}$ | 9.7 ± 0.1$^{i}$ |
| EL21-IV | 921.8 ± 2.1$^{n}$ | 70.0 ± 0.5$^{j}$ | 202.9 ± 3.3$^{i}$ | 19.3 ± 0.1$^{hi}$ | 3.5 ± 0.1$^{c}$ | 12.5 ± 0.4$^{c}$ | - | 10.4 ± 0.1$^{gh}$ | 8.6 ± 0.3$^{ghi}$ | 11.7 ± 0.1$^{l}$ | 1.7 ± 0.1$^{b}$ | 6.7 ± 0.2$^{e}$ |
| PM21-I | 348.4 ± 16.5$^{e}$ | 87.8 ± 2.0$^{n}$ | 95.7 ± 0.8$^{c}$ | 25.1 ± 0.3$^{l}$ | 5.8 ± 0.1$^{i}$ | 21.6 ± 0.4$^{i}$ | - | 5.6 ± 0.1$^{a}$ | 8.4 ± 0.1$^{fgh}$ | 5.4 ± 0.1$^{cd}$ | 3.6 ± 0.1$^{e}$ | 8.6 ± 0.2$^{gh}$ |
| PM21-II | 531.7 ± 5.5$^{g}$ | 65.5 ± 0.8$^{hi}$ | 107.9 ± 1.3$^{d}$ | 18.6 ± 0.6$^{h}$ | 6.7 ± 0.1$^{j}$ | 13.2 ± 0.2$^{cd}$ | - | 6.2 ± 0.1$^{a}$ | 5.8 ± 0.2$^{b}$ | 9.0 ± 0.2$^{hi}$ | 5.0 ± 0.1$^{f}$ | 5.2 ± 0.1$^{cd}$ |
| PM21-III | 1018.6 ± 5.1$^{o}$ | 49.9 ± 0.5$^{f}$ | 140.6 ± 1.6$^{e}$ | 14.8 ± 0.3$^{e}$ | 2.8 ± 0.2$^{b}$ | 8.1 ± 0.4$^{b}$ | - | 7.5 ± 0.4$^{b}$ | 7.9 ± 0.1$^{ef}$ | 4.8 ± 0.1$^{ab}$ | 4.9 ± 0.1$^{f}$ | 4.8 ± 0.2$^{c}$ |
| PM21-IV | 1151.9 ± 22.6$^{p}$ | 38.9 ± 0.8$^{d}$ | 185.0 ± 0.2$^{g}$ | 11.4 ± 0.1$^{b}$ | 2.1 ± 0.1$^{a}$ | 3.7 ± 0.1$^{a}$ | - | 10.3 ± 0.2$^{gh}$ | 9.0 ± 0.1$^{hi}$ | 5.2 ± 0.1$^{bc}$ | 5.7 ± 0.2$^{g}$ | 3.0 ± 0.1$^{a}$ |

"-" not detected. Abbreviations: CS21−2021 vintage Cabernet Sauvignon sample prior storage; 21−vintage year 2021; S−stainless steel tank; EM−wooden barrel with excellent medium toasting; E+−wooden barrel with excellent medium plus toasting; EL−wooden barrel with excellent medium long toasting; PM−wooden barrel with premium medium toasting; I, II, III, IV−sampling after 3, 6, 9 and 12 months of ageing, respectively. Different superscript letters (a−p) in the same column indicate statistical difference determined by ANOVA, Fisher's (LSD) test with $p$<0.05.

**Table S3.** Alcohol concentrations in the aromatic profile of 2020 vintage Cabernet Sauvignon and samples obtained during 12-month storage in different vessels.

| Sample | Isoamyl alcohol (mg/L) | 2,3-butanediol (µg/L) | 1-hexanol (µg/L) | 1-heptanol (µg/L) | Methionol (µg/L) | 2-ethyl-1-hexanol (µg/L) | Benzyl alcohol (µg/L) | 1-octanol (µg/L) | 2-phenylethanol (mg/L) |
|---|---|---|---|---|---|---|---|---|---|
| CS20 | 7.8 ± 0.1 $^{a}$ | 231.2 ± 5.1 $^{b}$ | 171.0 ± 1.0 $^{a}$ | - | 37.9 ± 0.2 $^{h}$ | - | 65.2 ± 0.5 $^{jk}$ | 35.5 ± 1.0 $^{h}$ | 2.9 ± 0.1 $^{c}$ |
| S20-I | 12.2 ± 0.2 $^{c}$ | 107.1 ± 4.1 $^{a}$ | 387.1 ± 11.9 $^{b}$ | - | 44.5 ± 1.6 $^{i}$ | - | 47.4 ± 0.1 $^{cd}$ | 56.6 ± 1.3 $^{j}$ | 2.4 ± 0.1 $^{a}$ |
| S20-II | 7.9 ± 0.1 $^{a}$ | 669.0 ± 4.3 $^{h}$ | 751.5 ± 25.0 $^{k}$ | - | 44.0 ± 0.2 $^{i}$ | - | 92.5 ± 0.4 $^{n}$ | 35.4 ± 0.6 $^{g}$ | 3.0 ± 0.1 $^{cd}$ |
| S20-III | 7.9 ± 0.1 $^{a}$ | 491.9 ± 7.0 $^{e}$ | 502.8 ± 9.8 $^{d}$ | - | 100.5 ± 2.0 $^{k}$ | - | 54.0 ± 0.3 $^{fg}$ | 32.3 ± 0.3 $^{e}$ | 2.4 ± 0.1 $^{a}$ |
| S20-IV | 7.9 ± 0.1 $^{a}$ | 1061.8 ± 7.7 $^{k}$ | 690.8 ± 4.3 $^{j}$ | - | 144.8 ± 3.9 $^{l}$ | - | 49.1 ± 1.1 $^{de}$ | 23.6 ± 0.5 $^{b}$ | 2.4 ± 0.1 $^{a}$ |
| EM20-I | 13.6 ± 0.1 $^{d}$ | 323.2 ± 4.2 $^{c}$ | 562.1 ± 11.0 $^{f}$ | - | 22.8 ± 0.8 $^{a}$ | 30.5 ± 0.2 $^{i}$ | 55.2 ± 1.4 $^{g}$ | 29.8 ± 0.6 $^{cd}$ | 3.0 ± 0.1 $^{cd}$ |
| EM20-II | 13.5 ± 0.2 $^{d}$ | 1449.2 ± 25.0 $^{m}$ | 513.3 ± 6.5 $^{d}$ | - | 30.9 ± 0.1 $^{def}$ | 10.0 ± 0.1 $^{c}$ | 62.8 ± 1.4 $^{j}$ | 18.5 ± 0.2 $^{a}$ | 3.0 ± 0.1 $^{cd}$ |
| EM20-III | 15.0 ± 0.1 $^{f}$ | 701.7 ± 1.6 $^{h}$ | 596.5 ± 9.9 $^{g}$ | - | 28.7 ± 0.3 $^{cd}$ | 4.8 ± 0.1 $^{a}$ | 72.9 ± 1.4 $^{l}$ | 21.0 ± 0.2 $^{b}$ | 3.1 ± 0.1 $^{d}$ |
| EM20-IV | 14.3 ± 0.1 $^{e}$ | 405.0 ± 6.2 $^{d}$ | 537.8 ± 6.1 $^{e}$ | - | 67.6 ± 0.2 $^{j}$ | - | 62.6 ± 1.2 $^{j}$ | 28.8 ± 0.5 $^{c}$ | 2.6 ± 0.1 $^{ab}$ |
| E+20-I | 11.3 ± 0.1 $^{b}$ | 999.7 ± 2.8 $^{j}$ | 529.4 ± 3.9 $^{de}$ | - | 22.5 ± 0.2 $^{a}$ | 15.4 ± 0.3 $^{e}$ | 42.7 ± 0.5 $^{a}$ | 31.6 ± 0.8 $^{de}$ | 2.9 ± 0.1 $^{c}$ |
| E+20-II | 14.8 ± 0.2 $^{f}$ | 1054.1 ± 38.1 $^{k}$ | 473.4 ± 2.0 $^{c}$ | - | 29.7 ± 0.1 $^{cde}$ | 19.6 ± 0.6 $^{f}$ | 46.3 ± 0.2 $^{bc}$ | 42.3 ± 1.0 $^{h}$ | 2.7 ± 0.1 $^{b}$ |
| E+20-III | 15.2 ± 0.1 $^{f}$ | 1051.0 ± 16.9 $^{k}$ | 681.1 ± 13.3 $^{ij}$ | - | 22.3 ± 0.7 $^{a}$ | 12.8 ± 0.1 $^{d}$ | 57.9 ± 0.9 $^{hi}$ | 35.2 ± 0.5 $^{fg}$ | 2.5 ± 0.1 $^{ab}$ |
| E+20-IV | 12.0 ± 0.2 $^{c}$ | 1367.0 ± 37.3 $^{l}$ | 397.8 ± 5.8 $^{b}$ | - | 23.5 ± 1.5 $^{a}$ | 6.9 ± 0.2 $^{b}$ | 67.7 ± 0.3 $^{k}$ | 37.3 ± 0.6 $^{g}$ | 3.1 ± 0.1 $^{d}$ |
| EL20-I | 12.2 ± 0.2 $^{c}$ | 1378.6 ± 20.1 $^{l}$ | 785.2 ± 14.6 $^{lm}$ | - | 35.3 ± 0.4 $^{gh}$ | 47.0 ± 1.4 $^{j}$ | 45.3 ± 0.7 $^{abc}$ | 45.1 ± 2.0 $^{i}$ | 2.5 ± 0.1 $^{ab}$ |
| EL20-II | 16.5 ± 0.2 $^{g}$ | 831.3 ± 3.3 $^{i}$ | 752.6 ± 20.1 $^{kl}$ | - | 32.6 ± 0.4 $^{efg}$ | 14.0 ± 0.8 $^{de}$ | 65.6 ± 1.6 $^{k}$ | 36.1 ± 0.5 $^{g}$ | 2.8 ± 0.1 $^{bc}$ |
| EL20-III | 17.0 ± 0.2 $^{g}$ | 541.5 ± 22.7 $^{fg}$ | 849.2 ± 13.3 $^{n}$ | - | 33.8 ± 0.1 $^{fg}$ | 8.1 ± 0.2 $^{b}$ | 51.4 ± 1.0 $^{ef}$ | 31.9 ± 0.5 $^{de}$ | 2.7 ± 0.1 $^{b}$ |
| EL20-IV | 17.2 ± 0.2 $^{h}$ | 540.4 ± 10.2 $^{fg}$ | 794.4 ± 6.3 $^{m}$ | - | 34.9 ± 0.4 $^{gh}$ | 7.7 ± 0.3 $^{b}$ | 59.1 ± 0.1 $^{i}$ | 30.8 ± 0.3 $^{cde}$ | 2.7 ± 0.1 $^{b}$ |
| PM20-I | 19.0 ± 0.1 $^{j}$ | 669.6 ± 5.7 $^{h}$ | 710.5 ± 6.7 $^{j}$ | - | 29.8 ± 0.1 $^{cde}$ | 24.3 ± 1.0 $^{h}$ | 43.6 ± 0.6 $^{ab}$ | 44.6 ± 1.4 $^{hi}$ | 2.7 ± 0.1 $^{b}$ |
| PM20-II | 18.2 ± 0.2 $^{i}$ | 569.6 ± 2.9 $^{g}$ | 693.5 ± 6.6 $^{j}$ | - | 24.2 ± 0.1 $^{ab}$ | 21.4 ± 0.1 $^{g}$ | 55.3 ± 0.4 $^{gh}$ | 32.9 ± 0.1 $^{ef}$ | 2.5 ± 0.1 $^{ab}$ |
| PM20-III | 16.3 ± 0.1 $^{g}$ | 517.5 ± 8.0 $^{ef}$ | 649.6 ± 12.2 $^{hi}$ | - | 27.3 ± 0.5 $^{bc}$ | 14.6 ± 0.4 $^{e}$ | 75.8 ± 1.5 $^{m}$ | 30.6 ± 0.6 $^{cde}$ | 2.4 ± 0.1 $^{a}$ |
| PM20-IV | 16.5 ± 0.2 $^{g}$ | 519.9 ± 4.0 $^{ef}$ | 633.2 ± 5.8 $^{h}$ | - | 29.9 ± 0.3 $^{cde}$ | 6.7 ± 0.3 $^{b}$ | 51.7 ± 1.0 $^{ef}$ | 32.1 ± 0.5 $^{de}$ | 2.6 ± 0.1 $^{ab}$ |

"-" not detected. Abbreviations: CS20−2020 vintage Cabernet Sauvignon sample prior storage; 20−vintage year 2020; S−stainless steel tank; EM−wooden barrel with excellent medium toasting; E+−wooden barrel with excellent medium plus toasting; EL−wooden barrel with excellent medium long toasting; PM−wooden barrel with premium medium toasting; I, II, III, IV−sampling after 3, 6, 9 and 12 months of ageing, respectively. Different superscript letters (a–n) in the same column indicate statistical difference determined by ANOVA, Fisher's (LSD) test with $p<0.05$.

**Table S4.** Alcohol concentrations in the aromatic profile of 2021 vintage Cabernet Sauvignon and samples obtained during 12-month storage in different vessels.

| Sample | Isoamyl alcohol (mg/L) | 2,3-butanediol (μg/L) | 1-hexanol (μg/L) | 1-heptanol (μg/L) | Methionol (μg/L) | 2-ethyl-1-hexanol (μg/L) | Benzyl alcohol (μg/L) | 1-octanol (μg/L) | 2-phenylethanol (mg/L) |
|---|---|---|---|---|---|---|---|---|---|
| CS21 | 11.1 ± 0.1 $^{f}$ | 490.7 ± 0.5 $^{d}$ | 376.2 ± 1.5 $^{e}$ | 3.7 ± 0.1 $^{bc}$ | 37.4 ± 0.1 $^{k}$ | 3.3 ± 0.1 $^{a}$ | 16.5 ± 0.4 $^{c}$ | 22.9 ± 0.3 $^{e}$ | 2.8 ± 0.1 $^{de}$ |
| S21-I | 14.2 ± 0.2 $^{i}$ | 469.0 ± 11.8 $^{d}$ | 940.3 ± 13.3 $^{k}$ | 3.1 ± 0.1 $^{ab}$ | 23.7 ± 0.2 $^{h}$ | 6.9 ± 0.2 $^{cd}$ | 16.9 ± 0.3 $^{c}$ | 28.5 ± 0.6 $^{hi}$ | 2.6 ± 0.1 $^{cd}$ |
| S21-II | 11.2 ± 0.1 $^{f}$ | 337.2 ± 21.5 $^{c}$ | 987.6 ± 1.2 $^{l}$ | 7.0 ± 0.7 $^{e}$ | 48.3 ± 1.3 $^{m}$ | 8.3 ± 0.1 $^{de}$ | 21.5 ± 0.1 $^{g}$ | 27.7 ± 0.5 $^{h}$ | 2.7± 0.1 $^{d}$ |
| S21-III | 7.6 ± 0.1 $^{b}$ | 279.3 ± 6.4 $^{b}$ | 947.8 ± 4.4 $^{k}$ | 11.1 ± 0.3 $^{gh}$ | 35.4 ± 0.8 $^{k}$ | 3.5 ± 0.1 $^{a}$ | 13.6 ± 0.4 $^{b}$ | 24.8 ± 0.3 $^{fg}$ | 2.4 ± 0.1 $^{bc}$ |
| S21-IV | 5.6 ± 0.1 $^{a}$ | 199.3 ± 2.8 $^{a}$ | 799.3 ± 0.7 $^{j}$ | 13.5 ± 0.1 $^{ij}$ | 44.5 ± 0.2 $^{l}$ | 2.9 ± 0.2 $^{a}$ | 12.3 ± 0.1 $^{a}$ | 23.1 ± 0.2 $^{e}$ | 2.2 ± 0.1 $^{ab}$ |
| EM21-I | 18.1 ± 0.1 $^{l}$ | 595.5 ± 0.1 $^{f}$ | 775.2 ± 4.6 $^{j}$ | 4.0 ± 0.1 $^{c}$ | 30.3 ± 0.8 $^{i}$ | 15.8 ± 0.1 $^{g}$ | 28.4 ± 0.1 $^{j}$ | 22.5 ± 0.4 $^{e}$ | 3.4 ± 0.1 $^{g}$ |
| EM21-II | 16.9 ± 0.1 $^{k}$ | 475.2 ± 13.6 $^{d}$ | 553.0 ± 2.4 $^{h}$ | 3.1 ± 0.1 $^{ab}$ | 18.4 ± 0.1 $^{de}$ | 12.0 ± 0.7 $^{f}$ | 26.7 ± 0.3 $^{i}$ | 29.8 ± 0.1 $^{i}$ | 3.0 ± 0.1 $^{ef}$ |
| EM21-III | 11.1 ± 0.2 $^{f}$ | 552.6 ± 1.9 $^{e}$ | 210.5 ± 5.5 $^{c}$ | 3.2 ± 0.1 $^{ab}$ | 32.9 ± 0.8 $^{j}$ | 5.2 ± 0.1 $^{b}$ | 19.0 ± 0.3 $^{ef}$ | 21.3 ± 0.3 $^{d}$ | 2.6 ± 0.1 $^{cd}$ |
| EM21-IV | 9.7 ± 0.1 $^{d}$ | 573.6 ± 3.0 $^{ef}$ | 174.9 ± 3.7 $^{b}$ | 2.6 ± 0.1 $^{a}$ | 29.5 ± 0.3 $^{i}$ | 2.4 ± 0.1 $^{a}$ | 17.1 ± 0.1 $^{c}$ | 23.4 ± 0.4 $^{e}$ | 2.3 ± 0.1 $^{b}$ |
| E+21-I | 17.8 ± 0.2 $^{l}$ | 639.6 ± 18.8 $^{g}$ | 394.2 ± 11.5 $^{e}$ | 5.6 ± 0.2 $^{d}$ | 44.6 ± 0.6 $^{l}$ | 26.8 ± 1.2 $^{i}$ | 19.4 ± 0.1 $^{ef}$ | 18.0 ± 0.3 $^{ab}$ | 2.6 ± 0.1 $^{cd}$ |
| E+21-II | 13.1 ± 0.1 $^{h}$ | 579.0 ± 5.7 $^{ef}$ | 501.6 ± 6.2 $^{f}$ | 11.5 ± 0.1 $^{h}$ | 21.0 ± 0.1 $^{fg}$ | 14.4 ± 0.2 $^{g}$ | 24.5 ± 0.4 $^{h}$ | 24.9 ± 0.5 $^{fg}$ | 2.7 ± 0.1 $^{d}$ |
| E+21-III | 10.4 ± 0.3 $^{e}$ | 804.3 ± 1.3 $^{h}$ | 203.7 ± 3.9 $^{c}$ | 13.1 ± 0.3 $^{i}$ | 23.0 ± 0.1 $^{gh}$ | 7.6 ± 0.4 $^{d}$ | 28.9 ± 0.5 $^{j}$ | 25.9 ± 0.8 $^{g}$ | 2.8 ± 0.1 $^{de}$ |
| E+21-IV | 8.2 ± 0.1 $^{c}$ | 855.5 ± 11.0 $^{i}$ | 177.2 ± 1.2 $^{b}$ | 18.1 ± 0.1 $^{l}$ | 12.5 ± 0.1 $^{a}$ | 5.2 ± 0.1 $^{b}$ | 32.9 ± 1.1 $^{k}$ | 29.9 ± 0.9 $^{i}$ | 2.9 ± 0.1 $^{e}$ |
| EL21-I | 15.9 ± 0.1 $^{j}$ | 770.0 ± 25.0 $^{h}$ | 509.8 ± 7.4 $^{fg}$ | 10.8 ± 0.3 $^{g}$ | 20.3 ± 0.6 $^{ef}$ | 39.2 ± 1.5 $^{k}$ | 19.7 ± 0.2 $^{ef}$ | 20.3 ± 0.1 $^{c}$ | 2.6 ± 0.1 $^{cd}$ |
| EL21-II | 12.9 ± 0.1 $^{h}$ | 795.0 ± 1.8 $^{h}$ | 530.1 ± 17.3 $^{gh}$ | 10.7 ± 0.4 $^{g}$ | 17.3 ± 0.6 $^{cd}$ | 18.0 ± 0.5 $^{h}$ | 24.6 ± 0.1 $^{h}$ | 21.9 ± 0.9 $^{de}$ | 2.6 ± 0.1 $^{cd}$ |
| EL21-III | 11.9 ± 0.1 $^{g}$ | 972.5 ± 11.4 $^{j}$ | 237.2 ± 6.5 $^{d}$ | 13.4 ± 0.2 $^{ij}$ | 15.2 ± 0.2 $^{bc}$ | 9.4 ± 0.1 $^{e}$ | 17.5 ± 0.4 $^{cd}$ | 17.9 ± 0.5 $^{ab}$ | 2.3 ± 0.1 $^{b}$ |
| EL21-IV | 10.5 ± 0.1 $^{e}$ | 1039.7 ± 24.2 $^{k}$ | 208.5 ± 5.3 $^{c}$ | 14.6 ± 0.2 $^{k}$ | 13.5 ± 0.1 $^{ab}$ | 7.7 ± 0.3 $^{d}$ | 18.6 ± 0.2 $^{de}$ | 18.7 ± 0.2 $^{b}$ | 2.3 ± 0.1 $^{b}$ |
| PM21-I | 11.9 ± 0.1 $^{g}$ | 348.1 ± 5.4 $^{c}$ | 110.2 ± 3.9 $^{a}$ | 9.4 ± 0.3 $^{f}$ | 24.7 ± 0.6 $^{h}$ | 33.1 ± 0.7 $^{j}$ | 26.9 ± 0.8 $^{i}$ | 24.2 ± 0.1 $^{f}$ | 3.1 ± 0.1 $^{f}$ |
| PM21-II | 10.2 ± 0.1 $^{e}$ | 1290.7 ± 28.0 $^{m}$ | 545.7 ± 5.5 $^{h}$ | 9.2 ± 0.1 $^{f}$ | 44.9 ± 2.5 $^{l}$ | 7.9 ± 0.3 $^{d}$ | 19.5 ± 0.4 $^{ef}$ | 20.9 ± 0.1 $^{cd}$ | 2.4 ± 0.1 $^{bc}$ |
| PM21-III | 10.3 ± 0.1 $^{e}$ | 1236.5 ± 25.9 $^{l}$ | 692.9 ± 18.9 $^{i}$ | 13.3 ± 0.2 $^{ij}$ | 23.3 ± 0.5 $^{h}$ | 7.8 ± 0.1 $^{d}$ | 20.0 ± 0.3 $^{f}$ | 18.9 ± 0.3 $^{b}$ | 2.5 ± 0.1 $^{c}$ |
| PM21-IV | 9.6 ± 0.1 $^{d}$ | 1684.3 ± 3.2 $^{n}$ | 937.0 ± 17.2 $^{k}$ | 13.9 ± 0.1 $^{jk}$ | 30.5 ± 0.1 $^{i}$ | 5.5 ± 0.4 $^{bc}$ | 16.5 ± 0.3 $^{c}$ | 16.9 ± 0.6 $^{a}$ | 2.1 ± 0.1 $^{a}$ |

Abbreviations: CS21−2021 vintage Cabernet Sauvignon sample prior storage; 21−vintage year 2021; S−stainless steel tank; EM−wooden barrel with excellent medium toasting; E+−wooden barrel with excellent medium plus toasting; EL−wooden barrel with excellent medium long toasting; PM−wooden barrel with premium medium toasting; I, II, III, IV−sampling after 3, 6, 9 and 12 months of ageing, respectively. Different superscript letters (a−n) in the same column indicate statistical difference determined by ANOVA, Fisher's (LSD) test with $p<0.05$.

**Table S5.** Carbonyl compound, volatile phenol and lactone concentrations (μg/L) in the aromatic profile of 2020 vintage Cabernet Sauvignon and samples obtained during 12-month storage in different vessels.

| Sample | Carbonyl compounds | | | | Volatile phenols and lactones | | | | | | |
|---|---|---|---|---|---|---|---|---|---|---|---|
| | Benzaldehyde | Geranyl acetone | Myristaldehyde | Hexyl cinnamaldehyde | γ-butyrolactone | 4-ethyl phenol | 4-ethyl guaiacol | Cis-whiskey lactone | γ-heptalactone | Trans-whiskey lactone | γ-nonalactone |
| CS20 | - | - | $6.6 \pm 0.1^{g}$ | $4.9 \pm 0.4^{ef}$ | - | - | - | - | - | - | - |
| S20-I | - | - | $4.2 \pm 0.1^{d}$ | $5.4 \pm 0.1^{fg}$ | $9.2 \pm 0.3^{b}$ | - | - | - | - | - | - |
| S20-II | - | - | $8.5 \pm 0.1^{h}$ | $6.1 \pm 0.1^{g}$ | $16.1 \pm 0.2^{d}$ | - | - | - | - | - | - |
| S20-III | - | - | $9.7 \pm 0.1^{i}$ | $3.1 \pm 0.1^{c}$ | $9.6 \pm 0.1^{b}$ | - | - | - | - | - | - |
| S20-IV | - | - | $5.9 \pm 0.1^{f}$ | $4.4 \pm 0.1^{de}$ | $6.6 \pm 0.1^{a}$ | - | - | - | - | - | - |
| EM20-I | - | - | $10.0 \pm 0.1^{i}$ | $2.4 \pm 0.1^{bc}$ | $15.4 \pm 0.3^{cd}$ | $23.6 \pm 0.1^{a}$ | - | $2.9 \pm 0.1^{a}$ | $6.5 \pm 0.1^{d}$ | $19.1 \pm 0.5^{b}$ | $4.4 \pm 0.2^{a}$ |
| EM20-II | - | - | $12.9 \pm 0.1^{j}$ | $2.3 \pm 0.1^{b}$ | $23.0 \pm 0.6^{g}$ | $61.5 \pm 0.8^{h}$ | - | $3.0 \pm 0.1^{a}$ | $6.4 \pm 0.1^{d}$ | $31.2 \pm 0.3^{e}$ | $6.8 \pm 0.1^{c}$ |
| EM20-III | - | - | $2.0 \pm 0.1^{b}$ | $3.9 \pm 0.3^{d}$ | $27.0 \pm 0.1^{i}$ | $105.1 \pm 0.2^{k}$ | - | $6.1 \pm 0.2^{d}$ | $9.9 \pm 0.5^{f}$ | $55.9 \pm 0.1^{i}$ | $8.6 \pm 0.1^{e}$ |
| EM20-IV | - | - | $5.0 \pm 0.2^{e}$ | $2.1 \pm 0.3^{ab}$ | $35.7 \pm 1.4^{j}$ | $66.2 \pm 0.2^{i}$ | - | $7.1 \pm 0.2^{e}$ | $11.2 \pm 0.1^{g}$ | $63.9 \pm 0.8^{j}$ | $10.2 \pm 0.3^{f}$ |
| E+20-I | - | - | $3.3 \pm 0.3^{c}$ | $1.5 \pm 0.1^{a}$ | $14.4 \pm 0.2^{c}$ | $34.5 \pm 1.4^{b}$ | - | $6.6 \pm 0.1^{de}$ | $6.7 \pm 0.1^{d}$ | $27.1 \pm 0.1^{d}$ | $4.6 \pm 0.2^{a}$ |
| E+20-II | - | - | $1.6 \pm 0.1^{a}$ | $2.6 \pm 0.2^{bc}$ | $21.4 \pm 0.9^{f}$ | $52.9 \pm 0.4^{e}$ | $8.7 \pm 0.1^{cd}$ | $8.0 \pm 0.1^{f}$ | $8.8 \pm 0.1^{e}$ | $33.6 \pm 0.1^{f}$ | $6.1 \pm 0.1^{b}$ |
| E+20-III | - | - | $4.2 \pm 0.2^{d}$ | $2.6 \pm 0.1^{bc}$ | $35.1 \pm 0.6^{j}$ | $91.3 \pm 1.5^{j}$ | $8.0 \pm 0.2^{b}$ | $12.0 \pm 0.1^{g}$ | $13.4 \pm 0.2^{h}$ | $45.5 \pm 0.4^{h}$ | $6.0 \pm 0.1^{b}$ |
| E+20-IV | - | - | $3.1 \pm 0.1^{c}$ | $3.1 \pm 0.1^{c}$ | $38.6 \pm 0.3^{k}$ | $64.2 \pm 0.1^{i}$ | $10.6 \pm 0.4^{e}$ | $13.7 \pm 0.1^{h}$ | $16.8 \pm 0.1^{i}$ | $55.0 \pm 2.0^{i}$ | $8.6 \pm 0.2^{e}$ |
| EL20-I | $6.9 \pm 0.2^{a}$ | - | $8.5 \pm 0.1^{h}$ | $8.8 \pm 0.5^{j}$ | $15.2 \pm 0.3^{cd}$ | $44.2 \pm 1.6^{d}$ | $3.4 \pm 0.1^{a}$ | $4.1 \pm 0.1^{b}$ | $3.8 \pm 0.1^{a}$ | $14.0 \pm 0.3^{j}$ | $4.8 \pm 0.1^{a}$ |
| EL20-II | $7.3 \pm 0.1^{a}$ | - | - | $8.1 \pm 0.1^{hi}$ | $18.0 \pm 0.2^{e}$ | $59.7 \pm 0.4^{gh}$ | $8.5 \pm 0.1^{bcd}$ | $5.5 \pm 0.2^{c}$ | $5.6 \pm 0.1^{c}$ | $19.1 \pm 0.1^{b}$ | $5.8 \pm 0.3^{b}$ |
| EL20-III | $9.4 \pm 0.1^{b}$ | - | - | $5.8 \pm 0.1^{g}$ | $20.9 \pm 0.4^{f}$ | $55.9 \pm 0.9^{f}$ | $8.2 \pm 0.1^{bc}$ | $7.1 \pm 0.2^{e}$ | $6.9 \pm 0.4^{d}$ | $21.0 \pm 0.2^{bc}$ | $8.7 \pm 0.3^{e}$ |
| EL20-IV | $9.8 \pm 0.2^{b}$ | - | - | $5.8 \pm 0.2^{g}$ | $22.0 \pm 0.1^{fg}$ | $53.6 \pm 0.3^{ef}$ | $8.2 \pm 0.2^{bc}$ | $8.0 \pm 0.1^{f}$ | $8.3 \pm 0.1^{e}$ | $22.3 \pm 0.2^{c}$ | $10.4 \pm 0.2^{fg}$ |
| PM20-I | $10.8 \pm 0.1^{d}$ | - | - | $10.9 \pm 0.3^{k}$ | $16.3 \pm 0.4^{d}$ | $37.9 \pm 1.1^{c}$ | $9.0 \pm 0.1^{d}$ | $17.1 \pm 0.3^{i}$ | $4.9 \pm 0.1^{b}$ | $42.4 \pm 0.9^{g}$ | $5.8 \pm 0.3^{b}$ |
| PM20-II | $9.9 \pm 0.4^{bc}$ | - | - | $8.5 \pm 0.2^{ij}$ | $16.4 \pm 0.2^{d}$ | $53.8 \pm 0.2^{ef}$ | $10.9 \pm 0.2^{ef}$ | $23.5 \pm 0.1^{j}$ | $6.4 \pm 0.1^{d}$ | $57.0 \pm 1.0^{i}$ | $7.6 \pm 0.1^{d}$ |
| PM20-III | $10.5 \pm 0.1^{c}$ | - | - | $7.5 \pm 0.3^{h}$ | $22.3 \pm 0.1^{fg}$ | $59.1 \pm 0.2^{g}$ | $11.1 \pm 0.1^{f}$ | $24.8 \pm 0.2^{k}$ | $8.5 \pm 0.1^{e}$ | $68.7 \pm 0.7^{k}$ | $9.9 \pm 0.2^{f}$ |
| PM20-IV | $10.4 \pm 0.1^{c}$ | - | - | $8.5 \pm 0.5^{ij}$ | $24.6 \pm 0.3^{h}$ | $63.9 \pm 0.1^{i}$ | $14.7 \pm 0.1^{g}$ | $35.4 \pm 0.5^{l}$ | $9.6 \pm 0.3^{f}$ | $83.3 \pm 0.5^{l}$ | $11.0 \pm 0.3^{g}$ |

"-" not detected. Abbreviations: CS20−2020 vintage Cabernet Sauvignon sample prior storage; 20−vintage year 2020; S−stainless steel tank; EM−wooden barrel with excellent medium toasting; E+−wooden barrel with excellent medium plus toasting; EL−wooden barrel with excellent medium long toasting; PM−wooden barrel with premium medium toasting; I, II, III, IV−sampling after 3, 6, 9 and 12 months of ageing, respectively. Different superscript letters (a–l) in the same column indicate statistical difference determined by ANOVA, Fisher's (LSD) test with $p<0.05$.

**Table S6.** Carbonyl compound, volatile phenol and lactone concentrations (μg/L) in the aromatic profile of 2021 vintage Cabernet Sauvignon and samples obtained during 12-month storage in different vessels.

| Sample | Carbonyl compounds | | | | Volatile phenols and lactones | | | | | | |
|---|---|---|---|---|---|---|---|---|---|---|---|
| | Benzaldehyde | Geranyl acetone | Myristaldehyde | Hexyl cinnamaldehyde | γ-butyrolactone | 4-ethyl phenol | 4-ethyl guaiacol | Cis-whiskey lactone | γ-heptalactone | Trans-whiskey lactone | γ-nonalactone |
| CS21 | - | 17.8 ± 0.4$^{j}$ | 3.8 ± 0.1$^{def}$ | 3.5 ± 0.3$^{ab}$ | - | - | - | - | - | - | - |
| S21-I | - | 19.6 ± 0.3$^{k}$ | 11.5 ± 0.3$^{j}$ | 7.8 ± 0.2$^{g}$ | 5.5 ± 0.2$^{a}$ | - | - | - | - | - | - |
| S21-II | - | 14.9 ± 0.2$^{h}$ | 3.5 ± 0.1$^{cd}$ | 4.0 ± 0.1$^{bc}$ | 7.8 ± 0.1$^{b}$ | - | - | - | - | - | - |
| S21-III | - | 12.2 ± 0.3$^{f}$ | 3.5 ± 0.1$^{c}$ | 5.2 ± 0.3$^{de}$ | 11.7 ± 0.4$^{d}$ | - | - | - | - | - | - |
| S21-IV | - | 9.4 ± 0.3$^{c}$ | 3.4 ± 0.1$^{c}$ | 3.1 ± 0.2$^{a}$ | 13.5 ± 0.2$^{e}$ | - | - | - | - | - | - |
| EM21-I | - | 12.8 ± 0.1$^{f}$ | 3.5 ± 0.1$^{cd}$ | 3.9 ± 0.1$^{bc}$ | 10.1 ± 0.1$^{cd}$ | 15.7 ± 0.2$^{f}$ | - | 2.2 ± 0.2$^{b}$ | 3.4 ± 0.1$^{de}$ | 13.4 ± 0.7$^{a}$ | 5.5 ± 0.5$^{f}$ |
| EM21-II | - | 14.0 ± 0.1$^{g}$ | 5.0 ± 0.2$^{g}$ | 6.8 ± 0.3$^{f}$ | 21.5 ± 0.1$^{gh}$ | 15.5 ± 0.2$^{f}$ | - | 3.3 ± 0.1$^{cd}$ | 3.7 ± 0.1$^{ef}$ | 41.4 ± 0.5$^{g}$ | 7.9 ± 0.1$^{h}$ |
| EM21-III | - | 11.3 ± 0.3$^{e}$ | 4.0 ± 0.1$^{ef}$ | 8.3 ± 0.1$^{g}$ | 25.5 ± 0.5$^{i}$ | 15.7 ± 0.2$^{f}$ | - | 3.7 ± 0.1$^{de}$ | 4.5 ± 0.2$^{h}$ | 49.8 ± 1.0$^{i}$ | 10.6 ± 0.3$^{i}$ |
| EM21-IV | - | 10.3 ± 0.2$^{d}$ | 3.4 ± 0.1$^{c}$ | 10.2 ± 0.5$^{i}$ | 33.0 ± 1.7$^{k}$ | 15.6 ± 0.1$^{f}$ | - | 4.4 ± 0.2$^{f}$ | 4.9 ± 0.1$^{i}$ | 62.9 ± 0.9$^{j}$ | 12.2 ± 0.1$^{j}$ |
| E+21-I | - | 6.1 ± 0.2$^{a}$ | 3.6 ± 0.3$^{cd}$ | 4.2 ± 0.3$^{c}$ | 7.4 ± 0.4$^{b}$ | 10.1 ± 0.3$^{b}$ | - | 1.7 ± 0.1$^{a}$ | 1.4 ± 0.1$^{a}$ | 34.8 ± 0.1$^{f}$ | 2.0 ± 0.1$^{a}$ |
| E+21-II | - | 11.3 ± 0.1$^{e}$ | 3.5 ± 0.1$^{cd}$ | 5.6 ± 0.4$^{de}$ | 13.7 ± 0.4$^{e}$ | 11.7 ± 0.1$^{cd}$ | - | 2.9 ± 0.1$^{c}$ | 1.7 ± 0.1$^{a}$ | 39.8 ± 0.3$^{g}$ | 2.5 ± 0.1$^{a}$ |
| E+21-III | - | 12.6 ± 0.2$^{f}$ | 5.5 ± 0.1$^{h}$ | 10.9 ± 0.2$^{i}$ | 22.6 ± 0.3$^{h}$ | 10.0 ± 0.2$^{b}$ | - | 5.2 ± 0.1$^{g}$ | 2.1 ± 0.1$^{b}$ | 45.2 ± 0.2$^{h}$ | 3.2 ± 0.2$^{b}$ |
| E+21-IV | - | 15.5 ± 0.2$^{hi}$ | 6.1 ± 0.1$^{i}$ | 12.6 ± 0.3$^{j}$ | 29.4 ± 0.7$^{j}$ | 15.2 ± 0.1$^{f}$ | - | 6.1 ± 0.1$^{h}$ | 2.5 ± 0.1$^{c}$ | 49.6 ± 1.2$^{i}$ | 3.8 ± 0.1$^{c}$ |
| EL21-I | - | 11.2 ± 0.6$^{e}$ | 3.7 ± 0.2$^{cde}$ | 5.0 ± 0.1$^{d}$ | 8.8 ± 0.1$^{bc}$ | 12.7 ± 0.1$^{de}$ | - | 2.2 ± 0.1$^{b}$ | 3.2 ± 0.1$^{d}$ | 28.0 ± 0.8$^{d}$ | 3.4 ± 0.2$^{bc}$ |
| EL21-II | - | 12.5 ± 0.1$^{f}$ | 3.4 ± 0.1$^{c}$ | 9.1 ± 0.3$^{h}$ | 11.5 ± 0.3$^{d}$ | 12.6 ± 0.3$^{de}$ | - | 3.1 ± 0.1$^{c}$ | 4.5 ± 0.1$^{h}$ | 30.5 ± 0.4$^{e}$ | 4.6 ± 0.2$^{de}$ |
| EL21-III | - | 15.6 ± 0.1$^{i}$ | 2.4 ± 0.1$^{b}$ | 5.2 ± 0.1$^{de}$ | 17.0 ± 0.5$^{f}$ | 12.7 ± 0.1$^{de}$ | - | 3.8 ± 0.1$^{e}$ | 5.2 ± 0.1$^{ij}$ | 45.0 ± 1.2$^{h}$ | 6.1 ± 0.1$^{g}$ |
| EL21-IV | - | 17.5 ± 0.1$^{j}$ | 1.9 ± 0.1$^{a}$ | 6.6 ± 0.1$^{f}$ | 20.0 ± 0.9$^{g}$ | 15.5 ± 0.3$^{f}$ | - | 4.5 ± 0.2$^{f}$ | 6.2 ± 0.2$^{k}$ | 51.6 ± 1.0$^{i}$ | 7.6 ± 0.1$^{h}$ |
| PM21-I | 4.3 ± 0.1$^{a}$ | 8.3 ± 0.1$^{b}$ | 5.2 ± 0.2$^{gh}$ | 5.8 ± 0.1$^{e}$ | 21.3 ± 0.5$^{gh}$ | 7.3 ± 0.1$^{a}$ | - | 7.8 ± 0.2$^{i}$ | 3.9 ± 0.1$^{fg}$ | 13.7 ± 0.3$^{a}$ | 3.2 ± 0.1$^{b}$ |
| PM21-II | 5.0 ± 0.1$^{b}$ | 15.7 ± 0.1$^{i}$ | 4.1 ± 0.1$^{f}$ | 8.2 ± 0.2$^{g}$ | 25.8 ± 0.9$^{i}$ | 13.2 ± 1.2$^{e}$ | - | 9.3 ± 0.4$^{j}$ | 4.2 ± 0.1$^{g}$ | 18.8 ± 0.1$^{b}$ | 3.9 ± 0.1$^{c}$ |
| PM21-III | 5.7 ± 0.1$^{c}$ | 7.7 ± 0.1$^{b}$ | 2.7 ± 0.1$^{b}$ | 8.3 ± 0.2$^{g}$ | 30.5 ± 0.1$^{j}$ | 15.8 ± 0.4$^{f}$ | - | 10.6 ± 0.1$^{k}$ | 5.1 ± 0.1$^{i}$ | 20.2 ± 0.4$^{b}$ | 4.5 ± 0.1$^{d}$ |
| PM21-IV | 6.8 ± 0.8$^{d}$ | 10.0 ± 0.1$^{cd}$ | 1.9 ± 0.1$^{a}$ | 8.4 ± 0.3$^{g}$ | 34.1 ± 0.6$^{k}$ | 20.2 ± 0.4$^{g}$ | - | 11.8 ± 0.3$^{l}$ | 5.5 ± 0.1$^{j}$ | 24.2 ± 0.3$^{c}$ | 5.1 ± 0.1$^{ef}$ |

"-" not detected. Abbreviations: CS21−2021 vintage Cabernet Sauvignon sample prior storage; 21−vintage year 2021; S−stainless steel tank; EM−wooden barrel with excellent medium toasting; E+−wooden barrel with excellent medium plus toasting; EL−wooden barrel with excellent medium long toasting; PM−wooden barrel with premium medium toasting; I, II, III, IV−sampling after 3, 6, 9 and 12 months of ageing, respectively. Different superscript letters (a−k) in the same column indicate statistical difference determined by ANOVA, Fisher's (LSD) test with $p<0.05$.

**Table S7.** Ester concentrations (µg/L) in the aromatic profile of 2020 vintage Cabernet Sauvignon and samples obtained during 12-month storage in different vessels.

| Sample | Ethyl hexanoate | Ethyl 4-hydroxybutanoate | Isoamyl lactate | Diethyl succinate | Ethyl octanoate | Phenethyl acetate | Ethyl decanoate | Ethyl cinnamate |
|---|---|---|---|---|---|---|---|---|
| CS20 | 117.6 ± 0.1 $^{l}$ | 20.4 ± 0.1 $^{g}$ | - | 1026.6 ± 21.8 $^{d}$ | 251.6 ± 5.0 $^{k}$ | 36.7 ± 0.9 $^{g}$ | 30.8 ± 0.2 $^{m}$ | - |
| S20-I | 127.1 ± 2.0 $^{m}$ | - | - | 756.7 ± 22.0 $^{b}$ | 178.3 ± 2.9 $^{h}$ | 27.1 ± 0.2 $^{b}$ | 23.0 ± 0.2 $^{efg}$ | - |
| S20-II | 186.7 ± 3.3 $^{o}$ | - | - | 1023.3 ± 8.2 $^{d}$ | 147.6 ± 1.7 $^{g}$ | 27.9 ± 0.3 $^{bc}$ | 21.4 ± 0.4 $^{c}$ | - |
| S20-III | 113.1 ± 1.3 $^{k}$ | - | - | 718.4 ± 6.2 $^{ab}$ | 136.3 ± 3.0 $^{ef}$ | 21.5 ± 0.9 $^{a}$ | 23.4 ± 0.4 $^{fgh}$ | - |
| S20-IV | 149.5 ± 0.8 $^{n}$ | - | - | 665.5 ± 2.8 $^{a}$ | 204.1 ± 2.8 $^{i}$ | 31.4 ± 0.1 $^{e}$ | 24.6 ± 0.3 $^{i}$ | - |
| EM20-I | 71.1 ± 1.4 $^{e}$ | - | 103.7 ± 2.5 $^{a}$ | 1108.1 ± 34.6 $^{ef}$ | 145.6 ± 3.0 $^{g}$ | 29.1 ± 0.6 $^{cd}$ | 22.3 ± 0.6 $^{cde}$ | - |
| EM20-II | 60.3 ± 0.7 $^{d}$ | - | 123.4 ± 0.8 $^{bc}$ | 1203.5 ± 11.9 $^{hi}$ | 100.8 ± 0.2 $^{b}$ | 26.2 ± 0.1 $^{b}$ | 22.2 ± 0.3 $^{cde}$ | - |
| EM20-III | 49.1 ± 0.7 $^{c}$ | - | 131.6 ± 0.9 $^{d}$ | 1027.2 ± 22.9 $^{d}$ | 99.9 ± 4.8 $^{b}$ | 26.8 ± 0.1 $^{b}$ | 22.6 ± 0.2 $^{def}$ | - |
| EM20-IV | 106.2 ± 0.2 $^{j}$ | - | 140.7 ± 0.2 $^{f}$ | 847.6 ± 3.0 $^{c}$ | 128.0 ± 6.1 $^{cd}$ | 30.4 ± 1.0 $^{de}$ | 22.4 ± 0.4 $^{de}$ | - |
| E+20-I | 14.6 ± 0.5 $^{b}$ | 11.5 ± 0.1 $^{cd}$ | 124.0 ± 1.7 $^{bc}$ | 1068.5 ± 12.5 $^{de}$ | 186.6 ± 1.1 $^{h}$ | 30.1 ± 0.6 $^{de}$ | 14.5 ± 0.1 $^{a}$ | - |
| E+20-II | 10.4 ± 0.2 $^{ab}$ | 14.9 ± 0.6 $^{f}$ | 138.1 ± 0.8 $^{ef}$ | 1209.8 ± 29.9 $^{hij}$ | 186.1 ± 1.9 $^{h}$ | 34.0 ± 1.0 $^{f}$ | 17.2 ± 0.2 $^{b}$ | - |
| E+20-III | 9.2 ± 0.1 $^{a}$ | 15.1 ± 0.3 $^{f}$ | 148.2 ± 1.7 $^{g}$ | 1358.5 ± 7.6 $^{k}$ | 123.9 ± 1.7 $^{c}$ | 39.6 ± 1.2 $^{h}$ | 21.9 ± 0.5 $^{cd}$ | - |
| E+20-IV | 10.2 ± 0.1 $^{ab}$ | 20.1 ± 0.7 $^{g}$ | 166.2 ± 2.0 $^{j}$ | 1250.7 ± 9.6 $^{ij}$ | 132.4 ± 3.3 $^{de}$ | 32.0 ± 0.3 $^{ef}$ | 26.8 ± 0.1 $^{j}$ | - |
| EL20-I | 95.2 ± 1.9 $^{i}$ | 10.1 ± 0.8 $^{ab}$ | 137.0 ± 0.3 $^{e}$ | 1101.1 ± 18.9 $^{ef}$ | 83.6 ± 0.8 $^{a}$ | 42.9 ± 1.0 $^{i}$ | 26.5 ± 0.3 $^{j}$ | 6.3 ± 0.1 $^{b}$ |
| EL20-II | 90.1 ± 1.5 $^{h}$ | 14.3 ± 0.2 $^{f}$ | 146.2 ± 0.6 $^{g}$ | 1177.5 ± 10.8 $^{gh}$ | 90.5 ± 1.2 $^{a}$ | 45.0 ± 0.1 $^{jk}$ | 24.2 ± 0.3 $^{hi}$ | 6.6 ± 0.1 $^{c}$ |
| EL20-III | 107.5 ± 0.7 $^{j}$ | 15.3 ± 0.1 $^{f}$ | 146.6 ± 0.1 $^{g}$ | 1173.3 ± 26.3 $^{gh}$ | 90.2 ± 0.4 $^{a}$ | 47.5 ± 0.2 $^{l}$ | 26.8 ± 0.4 $^{jk}$ | 6.6 ± 0.1 $^{b c}$ |
| EL20-IV | 90.4 ± 1.4 $^{h}$ | 12.3 ± 0.2 $^{de}$ | 160.9 ± 1.4 $^{i}$ | 1263.6 ± 14.6 $^{j}$ | 91.3 ± 1.1 $^{a}$ | 47.3 ± 0.1 $^{l}$ | 29.7 ± 0.2 $^{l}$ | 7.9 ± 0.1 $^{d}$ |
| PM20-I | 96.3 ± 3.7 $^{i}$ | 9.3 ± 0.1 $^{a}$ | 120.9 ± 0.6 $^{b}$ | 1130.4 ± 25.4 $^{fg}$ | 217.3 ± 1.3 $^{j}$ | 43.1 ± 0.6 $^{ij}$ | 27.7 ± 0.3 $^{k}$ | 8.2 ± 0.1 $^{d}$ |
| PM20-II | 75.9 ± 2.3 $^{fg}$ | 10.7 ± 0.1 $^{bc}$ | 126.9 ± 1.2 $^{c}$ | 1103.4 ± 18.2 $^{ef}$ | 142.7 ± 0.2 $^{fg}$ | 40.0 ± 0.1 $^{h}$ | 23.9 ± 0.1 $^{ghi}$ | 2.7 ± 0.1 $^{a}$ |
| PM20-III | 80.2 ± 1.3 $^{g}$ | 10.0 ± 0.1 $^{ab}$ | 152.0 ± 0.3 $^{h}$ | 1131.7 ± 2.7 $^{fg}$ | 132.8 ± 1.0 $^{de}$ | 36.2 ± 0.1 $^{g}$ | 22.5 ± 0.3 $^{de}$ | - |
| PM20-IV | 73.1 ± 0.9 $^{ef}$ | 12.7 ± 0.5 $^{e}$ | 177.6 ± 1.4 $^{k}$ | 1249.6 ± 29.4 $^{ij}$ | 133.4 ± 2.5 $^{de}$ | 45.5 ± 0.5 $^{jk}$ | 30.6 ± 0.1 $^{m}$ | - |

**Table S7.** Continue.

| Sample | Ethyl vanillate | Ethyl laurate | Methyl dihydrojasmonate | Ethyl myristate | Ethyl palmitate | Ethyl linoleate | Ethyl oleate | Ethyl stearate |
|---|---|---|---|---|---|---|---|---|
| CS20 | 12.0 ± 0.5 $^{gh}$ | 4.6 ± 0.1 $^{b}$ | 9.1 ± 0.1 $^{j}$ | 13.1 ± 0.1 $^{j}$ | 44.4 ± 3.8 $^{a}$ | - | 5.6 ± 0.1 $^{ij}$ | 32.3 ± 0.6 $^{ij}$ |
| S20-I | 7.8 ± 0.1 $^{c}$ | 10.1 ± 0.2 $^{j}$ | 6.6 ± 0.2 $^{h}$ | 9.9 ± 0.5 $^{g}$ | 111.3 ± 0.8 $^{g}$ | 4.5 ± 0.2 $^{de}$ | 7.0 ± 0.1 $^{k}$ | 13.0 ± 0.1 $^{cde}$ |
| S20-II | 7.9 ± 0.2 $^{c}$ | 9.9 ± 0.1 $^{j}$ | 7.8 ± 0.2 $^{i}$ | 9.0 ± 0.2 $^{g}$ | 123.6 ± 1.7 $^{h}$ | 3.8 ± 0.1 $^{bc}$ | 5.1 ± 0.1 $^{hi}$ | 13.8 ± 0.2 $^{de}$ |
| S20-III | 17.2 ± 0.1 $^{k}$ | 7.1 ± 0.1 $^{f}$ | 7.8 ± 0.1 $^{i}$ | 8.1 ± 0.2 $^{f}$ | 129.7 ± 0.5 $^{i}$ | 5.6 ± 0.1 $^{gh}$ | 3.5 ± 0.3 $^{efg}$ | 13.0 ± 0.1 $^{cde}$ |
| S20-IV | 16.8 ± 0.3 $^{k}$ | 6.3 ± 0.1 $^{de}$ | 4.7 ± 0.2 $^{fg}$ | 15.6 ± 0.2 $^{k}$ | 105.1 ± 1.5 $^{f}$ | 6.4 ± 0.1 $^{i}$ | 3.6 ± 0.1 $^{fg}$ | 10.1 ± 0.1 $^{b}$ |
| EM20-I | 12.7 ± 0.3 $^{hi}$ | 6.4 ± 0.3 $^{e}$ | 4.5 ± 0.1 $^{f}$ | 4.7 ± 0.1 $^{cd}$ | 90.7 ± 1.8 $^{d}$ | 4.4 ± 0.1 $^{de}$ | 3.7 ± 0.1 $^{g}$ | 8.1 ± 0.2 $^{a}$ |
| EM20-II | 13.2 ± 0.1 $^{i}$ | 8.4 ± 0.1 $^{gh}$ | 2.5 ± 0.2 $^{c}$ | 5.2 ± 0.1 $^{de}$ | 96.5 ± 0.8 $^{e}$ | 1.3 ± 0.1 $^{a}$ | 5.8 ± 0.1 $^{j}$ | 14.5 ± 0.3 $^{de}$ |
| EM20-III | 16.7 ± 0.3 $^{k}$ | 8.2 ± 0.1 $^{g}$ | 2.9 ± 0.1 $^{d}$ | 4.8 ± 0.1 $^{cd}$ | 88.2 ± 1.0 $^{d}$ | 3.9 ± 0.1 $^{bcd}$ | 6.4 ± 0.1 $^{k}$ | 15.1 ± 0.1 $^{e}$ |
| EM20-IV | 7.5 ± 0.2 $^{bc}$ | 4.6 ± 0.3 $^{b}$ | 2.0 ± 0.1 $^{ab}$ | 5.6 ± 0.1 $^{e}$ | 102.2 ± 0.4 $^{f}$ | 4.6 ± 0.3 $^{def}$ | 2.4 ± 0.1 $^{cd}$ | 11.0 ± 0.4 $^{bc}$ |
| E+20-I | 15.3 ± 0.1 $^{j}$ | 9.3 ± 0.1 $^{i}$ | 2.7 ± 0.1 $^{cd}$ | 4.5 ± 0.1 $^{bc}$ | 95.4 ± 1.4 $^{e}$ | 3.7 ± 0.3 $^{b}$ | 1.8 ± 0.1 $^{ab}$ | 15.4 ± 0.3 $^{e}$ |
| E+20-II | 11.3 ± 0.1 $^{fg}$ | 8.7 ± 0.1 $^{h}$ | 4.9 ± 0.1 $^{g}$ | 3.6 ± 0.3 $^{a}$ | 62.3 ± 0.7 $^{b}$ | 4.4 ± 0.3 $^{cde}$ | 3.1 ± 0.1 $^{e}$ | 12.1 ± 0.1 $^{bcd}$ |
| E+20-III | 10.4 ± 0.1 $^{e}$ | 8.2 ± 0.1 $^{g}$ | 2.4 ± 0.1 $^{c}$ | 3.6 ± 0.1 $^{a}$ | 59.0 ± 0.5 $^{b}$ | 3.9 ± 0.1 $^{bcd}$ | 2.0 ± 0.1 $^{abc}$ | 11.2 ± 0.1 $^{bc}$ |
| E+20-IV | 21.1 ± 0.3 $^{l}$ | 9.2 ± 0.2 $^{i}$ | 2.4 ± 0.1 $^{c}$ | 4.0 ± 0.1 $^{ab}$ | 88.2 ± 1.8 $^{d}$ | 9.6 ± 0.5 $^{k}$ | 5.1 ± 0.1 $^{h}$ | 20.8 ± 0.3 $^{f}$ |
| EL20-I | 8.8 ± 0.6 $^{d}$ | 5.3 ± 0.1 $^{c}$ | 3.5 ± 0.1 $^{e}$ | 8.4 ± 0.4 $^{fg}$ | 172.8 ± 0.2 $^{l}$ | 5.1 ± 0.2 $^{fg}$ | 24.8 ± 0.4 $^{n}$ | 34.7 ± 2.2 $^{j}$ |
| EL20-II | 7.0 ± 0.2 $^{b}$ | 3.6 ± 0.1 $^{a}$ | 1.9 ± 0.1 $^{ab}$ | 12.2 ± 0.6 $^{i}$ | 154.0 ± 1.6 $^{j}$ | 5.9 ± 0.2 $^{hi}$ | 30.9 ± 0.5 $^{o}$ | 47.7 ± 0.5 $^{m}$ |
| EL20-III | 7.6 ± 0.1 $^{bc}$ | 3.6 ± 0.1 $^{a}$ | 2.5 ± 0.1 $^{c}$ | 11.1 ± 0.3 $^{h}$ | 150.4 ± 0.8 $^{j}$ | 9.9 ± 0.5 $^{k}$ | 19.0 ± 0.2 $^{m}$ | 38.3 ± 1.1 $^{k}$ |
| EL20-IV | 10.9 ± 0.3 $^{ef}$ | 6.0 ± 0.1 $^{d}$ | 2.5 ± 0.1 $^{c}$ | 23.3 ± 0.1 $^{l}$ | 169.6 ± 3.1 $^{l}$ | 8.8 ± 0.1 $^{j}$ | 10.3 ± 0.1 $^{l}$ | 41.4 ± 0.3 $^{l}$ |
| PM20-I | 9.2 ± 0.2 $^{d}$ | 3.2 ± 0.1 $^{a}$ | 2.1 ± 0.1 $^{b}$ | 8.4 ± 0.3 $^{fg}$ | 159.7 ± 1.3 $^{k}$ | 11.0 ± 0.1 $^{l}$ | 3.1 ± 0.1 $^{ef}$ | 39.4 ± 0.2 $^{kl}$ |
| PM20-II | 9.1 ± 0.1 $^{d}$ | 3.5 ± 0.1 $^{a}$ | 1.8 ± 0.1 $^{a}$ | 8.1 ± 0.1 $^{f}$ | 88.5 ± 0.5 $^{d}$ | 6.2 ± 0.1 $^{hi}$ | 2.5 ± 0.1 $^{d}$ | 28.6 ± 0.3 $^{gh}$ |
| PM20-III | 4.6 ± 0.2 $^{a}$ | 3.5 ± 0.1 $^{a}$ | 1.9 ± 0.1 $^{ab}$ | 8.4 ± 0.1 $^{fg}$ | 81.0 ± 1.1 $^{c}$ | 4.4 ± 0.1 $^{cde}$ | 1.5 ± 0.1 $^{a}$ | 27.3 ± 0.6 $^{g}$ |
| PM20-IV | 10.5 ± 0.6 $^{ef}$ | 3.3 ± 0.2 $^{a}$ | 2.4 ± 0.1 $^{c}$ | 8.5 ± 0.1 $^{fg}$ | 101.1 ± 1.0 $^{f}$ | 6.2 ± 0.2 $^{hi}$ | 2.1 ± 0.1 $^{bcd}$ | 30.8 ± 2.8 $^{hi}$ |

"-" not detected. Abbreviations: CS20−2020 vintage Cabernet Sauvignon sample prior storage; 20−vintage year 2020; S−stainless steel tank; EM−wooden barrel with excellent medium toasting; E+−wooden barrel with excellent medium plus toasting; EL−wooden barrel with excellent medium long toasting; PM−wooden barrel with premium medium toasting; I, II, III, IV−sampling after 3, 6, 9 and 12 months of ageing, respectively. Different superscript letters (a–p) in the same column indicate statistical difference determined by ANOVA, Fisher's (LSD) test with $p<0.05$.

**Table S8.** Ester concentrations (µg/L) in the aromatic profile of 2021 vintage Cabernet Sauvignon and samples obtained during 12-month storage in different vessels.

| Sample | Ethyl hexanoate | Ethyl 4-hydroxybutanoate | Isoamyl lactate | Diethyl succinate | Ethyl octanoate | Phenethyl acetate | Ethyl decanoate | Ethyl cinnamate |
|---|---|---|---|---|---|---|---|---|
| CS21 | 304.7 ± 1.1 $^{k}$ | 69.2 ± 1.5 $^{m}$ | - | 579.5 ± 0.9 $^{ab}$ | 396.2 ± 0.5 $^{m}$ | 51.0 ± 0.6 $^{ij}$ | 38.7 ± 0.8 $^{i}$ | - |
| S21-I | 299.9 ± 6.0 $^{k}$ | 15.7 ± 0.2 $^{a}$ | - | 557.4 ± 13.1 $^{a}$ | 378.1 ± 1.1 $^{l}$ | 73.0 ± 0.4 $^{l}$ | 48.3 ± 1.2 $^{l}$ | - |
| S21-II | 275.8 ± 7.0 $^{j}$ | 35.4 ± 1.1 $^{f}$ | - | 608.2 ± 4.6 $^{cd}$ | 377.9 ± 7.5 $^{l}$ | 45.4 ± 0.7 $^{efg}$ | 43.0 ± 0.3 $^{j}$ | - |
| S21-III | 128.8 ± 2.0 $^{bc}$ | 27.5 ± 0.1 $^{d}$ | - | 761.6 ± 8.8 $^{i}$ | 158.9 ± 3.8 $^{b}$ | 47.1 ± 1.0 $^{fgh}$ | 18.0 ± 0.3 $^{c}$ | - |
| S21-IV | 95.9 ± 0.1 $^{a}$ | 25.2 ± 0.2 $^{c}$ | - | 804.1 ± 1.7 $^{k}$ | 128.4 ± 0.2 $^{a}$ | 34.2 ± 0.1 $^{c}$ | 11.8 ± 0.1 $^{b}$ | - |
| EM21-I | 345.9 ± 4.5 $^{l}$ | 51.2 ± 0.2 $^{l}$ | 21.0 ± 1.0 $^{b}$ | 790.0 ± 5.2 $^{jk}$ | 466.6 ± 4.1 $^{n}$ | 51.4 ± 0.3 $^{ij}$ | 43.7 ± 0.6 $^{jk}$ | - |
| EM21-II | 174.4 ± 1.0 $^{f}$ | 31.9 ± 0.1 $^{e}$ | 35.2 ± 0.2 $^{e}$ | 715.3 ± 18.8 $^{gh}$ | 290.7 ± 4.2 $^{hi}$ | 54.5 ± 1.9 $^{k}$ | 33.4 ± 0.3 $^{f}$ | - |
| EM21-III | 192.8 ± 2.9 $^{g}$ | 46.5 ± 1.0 $^{i}$ | 38.4 ± 0.5 $^{fg}$ | 633.2 ± 6.4 $^{ef}$ | 309.1 ± 3.0 $^{jk}$ | 36.6 ± 1.6 $^{c}$ | 33.7 ± 0.5 $^{f}$ | - |
| EM21-IV | 117.6 ± 2.5 $^{b}$ | 48.7 ± 0.6 $^{jk}$ | 46.0 ± 0.9 $^{ij}$ | 786.2 ± 2.7 $^{ij}$ | 232.2 ± 2.1 $^{e}$ | 31.1 ± 0.2 $^{b}$ | 29.2 ± 0.1 $^{e}$ | - |
| E+21-I | 417.5 ± 7.3 $^{n}$ | 51.3 ± 0.7 $^{l}$ | 15.9 ± 0.3 $^{a}$ | 573.2 ± 11.3 $^{ab}$ | 317.5 ± 3.4 $^{k}$ | 36.3 ± 0.9 $^{c}$ | 17.7 ± 0.3 $^{c}$ | - |
| E+21-II | 219.7 ± 0.5 $^{i}$ | 30.3 ± 0.2 $^{e}$ | 21.3 ± 0.3 $^{b}$ | 705.3 ± 10.4 $^{gh}$ | 279.3 ± 5.8 $^{g}$ | 47.7 ± 1.3 $^{gh}$ | 34.8 ± 1.7 $^{fg}$ | - |
| E+21-III | 214.0 ± 3.0 $^{hi}$ | 48.1 ± 0.4 $^{ij}$ | 42.2 ± 1.8 $^{h}$ | 775.9 ± 4.0 $^{ij}$ | 266.2 ± 1.9 $^{f}$ | 43.2 ± 1.4 $^{e}$ | 43.8 ± 1.0 $^{jk}$ | - |
| E+21-IV | 201.9 ± 1.6 $^{gh}$ | 50.3 ± 0.4 $^{kl}$ | 48.0 ± 0.1 $^{j}$ | 802.4 ± 1.3 $^{k}$ | 223.9 ± 1.9 $^{e}$ | 48.9 ± 0.8 $^{hi}$ | 53.7 ± 0.1 $^{m}$ | - |
| EL21-I | 371.8 ± 0.7 $^{m}$ | 21.9 ± 0.1 $^{b}$ | 22.9 ± 0.5 $^{b}$ | 631.6 ± 4.3 $^{ef}$ | 521.8 ± 3.7 $^{o}$ | 44.7 ± 1.1 $^{ef}$ | 36.5 ± 0.9 $^{gh}$ | - |
| EL21-II | 149.2 ± 1.6 $^{e}$ | 31.0 ± 0.9 $^{e}$ | 30.6 ± 0.8 $^{cd}$ | 697.4 ± 8.4 $^{g}$ | 265.5 ± 0.8 $^{f}$ | 52.1 ± 1.7 $^{jk}$ | 37.0 ± 1.2 $^{hi}$ | - |
| EL21-III | 143.4 ± 0.6 $^{de}$ | 27.7 ± 0.1 $^{d}$ | 36.9 ± 0.5 $^{efg}$ | 725.4 ± 2.3 $^{h}$ | 204.3 ± 3.2 $^{d}$ | 35.2 ± 0.2 $^{c}$ | 12.5 ± 0.2 $^{b}$ | - |
| EL21-IV | 136.1 ± 2.9 $^{cd}$ | 32.0 ± 0.6 $^{e}$ | 44.4 ± 0.7 $^{hi}$ | 849.2 ± 0.3 $^{l}$ | 176.7 ± 1.1 $^{c}$ | 45.4 ± 1.1 $^{efg}$ | 9.7 ± 0.3 $^{a}$ | - |
| PM21-I | 264.4 ± 12.4 $^{j}$ | 37.8 ± 0.3 $^{g}$ | 29.9 ± 0.5 $^{c}$ | 612.6 ± 5.3 $^{de}$ | 283.3 ± 4.1 $^{gh}$ | 46.7 ± 0.7 $^{fgh}$ | 45.4 ± 0.2 $^{k}$ | - |
| PM21-II | 196.6 ± 5.3 $^{g}$ | 51.8 ± 1.1 $^{l}$ | 32.5 ± 1.1 $^{d}$ | 644.0 ± 3.7 $^{f}$ | 298.2 ± 5.6 $^{ij}$ | 40.2 ± 0.8 $^{d}$ | 50.2 ± 0.4 $^{l}$ | - |
| PM21-III | 166.1 ± 2.4 $^{f}$ | 36.3 ± 1.2 $^{fg}$ | 36.2 ± 0.5 $^{ef}$ | 646.2 ± 12.0 $^{f}$ | 233.6 ± 5.2 $^{e}$ | 29.3 ± 0.1 $^{b}$ | 28.0 ± 0.8 $^{e}$ | - |
| PM21-IV | 133.8 ± 0.7 $^{cd}$ | 41.0 ± 0.1 $^{h}$ | 39.2 ± 0.8 $^{g}$ | 693.9 ± 1.8 $^{g}$ | 229.6 ± 5.3 $^{e}$ | 25.5 ± 0.2 $^{a}$ | 23.4 ± 0.1 $^{d}$ | - |

**Table S8.** Continue.

| Sample | Ethyl vanillate | Ethyl laurate | Methyl dihydrojasmonate | Ethyl myristate | Ethyl palmitate | Ethyl linoleate | Ethyl oleate | Ethyl stearate |
|---|---|---|---|---|---|---|---|---|
| CS21 | $2.0 \pm 0.1^{fg}$ | $8.7 \pm 0.6^{c}$ | $3.9 \pm 0.3^{j}$ | $14.8 \pm 0.1^{a}$ | $76.9 \pm 0.2^{c}$ | $3.7 \pm 0.1^{bc}$ | $4.4 \pm 0.1^{a}$ | $4.9 \pm 0.1^{a}$ |
| S21-I | $1.5 \pm 0.1^{c}$ | $11.4 \pm 0.2^{ghi}$ | $3.9 \pm 0.1^{j}$ | $22.5 \pm 0.4^{e}$ | $163.8 \pm 1.2^{l}$ | $8.6 \pm 0.2^{g}$ | $4.5 \pm 0.1^{a}$ | $14.4 \pm 0.3^{fg}$ |
| S21-II | $1.4 \pm 0.1^{c}$ | $10.7 \pm 0.1^{efg}$ | $2.6 \pm 0.1^{f}$ | $24.2 \pm 0.5^{ef}$ | $118.8 \pm 1.6^{i}$ | $12.2 \pm 0.1^{i}$ | $4.6 \pm 0.1^{a}$ | $15.1 \pm 0.1^{fg}$ |
| S21-III | $1.6 \pm 0.1^{cd}$ | $8.4 \pm 0.4^{c}$ | $2.1 \pm 0.1^{cd}$ | $18.7 \pm 0.3^{d}$ | $75.0 \pm 0.1^{c}$ | $28.3 \pm 0.1^{m}$ | $5.0 \pm 0.1^{a}$ | $12.9 \pm 0.1^{de}$ |
| S21-IV | $1.5 \pm 0.1^{cd}$ | $7.1 \pm 0.1^{ab}$ | $1.5 \pm 0.1^{a}$ | $18.1 \pm 0.1^{cd}$ | $47.3 \pm 0.2^{a}$ | $33.4 \pm 0.3^{o}$ | $10.5 \pm 0.1^{cd}$ | $12.3 \pm 0.1^{cd}$ |
| EM21-I | $2.5 \pm 0.1^{h}$ | $8.1 \pm 0.4^{bc}$ | $3.8 \pm 0.2^{ij}$ | $16.0 \pm 0.8^{ab}$ | $81.2 \pm 0.8^{d}$ | $8.1 \pm 0.1^{fg}$ | $5.1 \pm 0.2^{a}$ | $15.7 \pm 0.3^{gh}$ |
| EM21-II | $3.0 \pm 0.1^{i}$ | $10.2 \pm 0.1^{def}$ | $2.9 \pm 0.1^{g}$ | $18.2 \pm 0.3^{cd}$ | $92.1 \pm 0.6^{f}$ | $22.2 \pm 0.1^{k}$ | $11.2 \pm 0.5^{d}$ | $13.8 \pm 0.1^{ef}$ |
| EM21-III | $4.1 \pm 0.1^{j}$ | $15.1 \pm 0.3^{k}$ | $3.4 \pm 0.2^{h}$ | $56.0 \pm 1.6^{k}$ | $121.0 \pm 1.1^{i}$ | $35.5 \pm 0.3^{p}$ | $12.4 \pm 0.1^{e}$ | $26.7 \pm 0.1^{k}$ |
| EM21-IV | $4.8 \pm 0.1^{k}$ | $18.4 \pm 0.2^{l}$ | $3.9 \pm 0.1^{j}$ | $61.8 \pm 0.4^{m}$ | $138.6 \pm 0.5^{j}$ | $43.5 \pm 0.1^{q}$ | $15.2 \pm 0.2^{f}$ | $28.7 \pm 1.2^{l}$ |
| E+21-I | $1.4 \pm 0.1^{c}$ | $7.0 \pm 0.1^{a}$ | $3.3 \pm 0.2^{h}$ | $26.7 \pm 0.2^{g}$ | $87.8 \pm 0.3^{e}$ | $5.8 \pm 0.2^{e}$ | $5.0 \pm 0.1^{a}$ | $16.6 \pm 0.1^{h}$ |
| E+21-II | $2.1 \pm 0.1^{g}$ | $12.2 \pm 0.3^{i}$ | $3.8 \pm 0.1^{ij}$ | $24.9 \pm 0.2^{f}$ | $97.3 \pm 0.2^{g}$ | $5.5 \pm 0.1^{e}$ | $21.5 \pm 0.4^{h}$ | $24.5 \pm 0.3^{j}$ |
| E+21-III | $4.0 \pm 0.1^{j}$ | $19.3 \pm 0.6^{l}$ | $4.7 \pm 0.1^{k}$ | $59.9 \pm 0.7^{l}$ | $135.7 \pm 1.4^{j}$ | $4.5 \pm 0.1^{d}$ | $24.5 \pm 0.1^{i}$ | $39.7 \pm 0.8^{m}$ |
| E+21-IV | $5.2 \pm 0.1^{l}$ | $22.7 \pm 0.1^{m}$ | $5.3 \pm 0.1^{l}$ | $70.4 \pm 0.8^{n}$ | $146.0 \pm 0.5^{k}$ | $4.0 \pm 0.1^{cd}$ | $32.7 \pm 0.2^{j}$ | $46.5 \pm 1.2^{n}$ |
| EL21-I | $1.8 \pm 0.1^{ef}$ | $11.9 \pm 0.8^{hi}$ | $3.5 \pm 0.2^{hi}$ | $42.0 \pm 0.9^{i}$ | $86.7 \pm 0.5^{e}$ | $3.2 \pm 0.1^{b}$ | $11.5 \pm 0.8^{de}$ | $12.7 \pm 0.3^{cde}$ |
| EL21-II | $1.7 \pm 0.1^{de}$ | $11.0 \pm 0.4^{fgh}$ | $2.5 \pm 0.1^{ef}$ | $28.3 \pm 1.1^{g}$ | $88.8 \pm 1.1^{e}$ | $7.7 \pm 0.2^{f}$ | $19.4 \pm 0.1^{g}$ | $14.7 \pm 0.2^{fg}$ |
| EL21-III | $1.5 \pm 0.1^{c}$ | $9.7 \pm 0.1^{d}$ | $2.4 \pm 0.1^{def}$ | $41.5 \pm 0.3^{i}$ | $107.1 \pm 1.0^{h}$ | $8.1 \pm 0.1^{fg}$ | $24.6 \pm 1.1^{i}$ | $21.7 \pm 0.2^{i}$ |
| EL21-IV | $1.4 \pm 0.1^{c}$ | $8.8 \pm 0.3^{c}$ | $1.9 \pm 0.1^{bc}$ | $46.8 \pm 0.1^{j}$ | $136.2 \pm 2.1^{j}$ | $11.5 \pm 0.1^{h}$ | $31.9 \pm 0.6^{j}$ | $25.2 \pm 0.5^{j}$ |
| PM21-I | $2.7 \pm 0.1^{h}$ | $15.2 \pm 0.1^{k}$ | $8.0 \pm 0.1^{m}$ | $16.9 \pm 0.1^{bc}$ | $69.9 \pm 1.3^{b}$ | $1.5 \pm 0.1^{a}$ | $9.1 \pm 0.3^{b}$ | $11.4 \pm 0.2^{bc}$ |
| PM21-II | $1.8 \pm 0.1^{ef}$ | $13.1 \pm 0.1^{j}$ | $2.2 \pm 0.1^{cde}$ | $27.2 \pm 0.1^{g}$ | $70.3 \pm 1.2^{b}$ | $23.4 \pm 0.8^{l}$ | $9.8 \pm 0.1^{bc}$ | $10.4 \pm 0.2^{b}$ |
| PM21-III | $1.1 \pm 0.1^{b}$ | $9.9 \pm 0.3^{de}$ | $2.3 \pm 0.1^{def}$ | $34.0 \pm 0.4^{h}$ | $100.1 \pm 0.4^{g}$ | $21.4 \pm 0.1^{j}$ | $10.7 \pm 0.1^{cd}$ | $14.5 \pm 0.2^{fg}$ |
| PM21-IV | $0.7 \pm 0.1^{a}$ | $8.5 \pm 0.1^{c}$ | $1.7 \pm 0.1^{ab}$ | $40.3 \pm 0.2^{i}$ | $106.8 \pm 2.1^{h}$ | $31.9 \pm 0.3^{n}$ | $9.4 \pm 0.2^{b}$ | $15.6 \pm 0.4^{gh}$ |

"-" not detected. Abbreviations: CS21−2021 vintage Cabernet Sauvignon sample prior storage; 21−vintage year 2021; S−stainless steel tank; EM−wooden barrel with excellent medium toasting; E+−wooden barrel with excellent medium plus toasting; EL−wooden barrel with excellent medium long toasting; PM−wooden barrel with premium medium toasting; I, II, III, IV−sampling after 3, 6, 9 and 12 months of ageing, respectively. Different superscript letters (a−o) in the same column indicate statistical difference determined by ANOVA, Fisher's (LSD) test with $p<0.05$.

**Table S9.** The results of descriptive analysis (points from 0 to 10 for each property or aromatic note) obtained by trained panellists for 2020 vintage Cabernet Sauvignon and samples obtained during 12-month storage in different vessels.

| Property/ aromatic note | CS20 | S20-I | S20-II | S20-III | S20-IV | EM20-I | EM20-II | EM20-III | EM20-IV | E+20-I | E+20-II | E+20-III | E+20-IV | EL20-I | EL20-II | EL20-III | EL20-IV | PM20-I | PM20-II | PM20-III | PM20-IV |
|---|---|---|---|---|---|---|---|---|---|---|---|---|---|---|---|---|---|---|---|---|---|
| Body | 6.0 | 7.0 | 6.7 | 5.3 | 6.3 | 7.0 | 7.3 | 6.0 | 6.0 | 6.7 | 6.3 | 7.3 | 7.3 | 6.3 | 7.3 | 7.0 | 7.3 | 5.7 | 6.7 | 7.0 | 7.7 |
| Sweetness | 2.0 | 2.7 | 2.0 | 1.0 | 2.0 | 1.3 | 3.3 | 2.0 | 2.7 | 1.0 | 2.3 | 2.7 | 2.0 | 3.3 | 1.3 | 2.3 | 1.3 | 2.3 | 1.7 | 2.0 | 2.0 |
| Astringency | 3.3 | 3.0 | 2.7 | 2.7 | 6.7 | 4.0 | 2.7 | 3.3 | 6.0 | 4.7 | 3.7 | 2.7 | 5.3 | 5.3 | 4.0 | 2.3 | 3.0 | 5.0 | 6.0 | 3.0 | 2.0 |
| Black currant | 7.3 | 7.0 | 6.0 | 9.0 | 7.0 | 4.7 | 7.0 | 4.7 | 5.0 | 4.0 | 7.3 | 5.7 | 3.3 | 4.7 | 5.0 | 6.0 | 5.3 | 3.0 | 4.3 | 7.0 | 4.3 |
| Black cherry | 6.3 | 7.0 | 8.7 | 5.0 | 8.0 | 9.7 | 7.0 | 8.7 | 7.3 | 7.3 | 8.3 | 8.3 | 7.0 | 7.3 | 7.0 | 8.7 | 8.0 | 4.3 | 7.7 | 7.3 | 8.3 |
| Blackberry | 5.7 | 6.7 | 6.7 | 7.7 | 7.7 | 8.3 | 5.0 | 6.7 | 6.3 | 6.0 | 8.7 | 6.3 | 4.7 | 6.3 | 6.0 | 9.3 | 6.7 | 2.3 | 7.3 | 7.0 | 7.7 |
| Cherry | 3.7 | 3.0 | 2.7 | 1.7 | 4.3 | 1.0 | 7.7 | 4.7 | 5.0 | 3.0 | 2.7 | 4.7 | 5.0 | 2.3 | 4.7 | 1.3 | 7.0 | 3.3 | 2.7 | 1.7 | 1.7 |
| Dry plum | 4.7 | 2.3 | 4.0 | 3.7 | 6.0 | 3.7 | 2.7 | 5.7 | 3.0 | 5.7 | 6.7 | 4.7 | 2.3 | 6.3 | 4.3 | 2.7 | 3.3 | 1.7 | 1.7 | 5.7 | 4.0 |
| Smoke | - | - | 1.0 | - | - | 1.0 | - | 1.0 | 1.0 | 1.7 | - | - | 5.0 | 1.0 | 2.3 | 2.0 | - | 1.7 | 4.3 | 2.3 | 1.0 |
| Plum jam | 6.7 | 5.7 | 7.3 | 5.3 | 8.0 | 6.0 | 5.3 | 7.7 | 4.0 | 6.3 | 9.0 | 6.3 | 5.5 | 5.7 | 4.0 | 4.7 | 6.3 | 3.7 | 4.3 | 5.7 | 6.7 |
| Bell pepper | 2.7 | 2.0 | 3.0 | 1.0 | 1.3 | 5.7 | 2.7 | 4.3 | 3.3 | 4.3 | 3.7 | 2.3 | 4.3 | 2.0 | 4.3 | 5.0 | 3.0 | 2.3 | 2.7 | 2.7 | 2.7 |
| Coffee | - | - | 1.7 | 3.0 | 5.3 | 2.3 | - | 5.7 | 6.0 | 4.3 | 1.0 | 3.0 | 4.7 | 6.0 | 3.3 | 2.7 | - | 4.0 | 4.3 | 8.0 | 5.7 |
| Chocolate | - | - | 3.5 | 2.0 | 5.0 | 3.3 | - | 3.3 | 1.7 | 3.0 | 2.0 | - | 1.0 | 5.7 | 6.7 | 4.3 | - | 1.0 | 1.7 | 4.7 | 6.0 |
| Cedar wood | 2.7 | 2.7 | 8.3 | 8.0 | 9.0 | 9.0 | 3.3 | 8.0 | 5.0 | 7.7 | 8.0 | 3.3 | 3.0 | 4.3 | 9.0 | 9.7 | 3.0 | 1.3 | 4.7 | 5.0 | 8.3 |
| Oak wood | - | - | 1.7 | 1.5 | 1.7 | 2.0 | - | 1.7 | 3.0 | 1.3 | 1.0 | - | 2.7 | 1.0 | 1.0 | 1.0 | - | 2.0 | - | 1.7 | 1.0 |
| Graphite | 1.3 | 1.3 | 2.7 | 1.3 | 1.3 | 1.0 | 1.7 | 1.0 | 2.3 | 1.0 | 1.0 | 1.5 | 1.0 | 1.0 | 2.0 | 2.3 | 1.7 | 1.0 | 1.3 | 2.3 | 1.0 |
| Leather | - | - | - | 2.0 | 2.7 | 1.0 | - | 1.5 | 5.3 | 1.7 | - | - | 8.3 | 6.0 | 2.0 | 1.0 | - | 9.0 | 3.3 | 2.3 | 1.5 |
| Pepper | 2.3 | 2.3 | 3.7 | 5.0 | 3.3 | 2.7 | 3.0 | 2.0 | 6.3 | 4.3 | 3.3 | 3.3 | 3.0 | 5.7 | 3.7 | 2.0 | 3.0 | 2.3 | 4.3 | 3.0 | 5.0 |
| Flowers | 2.3 | 2.3 | 2.0 | 3.0 | 3.7 | 5.7 | 1.7 | 2.3 | - | 5.0 | 5.7 | 1.5 | - | 2.0 | 2.0 | 6.3 | 1.7 | - | 1.5 | 1.0 | 4.0 |

"-" not detected. Abbreviations: CS20−2020 vintage Cabernet Sauvignon sample prior storage; 20−vintage year 2020; S−stainless steel tank; EM−wooden barrel with excellent medium toasting; E+−wooden barrel with excellent medium plus toasting; EL−wooden barrel with excellent medium long toasting; PM−wooden barrel with premium medium toasting; I, II, III, IV−sampling after 3, 6, 9 and 12 months of ageing, respectively.

**Table S10.** The results of descriptive analysis (points from 0 to 10 for each property or aromatic note) obtained by trained panellists for 2021 vintage Cabernet Sauvignon and samples obtained during 12-month storage in different vessels.

| Property/ aromatic note | CS21 | S21-I | S21-II | S21-III | S21-IV | EM21-I | EM21-II | EM21-III | EM21-IV | E+21-I | E+21-II | E+21-III | E+21-IV | EL21-I | EL21-II | EL21-III | EL21-IV | PM21-I | PM21-II | PM21-III | PM21-IV |
|---|---|---|---|---|---|---|---|---|---|---|---|---|---|---|---|---|---|---|---|---|---|
| Body | 10.6 | 11.7 | 7.6 | 8.6 | 9.3 | 11.0 | 11.0 | 9.1 | 8.5 | 8.6 | 9.5 | 10.3 | 9.7 | 8.5 | 8.6 | 9.3 | 9.3 | 8.9 | 7.4 | 8.1 | 9.1 |
| Sweetness | 2.0 | 3.1 | 2.4 | 3.8 | 4.6 | 4.9 | 3.0 | 2.3 | 1.3 | 2.1 | 6.0 | 4.6 | 1.8 | 1.8 | 1.9 | 1.5 | 2.5 | 3.0 | 3.1 | 1.6 | 3.1 |
| Astringency | 4.6 | 8.0 | 10.0 | 9.1 | 8.8 | 6.2 | 12.2 | 12.6 | 11.6 | 10.3 | 6.0 | 8.6 | 11.5 | 10.8 | 9.7 | 8.9 | 7.8 | 9.9 | 10.9 | 10.5 | 6.7 |
| Black currant | 9.3 | 14.8 | 5.7 | 3.8 | 5.1 | 4.0 | 10.4 | 4.6 | 5.4 | 5.1 | 6.0 | 10.3 | 2.8 | 4.9 | 6.6 | 5.9 | 9.3 | 2.5 | 2.6 | 4.4 | 7.9 |
| Black cherry | 15.2 | 11.7 | 9.5 | 12.5 | 10.6 | 9.7 | 14.0 | 8.0 | 10.3 | 9.4 | 9.5 | 13.1 | 9.7 | 12.1 | 10.1 | 8.5 | 10.8 | 7.4 | 10.5 | 9.7 | 9.8 |
| Blackberry | 15.9 | 11.1 | 8.1 | 10.1 | 9.3 | 10.6 | 10.4 | 6.9 | 4.5 | 7.3 | 8.4 | 14.3 | 8.8 | 9.9 | 8.6 | 8.5 | 9.8 | 4.5 | 7.0 | 8.9 | 8.7 |
| Cherry | 7.3 | 7.4 | 2.9 | 2.2 | 2.3 | 6.2 | 6.1 | 3.4 | 2.7 | 1.7 | 3.2 | 4.6 | 3.7 | 2.7 | 2.7 | 10.4 | 5.4 | 5.0 | 6.1 | 0.0 | 2.4 |
| Dry plum | 7.3 | 3.1 | 7.6 | 3.4 | 6.0 | 5.7 | 8.5 | 2.9 | 7.6 | 6.4 | 7.6 | 3.4 | 5.1 | 6.7 | 4.3 | 7.8 | 8.8 | 4.0 | 2.6 | 6.4 | 7.5 |
| Smoke | 0.0 | 0.0 | 0.0 | 1.4 | 1.4 | 0.0 | 0.0 | 0.0 | 1.3 | 1.3 | 0.0 | 0.0 | 0.0 | 0.0 | 1.2 | 0.0 | 0.0 | 0.0 | 1.3 | 1.2 | 0.0 |
| Plum jam | 7.3 | 4.9 | 7.6 | 5.8 | 6.0 | 7.5 | 6.7 | 4.0 | 3.6 | 5.1 | 6.4 | 6.9 | 9.7 | 5.4 | 7.4 | 8.5 | 7.8 | 7.4 | 7.4 | 8.1 | 7.9 |
| Bell pepper | 3.3 | 8.6 | 4.8 | 7.2 | 3.2 | 6.2 | 5.5 | 5.1 | 8.5 | 3.9 | 2.8 | 9.1 | 2.8 | 2.2 | 2.7 | 1.5 | 6.9 | 4.5 | 2.2 | 1.2 | 5.5 |
| Coffee | 0.0 | 0.0 | 2.9 | 8.2 | 5.6 | 2.6 | 0.0 | 5.1 | 7.6 | 6.9 | 3.6 | 0.0 | 4.1 | 4.0 | 4.7 | 3.7 | 0.0 | 7.4 | 8.3 | 6.8 | 2.4 |
| Chocolate | 0.0 | 0.0 | 3.3 | 3.4 | 4.6 | 2.0 | 0.0 | 3.4 | 4.9 | 6.9 | 2.4 | 0.0 | 2.8 | 3.6 | 9.7 | 2.2 | 1.5 | 3.0 | 7.0 | 8.5 | 4.7 |
| Cedar wood | 7.3 | 4.3 | 4.8 | 7.7 | 6.5 | 9.3 | 5.5 | 6.3 | 8.5 | 11.2 | 10.3 | 4.0 | 1.8 | 10.3 | 8.6 | 9.3 | 8.3 | 3.0 | 10.1 | 7.9 | 9.8 |
| Oak wood | 0.0 | 0.0 | 1.4 | 1.4 | 2.1 | 2.6 | 0.0 | 2.3 | 1.8 | 3.4 | 1.2 | 0.0 | 3.2 | 5.4 | 2.3 | 0.0 | 0.0 | 3.0 | 0.0 | 1.2 | 1.2 |
| Graphite | 3.3 | 2.5 | 3.8 | 3.8 | 3.7 | 4.9 | 1.8 | 1.7 | 3.1 | 3.4 | 2.4 | 3.4 | 4.6 | 2.0 | 2.3 | 2.2 | 2.5 | 3.0 | 3.5 | 6.0 | 2.8 |
| Leather | 0.0 | 0.0 | 11.4 | 1.4 | 2.1 | 1.3 | 0.0 | 14.9 | 2.2 | 1.3 | 3.0 | 0.0 | 12.4 | 4.5 | 1.8 | 3.0 | 1.5 | 14.4 | 7.9 | 1.6 | 1.2 |
| Pepper | 6.6 | 6.8 | 6.2 | 6.2 | 5.6 | 3.5 | 4.9 | 7.4 | 5.4 | 3.9 | 4.4 | 4.6 | 5.5 | 4.9 | 3.5 | 2.2 | 5.4 | 6.4 | 2.0 | 3.6 | 2.8 |
| Flowers | 0.0 | 1.9 | 0.0 | 0.0 | 3.2 | 1.8 | 0.0 | 0.0 | 1.3 | 1.7 | 7.6 | 2.9 | 0.0 | 0.0 | 3.1 | 6.3 | 2.5 | 3.0 | 0.0 | 4.4 | 6.7 |

“-“ not detected. Abbreviations: CS21−2021 vintage Cabernet Sauvignon sample prior storage; 21−vintage year 2021; S−stainless steel tank; EM−wooden barrel with excellent medium toasting; E+−wooden barrel with excellent medium plus toasting; EL−wooden barrel with excellent medium long toasting; PM−wooden barrel with premium medium toasting; I, II, III, IV−sampling after 3, 6, 9 and 12 months of ageing, respectively.

**Table S11.** Main information of aroma compounds identified in 2020 and 2021 vintage Cabernet Sauvignon and samples obtained during 12-month ageing in different vessels.

| Compound | RT[a] | RI[b] | *m/z*[c] | OTH[d] | OD[e] |
|---|---|---|---|---|---|
| Acids | | | | | |
| Acetic acid | 3.6218 | 722 | 60 | 20,000.0 [34] | vinegar |
| Hexanoic acid | 19.5182 | 1011 | 73-116 | 800.0 [36] | fatty |
| Decanoic acid | 38.1283 | 1380 | 129-172 | 1,000.0 [39] | fatty |
| Lauric acid | 42.1492 | 1562 | 157-200 | 5.0 [45] | fatty |
| Myristic acid | 44.8136 | 1756 | 185-228 | NF | fatty |
| Palmitic acid | 47.4374 | 1997 | 213-256 | NF | fatty |
| Alcohols | | | | | |
| Isoamyl alcohol | 4.0111 | 743 | 70-88 | 60,000.0 [39] | fruity |
| 2,3-butanediol | 6.1971 | 819 | 45-90 | 4,500.0 [36] | fruity |
| 1-hexanol | 9.4455 | 869 | 84-102 | 1,100.0 [36] | green |
| 1-heptanol | 16.7968 | 974 | 56-116 | 20.0 [36] | green |
| Methionol | 17.2600 | 980 | 88-106 | 1,000.0 [39] | sulphurous |
| 2-ethyl-1-hexanol | 20.6394 | 1031 | 57-130 | 75.0 [34] | citrus |
| Benzyl alcohol | 20.7204 | 1032 | 79-108 | 100.0 [36] | fruity |
| 1-octanol | 23.1248 | 1072 | 112-130 | NF | green |
| 2-phenylethanol | 25.3993 | 1106 | 91-122 | 1,000.0 [36] | floral |
| Carbonyl compounds | | | | | |
| Benzaldehyde | 15.4325 | 956 | 77-106 | 350.0 [38] | almond |
| Geranyl acetone | 39.9235 | 1446 | 176-194 | NF | floral |
| Myristaldehyde | 42.8153 | 1604 | 82-226 | NF | fatty |
| Hexyl cinnamaldehyde | 44.6917 | 1746 | 129-216 | NF | floral |
| Terpenes | | | | | |
| Linalool oxide | 23.8234 | 1083 | 71-154 | 25.0 [37] | floral |
| Linalool | 24.5951 | 1094 | 71-154 | 6.0 [38] | citrus |
| Hotrienol | 24.9201 | 1099 | 69-154 | 110.0 [46] | floral |
| β-citronellol | 31.3942 | 1232 | 138-156 | NF | citrus |
| Eugenol | 37.1456 | 1355 | 164 | 6.0 [35] | spicy clove |
| β-damascenone | 38.3070 | 1385 | 175-190 | 0.1 [37] | fruity |
| Esters | | | | | |
| Ethyl hexanoate | 18.6977 | 997 | 115-144 | 100.0 [36] | fruity |
| Ethyl 4-hydroxybutanoate | 22.3450 | 1060 | 132 | 130.0 [36] | caramel |
| Isoamyl lactate | 22.8404 | 1067 | 43-130 | NF | nutty, fruity |
| Diethyl succinate | 29.1360 | 1188 | 129-174 | 1,200.0 [36] | fruity |
| Ethyl octanoate | 29.8264 | 1201 | 127-172 | 5.0 [42] | fruity |
| Phenethyl acetate | 32.5083 | 1251 | 104-164 | 250.0 [43] | floral |
| Ethyl decanoate | 38.5506 | 1391 | 155-200 | 50.0 [36] | fruity |
| Ethyl cinnamate | 40.1914 | 1458 | 131-162 | 1.1 [37] | fruity |
| Ethyl vanillate | 42.4500 | 1581 | 151-196 | 3.0 [37] | smoke |
| Ethyl laurate | 42.5472 | 1587 | 183-228 | 500.0 [34] | fatty |
| Methyl dihydrojasmonate | 43.4652 | 1651 | 83-198 | 15.0 [34] | floral |
| Ethyl myristate | 45.1710 | 1784 | 88-256 | 180.0 [34] | fatty |
| Ethyl palmitate | 47.3155 | 1984 | 239-284 | 1,500.0 [37] | fatty |
| Ethyl linoleate | 48.9807 | 2166 | 263-308 | NF | fatty |
| Ethyl oleate | 49.0457 | 2167 | 264-310 | 870.0 [34] | fatty |
| Ethyl stearate | 49.2082 | 2185 | 267-312 | NF | fatty |
| Phenols and lactones | | | | | |
| γ-butyrolactone | 12.7354 | 915 | 86 | 2000.0 [36] | creamy, nutty |
| 4-ethylphenol | 28.4620 | 1174 | 107-122 | 140.0 [41] | smoke |
| 4-ethylguaiacol | 33.4899 | 1272 | 137-152 | 110.0 [41] | smoke |
| cis-whiskey lactone | 34.1401 | 1283 | 99-156 | 54.0 [40] | coconut |
| γ-heptalactone | 34.6437 | 1292 | 85-142 | NF | coconut, creamy |
| trans-whiskey lactone | 35.5695 | 1313 | 99-156 | 380.0 [40] | coconut, clove |
| γ-nonalactone | 37.2835 | 1358 | 85-156 | 30.0 [44] | coconut, creamy |

[a] retention time (min); [b] retention index; [c] identification ions on mass spectrum (mass-to-charge ratio); [d] odour perception threshold (μg/L); [e] odour description (main aromatic note); NF – not found.

**Disclaimer/Publisher's Note:** The statements, opinions and data contained in all publications are solely those of the individual author(s) and contributor(s) and not of MDPI and/or the editor(s). MDPI and/or the editor(s) disclaim responsibility for any injury to people or property resulting from any ideas, methods, instructions or products referred to in the content.
